# Anti-inflammatory maistemonine-class alkaloids of *Stemona japonica*

**DOI:** 10.1007/s13659-023-00372-5

**Published:** 2023-03-13

**Authors:** Cheng -Yong Tan, Bao-Bao Shi, Mei-Fen Bao, Xiang-Hai Cai

**Affiliations:** 1 State Key Laboratory of Phytochemistry and Plant Resources in West China, Kunming Institute of Botany, Chinese Academy of Sciences, Kunming, 650201 People’s Republic of China; 2 University of Chinese Academy of Sciences, Beijing, 100049 People’s Republic of China; 3 School of Pharmaceutical Sciences, South-Central MinZu University, Wuhan, 430074 People’s Republic of China

**Keywords:** *Stemona japonica*, *Stemona* alkaloids, Stemajapines A–C, Anti-inflammatory

## Abstract

Three hitherto undescribed *Stemona* alkaloids, named stemajapines A–C (**1**–**3**), along with six known alkaloids (**4**–**9**), were isolated and identified from the roots of *Stemona japonica* (Blume) Miq. (Stemonaceae). Their structures were established by the analysis of the mass data, NMR spectra, and computational chemistry. Stemjapines A and B were degraded maistemonines without spiro-lactone ring and skeletal methyl from maistemonine. Concurrence of alkaloids **1** and **2** revealed an undescribed way to form diverse *Stemona* alkaloids. Bioassay results disclosed the anti-inflammatory natural constituents stemjapines A and C with IC_50_ values of 19.7 and 13.8 *µ*M, respectively, compared to positive control dexamethasone with 11.7 *µ*M. The findings may point out a new direction of *Stemona* alkaloids inaddition to its traditional antitussive and insecticide activities.

**Supplementary Information:**

The online version contains supplementary material available at 10.1007/s13659-023-00372-5.

## Introduction

**Fig. 1:**
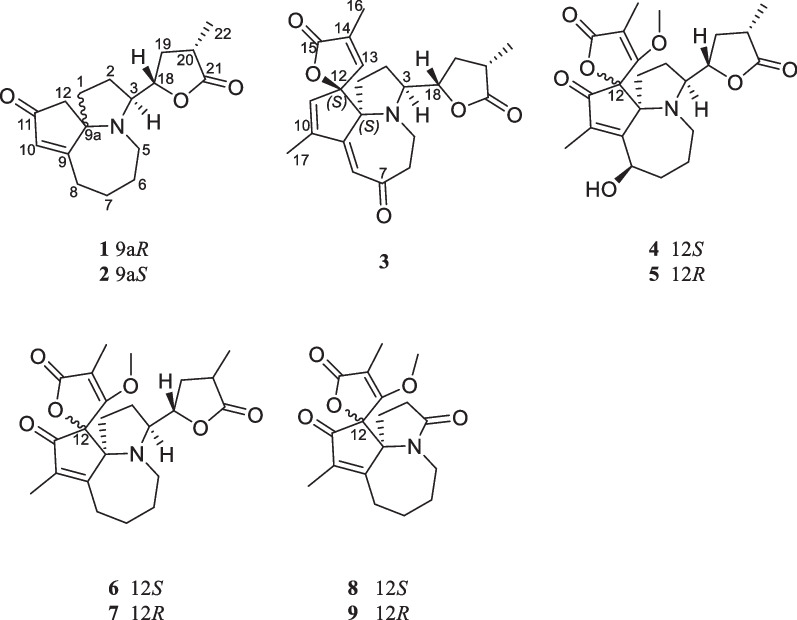
Structure of alkaloids **1**–**9**

*Stemona* species (Stemonaceae) are an abundant source of *Stemona* alkaloids [1]. More than 280 alkaloids were separated from this genus plants [2, 3]. Their fundamental ring fractions consist of a pyrrolo[1,2-*α*] azepine ring system. As a traditional Chinese medicine, *Stemona japonica* is one of Baibu resources used as an antitussive agent and insecticide. This plant is distributed in the mountainous area of Zhejiang, Jiangsu provinces, China. Previous phytochemical studies on the tuberous roots *S. japonica* led to the discovery of some *Stemona* alkaloids, which could be the characteristic components of Stemonae Radix [4–6]. Among these alkaloids, protostemonine is a main type of constituent [7, 8]. In this type, there is a special subtype, maistemonine class. This class is only several reported alkaloids with stable spiro-carbons so far, (iso)maistemonine, (iso)oxymaistemonine, (iso)stemonamide, oxomaistemonine, 3*β*-n-butylstemonamine, and 8-oxo-3*β*-n-butylstemonamine. To date, few bioactivities of these class alkaloids have been disclosed [9]. We carried out the studies on the class alkaloidal composition of this plant species. This investigation led to the isolation of three alkaloids with an unreported chiral center and skeletal carbons as well as three pairs of known homologous alkaloids (Fig. 1). Additionally, their anti-inflammatory activities were revealed.

## Results and discussion

**Fig. 2:**
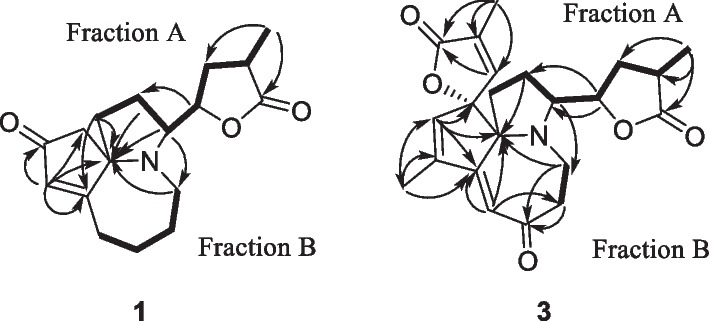
HMBC and ^1^ H-^1^ H COSY correlations of **1** and **3**

In short, 9 alkaloids were isolated and elucidated from the roots of *S. japonica* during this research. The pyrrolo[1,2-*α*]azepine type framework was the most plentiful core skeleton within the identified alkaloids and confirmed previous studies [4]. Alkaloids **1** and **2** were obtained as a white, amorphous powder. Its molecular formula was established to be C_17_H_23_NO_3_ by the positive HRESIMS spectrum from the [M + H]^+^ peak at *m / z* 312.1576 (calcd. for 312.1576). The absorption peaks of UV spectrum (MeOH) at 205, 223 and 285 nm consisted of the contemporaneously isolated stemarine B [10]. ^1^ H NMR spectrum of alkaloid **1** revealed only a methyl at *δ*_H_ 1.21 (d, *J* = 7.0 Hz, 3 H). Alkaloid **1** showed 17 carbon signals in its ^13^ C NMR spectrum, a methyl (*δ*_C_ 36.0), eight methylenes (*δ*_C_ 38.7, 27.8, 46.2, 25.2, 30.1, 31.0, 54.1, 35.2), four methines (*δ*_C_ 64.2, 130.1, 85.6, 36.0), and four quaternary carbons (*δ*_C_ 189.2, 75.5, 210.1, 182.2). Those data were very close to stemarine B except for one less methyl signal than stemarine B as shown in Table 1. So the fragment A [C_1_–C_2_–C_3_–C_18_–C_19_–C_20_(C_21_)–C_22_], and fragment B (C_5_-C_6_-C_7_-C_8_) were reflected by analysis of the ^1^ H-^1^ H COSY as well as HSQC spectra as shown in Fig. 2. The HMBC cross-peaks from H-1 (*δ*_H_ 2.04, 1.80), H-2 (*δ*_H_ 1.96) and H-5 (*δ*_H_ 3.50, 2.55) to *δ*_C_ 75.4 assign the quaternary carbon signal as C-9a. So the pyrrolo[1,2-*α*]azepine ring as well as lactone ring could be determined. Likewise, H-1 (*δ*_H_ 2.04) showed a correlation to *δ*_C_ 189.2, assigning this signal to C-9. The HMBC cross-peaks of *δ*_H_ 5.82 (H-10) to *δ*_C_ 210.1 (s), 54.0 (t), C-9 and C-9a assigned the five membered ring with *α*,*β*-unsaturated ring. Therefore, the maistemonine structure of **1** was tentatively determined as shown in Fig. 2.

**Table 1:** ^1^H (600 MHz) and ^13^C (150 MHz) NMR data of **1**–**3** in methanol-*d*_4_ (*δ* in ppm and *J* in Hz)

Position	*δ*_H_ (1)^c^	*δ*_C_ (1)	*δ*_H_ (2)^b^	*δ*_C_ (2)	*δ*_H_ (3)^c^	*δ*_C_ (3)
1	2.04^a^ 1.80, dd (12.5, 7.2)	38.7 t	1.86^a^ 1.46, dd (11.3, 7.7)	38.2 t	2.23, m 1.82^a^	41.3 t
2	1.96^a^ 1.62^a^	27.8 t	2.07^a^ 1.67^a^	26.0 t	1.83^a^ 1.28^a^	25.4 t
3	3.56, ddd (10.8, 7.4, 5.2)	64.2 d	3.11^a^	67.0 d	3.51, m	64.1 d
5	3.50, dd (15.5, 4.7) 2.55^a^	46.2 t	2.42, d (17.0) 2.33, d (17.0)	45.4 t	3.43, m	44.1 t
6	1.93^a^ 1.46, m	25.2 t	1.60^a^ 1.52^a^	31.7 t	3.17, ddd (19.3, 10.9, 4.9) 2.40, dq (19.3, 2.5)	40.7 t
7	2.05^a^ 1.23, m	30.1 t	2.13^a^ 1.14, m	31.3 t		206.8 s
8	2.83, m 2.26^a^	31.0 t	2.71^a^ 2.43^a^	29.5 t	5.92, m	119.7 d
9		189.2 s		190.0 s		165.0 s
9a		75.5 s		75.4 s		84.1 s
10	5.82, s	130.1 d	5.93, s	130.4 d		150.5 s
11		210.1 s		209.4 s	6.02, q (1.8)	133.8 d
12	2.54, dd (17.8, 0.9) 2.41, d (17.8)	54.1 t	3.09^a^ 2.33^a^	45.0 t		97.4 s
13					7.21, d (1.6)	147.4 d
14						135.2 s
15						174.6 s
16					1.98, d (1.6, 3 H)	10.8 q
17					1.98, d (1.8, 3 H)	13.7 q
18	4.22, ddd (10.8, 7.3, 5.5)	85.6 d	4.39, ddd (10.5, 7.2, 5.6)	84.2 d	3.96, ddd (10.9, 7.5, 5.4)	85.6 d
19	2.43^a^ 1.62^a^	35.2 t	2.52, ddd (12.5, 7.2, 5.6) 1.60^a^	34.9 t	2.34, ddd (12.4, 8.6, 5.4) 1.55, td (12.4, 10.8)	34.7 t
20	2.70, ddq (12.2, 8.5, 7.0)	36.0 d	2.75^a^	36.3 d	2.70, ddq (12.3, 8.6, 7.0)	35.9 d
21		182.2 s		182.3 s		181.8 s
22	1.21, d (7.0, 3 H)	15.0 q	1.22, d (7.0, 3 H)	15.3 q	1.20, d (7.0, 3 H)	15.0 q

^a^Overlap signals.^b^Recorded at 600 MHz; ^c^At 500 MHz

The ^13^C NMR spectrum of alkaloid **2** also showed 17 carbon signals, almost identical to **1**. Its molecular formula was determined by the HRESIMS to be C_17_H_23_NO_3_ from the [M + Na]^+^ peak at *m / z* 290.1756 (calcd. for 290.1756). The absorption peaks of UV spectrum (MeOH) showed absorption bands at 206, 226, and 276 nm, close to that of **1**. Unlike to **1**, **2** had the different specific rotation ([*α*] 46.7 (*c*, 0.06, MeOH)). Careful analysis of NMR spectra of **1** and **2** disclosed minor difference that signal of C-12 (*δ*_C_ 54.1) and 25.1 in **1** and was substituted by about *δ*_C_ 45.0 and 31.7 in **2** (Table 1). The HMBC correlations of *δ*_H_ 5.93 (H-10) to *δ*_C_ 190.0 (s, C-9), 209.4 (s, C-11), 45.0 (t), and 75.4 (C-9a) assigned an *α*,*β*-unsaturated five membered ring, too. Other structural fractions were same to those of **1** based on the HMBC and ^1^ H-^1^ H COSY correlations.

**Fig. 3:**
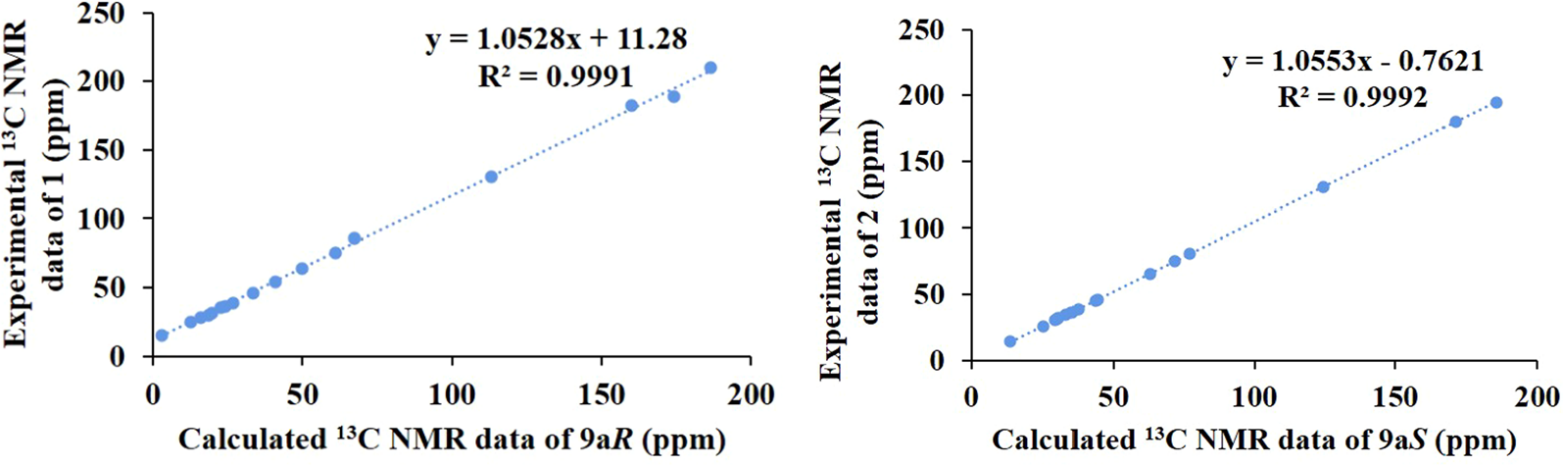
Correlation plots of experimental and ^13^ C NMR spectral data for **1** and **2**

Both **1** and **2** had completely different optical rotations ([*α*] -7 (*c*, 0.20, MeOH) for **1**; [*α*] 46.7 (*c*, 0.06, MeOH) for **2**), suggesting specific stereo-configurations. The relative configurations of **1** and **2** were reflected by their ROESY spectra in the company of its biogenetic consideration. Generally, H-18/H-20 are *β*-oriented and CH_3_-20 are *α*-oriented in *Stemona* alkaloids, the significant ROESY cross-peaks of H-1/H-2*β* and H-2*α*/H-3 suggested that H-3 is *α*-oriented and H-1 is *β*-oriented [11]. In addition to this, the obvious ROESY cross-peaks of H-20/H-1 indicated that H-20 is *β*-oriented. Thus, only the configuration of C-9a in **1** and **2** need to further elucidate. Additional extensive structural screening was performed on both epimers, and the NMR chemical shifts were calculated using the PCM solvent model in methanol at the mPW1PW91 / 6–31 + G(d, p)/M06-2X / def2SVP level of theory [10]. The ^13^ C NMR data with R^2^ values of 0.9991 for 9a***R***-**1** (**1a**) and 0.9992 for 9a***S***-**2** (**2a**) were in good agreement with their experimental values (Fig. 3), but had a lower R^2^ value in another case (Fig. S1–S11). Furthermore, calculated ECD spectra supported this prediction. On the experimental spectra, two cotton effects (CE) with alternating signals were observed, and the 9a***R*** theoretical spectrum revealed a positive cotton effect at 270 nm, which was very consistent with the experimental ECD spectra of compound **1**, while the ECD spectrum of 9a***S*** is consistent with that of compound **2** because they all have a negative cotton effect there. As a result, the absolute configurations of **1** and **2** were determined to be 9a***R*** and 9a***S***, and named as stemajapines A and B, respectively.

The ^1^H NMR spectrum of **3** showed the presence of three methyl groups (*δ*_H_ 1.20 and 1.98 × 2), two *sp*^2^ methines (*δ*_H_ 6.02 and 5.92), and two *sp*^3^ methylenes (*δ*_H_ 3.96, H-18; *δ*_H_ 3.51, H-3) (Table 1). Its ^13^C NMR spectrum showed 22 signals caused by three methyls, five methylenes, six methines, and eight quaternary carbons, including three carbonyl carbons. Compound **3** was assigned the molecular formula C_22_H_25_NO_5_ based on HRESIMS *m/z* = 406.1631 [M + Na]^+^ (calcd. for 406.1630), possessing same skeleton carbons as maistemonine or isomaistemonine. Detailed analysis of the NMR data disclosed similar substructures (Fraction A, C_1_–C_2_–C_3_–C_18_–C_19_–C_20_–C_22_) of **3** as that of maistemonine, except for absent of C-13 methoxy substitution and additional conjugated system in **3**. The UV spectrum of alkaloid **3** showed maximum absorption bands: *λ*_max_ (log *ε*) = 203 (0.40), 270 (0.26), and 322 (0.05) nm, different from those of maistemonine and isomaistemonine [12]. The HMBC correlations from H-21 (*δ*_H_ 3.96) to C-19 (*δ*_C_ 34.7, t)/20 (*δ*_C_ 35.9, d)/21 (*δ*_C_ 181.8, s) as well as H-2 (*δ*_H_ 1.83) to C-9a (*δ*_C_ 84.1) confirmed the 3-methyldihydrofuran-2-one ring (fraction A). The HMBC correlations of H-3 to the signal (*δ*_C_ 44.1) assigned it as C-5. Its proton as well as its coupling protons *δ*_H_ 3.17 (H-6) showed the HMBC correlations to new carbonyl (*δ*_C_ 206.8, s), placing the signal as C-7. A proton indicated HMBC correlations to C-7/6/9a and signal (*δ*_C_ 165.0, s), assigning the pyrrolo[1,2-*α*]azepine ring containing an *α*,*β*-unsaturated lactone. Further, the HMBC crosspeaks between *δ*_H_ 6.02 (q, *J* = 1.8 Hz) with C-9 (*δ*_C_ 165.0), C-9a, *δ*_C_ 97.4 (C-12), and between methyl protons (*δ*_H_ 1.98) with C-9/10(*δ*_C_ 150.5, s)/11(*δ*_C_ 133.8, d) confirmed the 1-en-cyclopentene fused with azepine ring. This ring without carbonyl was different from that in maistemonine. Finally, the 3-methylfuran-2-one moiety was elucidated by the HMBC correlations from H-16 (*δ*_H_ 1.98) to C-13(147.4, d)/14(135.2, s)/15(174.6, s) as well as unsaturation degrees of **3**. Therefore, the planar structure of **3** was determined.

**Fig. 4:**
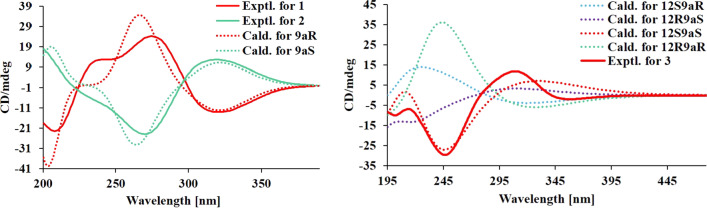
Experimental and calculated ECD curves of alkaloids **1**–**3**

**Fig. 5:**
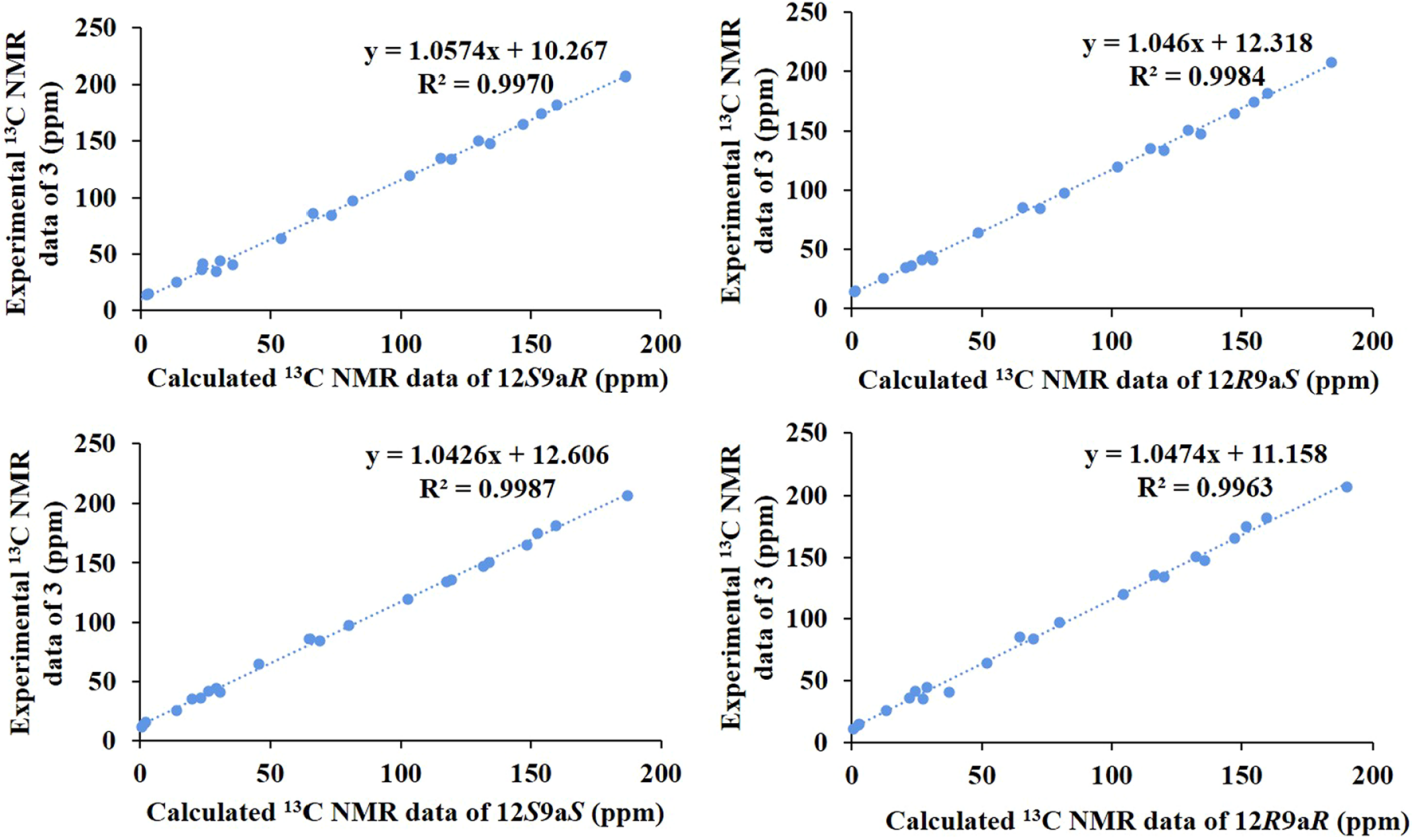
Correlation plots of experimental and ^13^ C NMR spectral data for **3**

The relative configuration of **3** was deduced from the analysis of its ROESY spectra (Fig. 4). The ROESY correlation of H-13 (*δ*_H_ 7.21) with H-1 (*δ*_H_ 2.23, 1.82) and H-2 (*δ*_H_ 1.83, 1.28) indicated that all protons were cofacial, namely 9a***S***,12***S*** or 9a***R***,12***R***. Other relative configurations were same to **1** and **2** by the ROESY correlations and were assigned as *β*-oriented. Hence, the quantum chemical calculations of the NMR data (qcc NMR) of four diastereoisomers need to be performed. The chemical shifts of the four epimers (12***S***9a***R***, 12***R***9a***S***, 12***S***9a***S***, and 12***R***9a***R***) in the NMR spectrum were calculated using the PCM solvent model in methanol at the mPW1PW91/6–31 + G(d,p)//M06-2X / def2-SVP level of theory [10]. The ^13^ C NMR data with the correlation coefficient (R^2^) of 0.9987 of 12***S***9a***S*** were in good agreement with its experimental values (Fig. 5). In addition, ECD calculations of the four model molecules (12***S***9a***R***, 12***R***9a***S***, 12***S***9a***S*** and 12***R***9a***R***) indicated that the calculated Cotton effects of the model molecule 12***S***9a***S*** is the most consistent with the experimental value than the other three corresponding epimers, while the 12***R***9a***S*** is somewhat similar to the experimental spectrum (Fig. 4). Therefore, the absolute configuration of **3** could be determined as 3***S***,9a***S***,12***S***,18***S***,20***S***. Subsequently, **3** was named as stemajapine C.

The other six alkaloids were elucidated as oxymaistemonine (**4**) [12], isooxymaistemonine (**5**) [13], maistemonine (**6**) [12], isomaistemonine (**7**) [13], isostemonamide (**8**) [4], stemonamide (**9**) [4] by comparison NMR data with previous reported.

Compounds (**1**–**8**) were evaluated for their anti-inflammatory activity by measuring LPS-induced nitric oxide (NO) production in RAW264.7 macrophages [14]. The results showed that maistemonine-type alkaloids can inhibite NO production with IC_50_ values ranging from 113.52 ± 3.00 to 13.75 ± 0.24 *µ*M, compounds **1** and **3** displayed certain inhibitory activity with an IC_50_ value of 19.73 ± 3.53 and 13.75 ± 0.24 *µ*M, which approach the activity of positive control Dexamethasone (Table 2), We try to analyze the structure-activity relationship of compounds **1** and **2**. Though sharing the same planar structure, but they had totally different activities on inhibiting NO production, the one with 9a***R*** has stronger activity than isomer with 9a***S***. Compounds **1**–**8** displayed no cytotoxic activity at the concentration of lower than 100 *µ*M. From above data, compounds **1** and **3** could be for anti-inflammatory activity.

**Table 2:** Inhibitory activities of compounds **1**–**8** against LPS-induced NO production in RAW264.7 macrophages

Compounds	IC_50_ (*µ*M) ^a^
**1**	19.73 ± 3.53
**2**	103.23 ± 3.06
**3**	13.75 ± 0.24
**4**	107.62 ± 4.51
**5**	113.52 ± 3.00
**6**	110.16 ± 4.40
**7**	109.15 ± 1.42
**8**	104.78 ± 2.24
Dexamethasone	11.71 ± 0.05

^a^Each value represents as mean ± SD of three independent experiments

The bioassay results disclosed the anti-inflammatory natural constituents stemjapines A and C. Though **1** and **2** were epimers at C-9a, however, both compounds possessed completely different anti-inflammatory activity. This finding would attract pharmacologists to continue research. Careful analysis the structure of stemajapines A and B showed us *Stemona* alkaloids without skeletal methyl. Also, it’s the first representative alkaloid (**1** and **2**) with two kinds of spiro center C-9a. Finally, the inhibiting NO production of **1** may point out a new function direction of *Stemona* alkaloids besides for its traditional antitussive [15–20] and insecticide activities [20, 21].

## Experimental section

### General experimental procedures

UV spectra were recorded on Shimadzu UV-2700 and UV-2401PC spectrometers. Optical rotations were measured with an automatic polarimeter RUDOLPH APVI-6. 1D and 2D NMR spectra were obtained on Bruker AVANCE III-500 and AVANCE III-600 MHz spectrometers with SiMe4 as internal standard. MS data were obtained using Shimadzu UPLC-IT-TOF (Shimadzu Corp, Kyoto, Japan). Column chromatography (CC) was performed on silica gel (200–300 mesh, Qing-dao Haiyang Chemical Co., Ltd., Qingdao, China) or RP-18 silica gel (20–45 *µ*m, Fuji Silysia Chemical Ltd., Japan). Fractions were examined by TLC on silica gel plates (GF254, Qingdao Haiyang Chemical Co., Ltd., Qingdao, China) and spots are visualized with Dragendorff’s spray reagent. MPLC was performed using a Buchi pump system in combination with glass columns filled with RP-18 silica gel (19 × 480, 40 × 480, 45 × 480 and 55 × 480 mm, respectively). HPLC analysis was performed using a Waters 1525 EF pump in combination with a Sunfire C18 pre- or semi-preparative analysis column (4.6 × 150 and 19 × 250 mm, respectively) The HPLC system is combined with a Waters 2998 Photodiode Array Detector and Waters Fraction Collector III [22].

### Plant materials

The roots of *Stemona japonica* were collected in October 2021 in Anhui province, People’s Republic of China and identified by Xiang-hai Cai. The voucher sample (cai20191002) is kept at the State Key Laboratory of Phytochemistry and Plant Resources in West China, Kunming Institute of Botany, Chinese Academy of Sciences.

### Extraction and isolation

Fresh root fragments of *S. japonica* (60 kg) were extracted with 80% methanol at room temperature and the solvent was evaporated under vacuum. The crude extract was suspended in HCl (1%) and separated with water and EtOAc The acid layer was then adjusted to pH 7–8 with 5% ammonia solution and extracted with EtOAc to obtain a crude alkaloid extract (666 g).The crude alkaloids were treated with C_18_ MPLC with MeOH–H_2_O (1:9 to 100:0, *v / v*) to yield three fractions (I–III) based on TLC analysis.

Fr.I (437 g) was chromatographed over a silica gel column eluting with gradient CHCl_3_–Acetone gradient (1:0 to 0:1, *v / v*) to give seven fractions (I-1 ~ 7). Then, Fr.I-7 was subjected to silica gel column eluting with gradient CHCl_3_–MeOH gradient (1:0 to 0:1, *v / v*) to yield Fr.I-7-1 ~ I-7-6. Fr.I-7-2 was subjected to C_18_ MPLC (MeOH–H_2_O, 1:20 to 0:1, *v / v*) to obtain three fractions (I-7-2-1 ~ 3).

Fr.I-7-1 was purified by a Sephadex LH-20 column to obtain five fractions (I-7-1-1 ~ 5), compound **4** (10.4 mg) was crystallized from Fr.I-7-1-1 afterwards. Fr.I-7-1-2 was eluted with MeOH and semi-preparative HPLC (CH_3_CN–H_2_O, 2:3 to 11:9, *v / v*) to give **1** (tR: 31.4 min; 1.8 mg). The subsection I-7-1-4 was purified with a Sephadex LH-20 column and eluted with semi-preparative MeOH and HPLC (CH_3_CN–H_2_O, 2:3 to 11:9, *v / v*) to obtain **8** (tR:20.1 min; 0.8 mg). Fr.I-7-1-5 was eluted with MeOH and semi-preparative HPLC (CH_3_CN–H_2_O, 7:13 to 1:1, *v / v*) to give **6** (tR: 49.4 min; 2.7 mg).

Fr.I-7-2 was purified on a Sephadex LH-20 column, eluted with MeOH, and preparative HPLC (CH_3_CN-H_2_O, 2:5, *v / v*) with isocratic elution to give **7** (tR: 48.4 min; 2.0 mg).

Fr.I-7-3 was subjected to C_18_ MPLC (MeOH-H_2_O, 1:9–4:1, *v / v*) and purified on a Sephadex LH-20 column and eluted with MeOH and semi-preparative HPLC (CH_3_CN–H_2_O, 3:7 to 2:3, *v / v*) to give **9** (tR:31.1 min; 0.5 mg) and **5** (tR: 64.1 min; 1.7 mg).

Fr.I-7-4 was subjected to C_18_ MPLC (MeOH–H_2_O, 1:19 to 7:3, *v / v*) and eluted with semi-preparative HPLC (CH_3_CN–H_2_O, 2:3 to 9:11, *v / v*) to obtain **3** (tR: 29.0 min; 7.3 mg) and **2** (tR: 44.7 min; 3.1 mg).

Stemajapine A (**1**): white amorphous powder; C_17_H_23_NO_3_; [*α*]-7 (*c*, 0.20, MeOH); UV (MeOH) *λ*_max_ (log*ε*): 205 (0.37), 223 (0.52), and 285 (0.06) nm (Fig. S12–20); ^1^ H (500 MHz) and ^13^ C (125 MHz) NMR data (methanol-d4), see Table 1; positive HRESIMS *m / z* 312.1576 [M + Na]^+^ (calcd. for 312.1576).

Stemajapine B (**2**): white amorphous powder; C_17_H_23_NO_3_; [*α*] 46.7 (*c*, 0.06, MeOH); UV (MeOH) *λ*_max_ (log*ε*): 206 (0.48), 226 (0.66), and 276 (0.10) nm (Fig. S21–29); ^1^ H (600 MHz) and ^13^ C (150 MHz) NMR data (methanol-d4), see Table 1; positive HRESIMS *m / z* 290.1756 [M + H]^+^ (calcd. for 290.1756).

Stemajapine C (**3**): white amorphous powder; C_22_H_25_NO_5_; [*α*] 60.20 (*c*, 0.10, MeOH); UV (MeOH) *λ*_max_ (log*ε*): 203 (0.40), 270 (0.26), and 322 (0.05) nm (Fig. S30, S31); ^1^ H (500 MHz) and ^13^ C (125 MHz) NMR data (methanol-d4), see Table 1; positive HRESIMS *m / z* 406.1631 [M + Na]^+^ (calcd. for 406.1631).

### Anti-inflammatory activity assay

The instruments, reagents and methods used in the experiment of NO release inhibition are the same as those described in the literature [14].

## Supplementary Information

Below is the link to the electronic supplementary material.

Supplementary file1 (PDF 4,576 KB)
