# Supplementary material for: Anti-inflammatory maistemonine-class alkaloids of Stemona japonica
Source: Nat Prod Bioprospect. 2023 Mar 13;13(1):8. doi: 10.1007/s13659-023-00372-5 (PMC10011249; doi:10.1007/s13659-023-00372-5)
Supplement: Supplementary file 1 — Supplementary file1 (PDF 4,576 KB) [file 13659_2023_372_MOESM1_ESM.pdf]

Supplementary information

Anti-inflammatory of Maistemonine-class Alkaloids of *Stemona japonica*

Cheng-Yong Tan,[a,b] Mei-Fen Bao,[a] Bao-Bao Shi[c], and Xiang-Hai Cai*[a]

[a] State Key Laboratory of Phytochemistry and Plant Resources in West China, Kunming Institute of Botany, Chinese Academy of Sciences, Kunming 650201, People's Republic of China

[b]University of Chinese Academy of Sciences, Beijing 100049, China

[c]School of Pharmaceutical Sciences, South-Central MinZu University, Wuhan 430074, People's Republic of China

Contents

Quantum chemical calculation 4

1. General computational data and results of **1** and **2**. 4

Figure S1. Correlations between calculated and experimental ^{13}C NMR chemical shifts of **1** and **9a*R***, **2** and **9a*S*** 5

Table S1. Experimental and calculated ^{13}C NMR chemical shifts of **9a*R*** and **9a*S***. 6

Table S2. Important thermodynamic parameters of the M06-2X/Def2SVP optimized conformers of **9a*R*** in the gas phase. 6

Table S3. Conformational analysis of the M06-2X/Def2SVP optimized conformers of **9a*R*** in the gas phase (T=298.15 K). 6

Table S4. Important thermodynamic parameters of the M06-2X/Def2SVP optimized conformers of **9a*S*** in the gas phase. 6

Table S5. Conformational analysis of the M06-2X/Def2SVP optimized conformers of **9a*S*** in the gas phase (T=298.15 K). 6

Figure S2. Experimental ECD spectra of **1** and **2** calculated ECD spectra of **9a*R*** and **9a*S*** in methanol. 7

2. General computational data and results of **3**. 7

Figure S3. Correlations between calculated and experimental ^{13}C NMR chemical shifts of **3**. 7

Table S6. Experimental and calculated ^{13}C NMR chemical shifts of **3**. 9

Table S7. Important thermodynamic parameters of the M06-2X/Def2SVP optimized conformers of **3** in the gas phase. 8

Table S8. Conformational analysis of the M06-2X/Def2SVP optimized conformers of **3** in the gas phase (T=298.15 K). 9

Figure S4. Experimental ECD spectra of 1 and 2 calculated ECD spectra of **9a*R*** and **9a*S*** in methanol. 10

Table S9. Cartesian coordinates for the low-energy optimized conformers of **9a*R*** at M06-2X/Def2SVP level. 10

Table S10. Cartesian coordinates for the low-energy optimized conformers of **9a*S*** at M06-2X/Def2SVP level. 13

Table S11. Cartesian coordinates for the low-energy optimized conformers of **3** at M06-2X/Def2SVP level. 15

Spectroscopic data 37

Figure S5. ^{1}H NMR Spectrum of **1** in CD_3OD 37
Figure S6. ^{13}C NMR Spectrum of **1** in CD_3OD 38
Figure S7. HMBC Spectrum of **1** in CD_3OD 39
Figure S8. HSQC Spectrum of **1** in CD_3OD 40
Figure S9. ROESY Spectrum of **1** in CD_3OD 41
Figure S10. COSY Spectrum of **1** in CD_3OD 42
Figure S11. HRESIMS Spectrum of **1** 43
Figure S12. UV *S*pectrum of **1** 44
Figure S13. Optical Rotation Spectrum of **1** 44
Figure S14. ^{1}H NMR Spectrum of **2** in CD_3OD 45
Figure S15. ^{13}C NMR Spectrum of **2** in CD_3OD 46
Figure S16. HMBC Spectrum of **2** in CD_3OD 47
Figure S17. HSQC Spectrum of **2** in CD_3OD 48
Figure S18. ^{1}H-^{1}H COSY Spectrum of **2** in CD_3OD 49
Figure S19. ROESY Spectrum of **2** in CD_3OD 50
Figure S20. HRESIMS Spectrum of **2** 51
Figure S21. UV Spectrum of **2** 52
Figure S22. Optical Rotation Spectrum of **2** 52
Figure S23. ^{1}H NMR Spectrum of **3** in CD_3OD 53
Figure S24. ^{13}C NMR Spectrum of **3** in CD_3OD 54
Figure S25. HMBC Spectrum of **3** in CD_3OD 55
Figure S26. HSQC Spectrum of **3** in CD_3OD 56
Figure S27. ROESY Spectrum of **3** in CD_3OD 57
Figure S28. COSY Spectrum of **3** in CD_3OD 58
Figure S29. HRESIMS Spectrum of **3** 59
Figure S30. UV Spectrum of **3** 60
Figure S31. Optical Rotation Spectrum of **3** 60

Quantum chemical calculation

The initial conformational analysis of the compound 1-2 were executed by employing Monte Carlo searching algorithm via the MMFF94 molecular mechanics force field, with the aid of the SPARTAN'16 program package, leading to afford a panel of relatively favored conformations in an energy range of 3 kcal/mol above the global minimum. The force field minimum energy conformers thus obtained were subsequently optimized by applying the density functional theory (DFT) with the M06-2X/Def2SVP level in vacuum, implemented in the Gaussian 09 software package. Harmonic vibrational frequencies were also performed to confirm no imaginary frequencies of the finally optimized conformers. These predominant conformers were subjected to theoretical calculation of ECD by utilizing Time-dependent density functional theory (TDDFT) calculations at the M06-2X/Def2SVP level in MeOH using the Polarizable Continuum Model (PCM) solvent model. The energies, oscillator strengths, and rotational strengths of each conformers were carried out with Gaussian 09 software package. The oretical calculations of ECD spectra for each conformer were then approximated by the Gaussian distribution. The final ECD spectrum of the individual conformers was summed up on the basis of Boltzmann-weighed population contribution by the SpecDisv1.71. Gauge Independent Atomic Orbital (GIAO) calculations of their 1H and 13C NMR chemical shifts using density functional theory (DFT) at the mPW1PW91/6-311+G(d,p) level with the PCM model in methanol. The calculated NMR data of these conformers were averaged according to the Boltzmann distribution theory and their relative Gibbs free energy. The 1H and ^{13}C NMR chemical shifts for TMS were also calculated by the same procedures and used as the reference.

1. General computational data and results of 1 and 2.

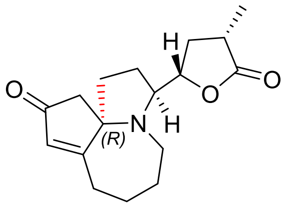

1

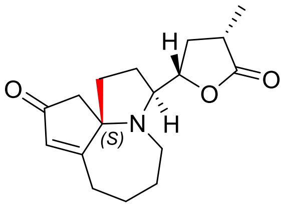

2

Conformation search based on molecular mechanics with MMFF force fields were performed for **9a*R*** and **9a*S***. More than 220000 initial geometries were searched in theory, and 5 conformers were found within 3 kcal/mol from the global minimum. Those conformers were subjected to DFT geometry optimization at the M06-2X/def2-SVP level of theory with Grimme's DFT-D3 dispersion correction. Frequency analysis of all optimized conformations was undertaken at the same level of theory to ensure they were true local minima on the potential energy surface. Finally, 5 predominant conformations are given for subsequent NMR calculations, respectively. Gauge Independent Atomic Orbital (GIAO) calculations of their 1H and ^{13}C NMR chemical shifts using density functional theory (DFT) at the mPW1PW91/6-311+G(d,p) level with the PCM model in methanol. The calculated NMR data of these conformers were averaged according to the Boltzmann distribution theory and their relative Gibbs free energy. The 1H and ^{13}C NMR chemical shifts for TMS were also calculated by the same procedures and used as the reference.

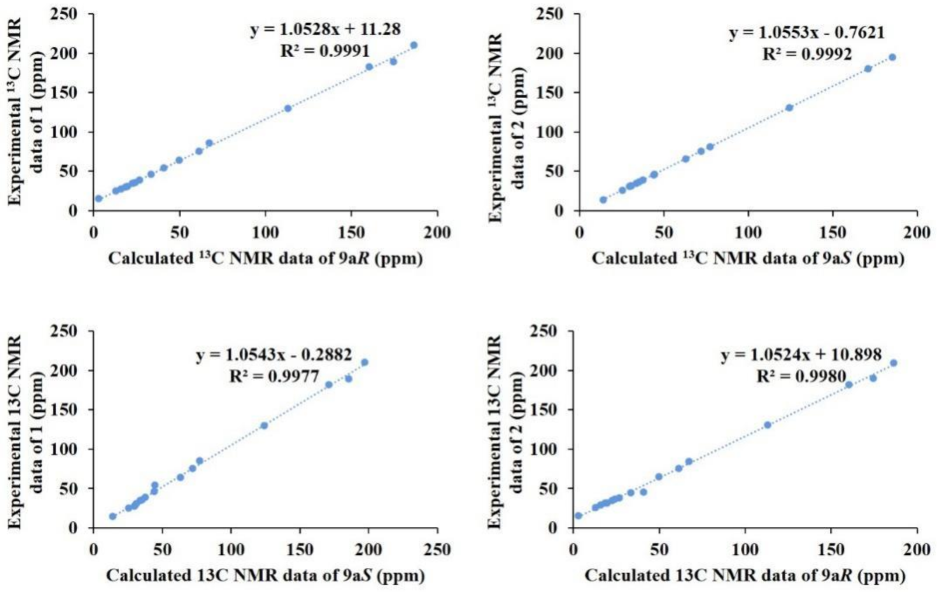

Figure S1. Correlations between calculated and experimental ^{13}C NMR chemical shifts of **1** and **9a*R***, **2** and **9a*S***.

Table S1. Experimental and calculated ^{13}C NMR chemical shifts of **9a*R*** and **9a*S***.

Num.	Exp.(CD_3OD) of 1	**9a*R***	Exp.(CD_3OD) of 2	**9a*S***
1	38.66	26.85	38.18	37.62
2	27.76	15.98	25.95	25.36
3	64.23	49.86	65.29	62.91
5	46.24	33.36	45.35	44.34
6	30.12	18.65	31.71	30.93
7	25.19	12.76	31.36	30.46
8	30.98	19.68	29.38	29.76
9	189.22	174.30	189.69	185.37
**9a**	75.51	61.15	75.35	71.92
10	130.13	112.97	130.37	124.32
11	210.11	186.56	209.37	197.04
12	54.12	40.90	44.94	43.87
13	85.59	67.25	84.13	77.23
14	35.22	22.78	34.89	33.63
15	36.02	24.00	36.21	35.40
16	182.2	160.22	182.15	171.10
17	15.02	2.93	15.14	13.93
R^2	0.9991		0.9992	
MAE	3.41		3.14	
RMSD	7.81		6.80	

Table S2. Important thermodynamic parameters of the M06-2X/Def2SVP optimized conformers of **9a*R*** in the gas phase.

Conformers	E[a] (Hartree)	C[b] (Hartree)	G[c] (kcal/mol)
**1**_1	-940.999026	0.335247	-590273.105969
**1**_2	-941.001183	0.336098	-590273.105969
**1**_3	-940.996137	0.335457	-590271.160697
**1**_4	-940.997002	0.335977	-590271.378442
**1**_5	-940.999561	0.336220	-590272.830493

[a]Electronic energy; [b]Thermal correction to Gibbs free energy ; [c]Gibbs free energy (E + C).

Table S3. Conformational analysis of the M06-2X/Def2SVP optimized conformers of **9a*R*** in the gas phase (T=298.15 K).

Conformers	ΔG (kcal/mol)[a]	Population[b]
**1**_1	0.000000	12.09%
**1**_2	0.000000	71.39%
**1**_3	-1.945272	0.59%
**1**_4	-1.727527	0.83%
**1**_5	-0.275476	15.11%

Table S4. Important thermodynamic parameters of the M06-2X/Def2SVP optimized conformers of **9a*S*** in the gas phase.

Conformers	E[a] (Hartree)	C[b] (Hartree)	G[c] (kcal/mol)
**2**_1	-940.998721	0.335923	-590272.490322
**2**_2	-940.995621	0.335866	-590270.580944
**2**_3	-941.000104	0.335840	-590273.409996
**2**_4	-940.984991	0.336419	-590263.563533

Table S5. Conformational analysis of the M06-2X/Def2SVP optimized conformers of **9a*S*** in the gas phase (T=298.15 K).

Conformers	ΔG (kcal/mol)[a]	Population[b]
**2**_1	0.000000	35.45%
**2**_2	-1.909378	1.23%
**2**_3	0.919674	63.32%
**2**_4	-8.926789	0.00%

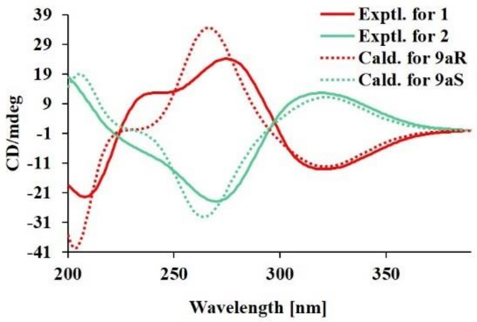

Figure S2. Experimental ECD spectra of **1** and **2** calculated ECD spectra of **9a*R*** and **9a*S*** in methanol.

2. General computational data and results of 3.

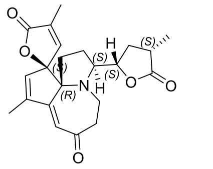

12***S***9a***R***

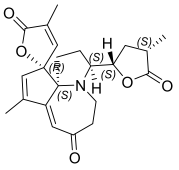

12***R***9a***S***

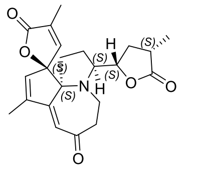

12***S***9a***S***

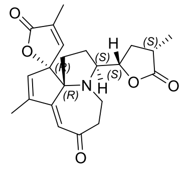

12***R***9a***R***

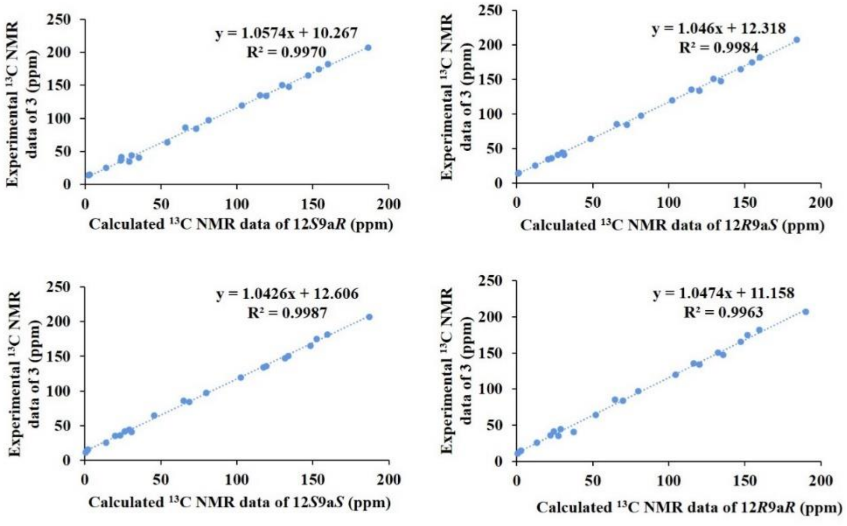

Figure S3. Correlations between calculated and experimental ^{13}C NMR chemical shifts of **3**.

Table S6. Experimental and calculated ^{13}C NMR chemical shifts of **3**.

Num.	Exp.(CD3OD) of **3**	12***S*9a*R***	12***S*9a*R***	12***S*9a*R***	12***S*9a*R***
1	40.7	35.4	28.1	31.0	37.3
2	25.4	13.7	13.1	14.6	13.3
3	64.1	53.9	48.3	46.2	52.0
5	44.1	30.7	30.9	30.0	28.7
6	41.4	23.8	32.5	27.4	24.3
7	206.9	186.3	183.6	186.9	189.9
8	119.7	103.4	103.6	103.9	104.4
9	165.0	147.0	148.7	149.9	147.2
**9a**	85.6	66.1	64.3	64.7	64.6
10	147.4	134.4	135.4	133.0	135.6
11	135.2	115.1	116.9	121.5	116.4
12	97.4	81.4	81.6	79.8	80.0
13	150.5	129.8	130.7	135.9	132.3
14	133.8	119.5	122.0	118.8	119.9
15	174.6	154.2	154.8	152.0	151.6
16	10.8	0.0	0.2	1.9	0.5
17	13.7	2.1	2.4	3.0	2.6
18	84.1	73.1	72.9	69.1	70.0
19	34.7	29.1	21.5	21.0	27.5
20	35.9	23.5	24.1	24.2	22.2
21	181.8	159.8	159.8	159.5	159.6
22	15.0	2.8	2.9	3.6	2.6
R^2		0.997	0.9984	0.9987	0.9963
MAE		11.2	8.9	8.4	13.6
RMSD		14.8	13.9	7.9	21.4

Table S7. Important thermodynamic parameters of the M06-2X/Def2SVP optimized conformers of **3** in the gas phase.

Conformers	E^a (Hartree)	C^b (Hartree)	G^c (kcal/mol)
12***S*9a*R*** _1	-1282.783088	0.387999	-804711.8950
12***S*9a*R*** _2	-1282.788291	0.387297	-804715.6005
12***S*9a*R*** _3	-1282.781401	0.387862	-804710.9225
12***S*9a*R*** _4	-1282.787394	0.388082	-804714.5451
12***S*9a*R*** _5	-1282.783021	0.388809	-804711.3446
12***S*9a*R*** _6	-1282.788323	0.388555	-804714.8314
12***S*9a*R*** _7	-1282.791199	0.385295	-804718.6817
12***R*9a*S*** _1	-1284.238402	0.386163	-805626.2671
12***R*9a*S*** _2	-1284.236769	0.386415	-805625.0842
12***R*9a*S*** _3	-1284.234867	0.385942	-805624.1871
12***R*9a*S*** _4	-1284.234867	0.385943	-805624.1864

12***R*9a*S*** _5	-1284.233068	0.389053	-805621.1063
12***R*9a*S*** _6	-1284.232638	0.386934	-805622.1660
12***R*9a*S*** _7	-1284.231745	0.386545	-805621.8500
12***S*9a*S*** _1	-1284.237427	0.388483	-805624.1991
12***S*9a*S*** _2	-1284.532391	0.390231	-805808.1942
12***S*9a*S*** _3	-1284.235675	0.386396	-805624.4097
12***S*9a*S*** _4	-1284.236627	0.391920	-805621.5404
12***S*9a*S*** _5	-1284.233617	0.388954	-805621.5126
12***S*9a*S*** _6	-1284.232063	0.386240	-805622.2405
12***S*9a*S*** _7	-1284.229751	0.387126	-805620.2338
12***R*9a*R*** _1	-1282.789545	0.387436	-804716.3000
12***R*9a*R*** _2	-1282.780491	0.386574	-804711.1595
12***R*9a*R*** _3	-1282.789270	0.386599	-804716.6529
12***R*9a*R*** _4	-1282.781888	0.385846	-804712.4934
12***R*9a*R*** _5	-1282.789270	0.386607	-804716.6479
12***R*9a*R*** _6	-1282.789545	0.387437	-804716.2993
12***R*9a*R*** _7	-1282.784237	0.387692	-804712.8089
12***R*9a*R*** _8	-1282.789270	0.386610	-804716.6459

[a]Electronic energy; [b]Thermal correction to Gibbs free energy ; [c]Gibbs free energy (E + C).

Table S8. Conformational analysis of the M06-2X/Def2SVP optimized conformers of **3** in the gas phase (T=298.15 K).

Conformers	ΔG (kcal/mol)a	Population[b]
12***S*9a*R*** _1	0.0000	0.04%
12***S*9a*R*** _2	3.7055	9.71%
12***S*9a*R*** _3	-0.9725	0.00%
12***S*9a*R*** _4	2.6501	1.84%
12***S*9a*R*** _5	-0.5504	0.01%
12***S*9a*R*** _6	2.9364	2.17%
12***S*9a*R*** _7	6.7867	86.22%
12***R*9a*S*** _1	0.0000	52.43%
12***R*9a*S*** _2	-1.1829	31.10%
12***R*9a*S*** _3	-2.0800	0.80%
12***R*9a*S*** _4	-2.0807	0.80%
12***R*9a*S*** _5	-5.1608	11.95%
12***R*9a*S*** _6	-4.1011	2.98%
12***R*9a*S*** _7	-4.4171	0.04%
12***S*9a*S*** _1	0.0000	24.42%
12***S*9a*S*** _2	183.9951	57.98%
12***S*9a*S*** _3	0.2106	11.81%
12***S*9a*S*** _4	-2.6587	0.32%
12***S*9a*S*** _5	-2.6865	5.55%

12***S*9a*S*** _6	-1.9586	0.10%
12***S*9a*S*** _7	-3.9653	0.01%
12***R*9a*R*** _1	0.0000	22.61%
12***R*9a*R*** _2	-5.1405	0.00%
12***R*9a*R*** _3	0.3529	0.00%
12***R*9a*R*** _4	-3.8066	0.22%
12***R*9a*R*** _5	0.3479	27.20%
12***R*9a*R*** _6	-0.0007	22.59%
12***R*9a*R*** _7	-3.4911	0.06%
12***R*9a*R*** _8	0.3459	27.33%

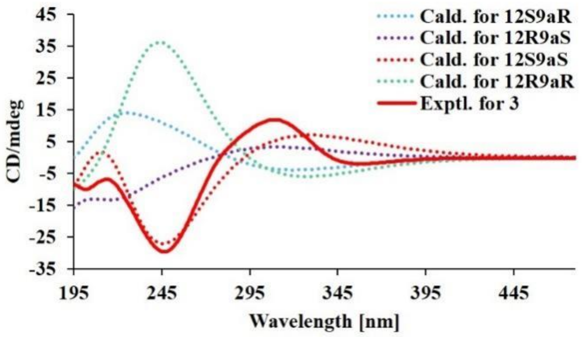

Figure S4. Experimental ECD spectra of **1** and **2** calculated ECD spectra of **9a*R*** and **9a*S*** in methanol.

Table S9. Cartesian coordinates for the low-energy optimized conformers of **9a*R*** at M06-2X/Def2SVP level.

conformer **9a*R***_1							
C	-2.44395	2.547463	0.965063	H	-1.59839	-1.72568	-0.97767
C	-2.54772	1.042662	0.722672	H	-2.477	2.740886	2.045248
C	-1.37308	0.210624	1.251972	H	-3.33779	3.023385	0.542627
N	-0.11658	0.268315	0.511793	H	-3.45809	0.691497	1.226238
C	0.719076	1.463909	0.664386	H	-2.71451	0.850074	-0.3427
C	0.044456	2.761844	1.033636	H	-1.19435	0.459886	2.305989
C	-1.2118	3.211597	0.359712	H	-1.69859	-0.83689	1.27507
C	-0.15668	-0.1191	-0.90424	H	-1.31656	4.298637	0.469908
C	-0.52728	-1.58877	-1.17769	H	-1.16506	3.02191	-0.71819
O	-0.31772	-1.90696	-2.56065	H	-0.86421	-4.34074	-1.1058
C	0.026763	-3.22524	-2.67232	H	-0.09836	-2.794	0.594773
C	0.097734	-3.8544	-1.30491	H	1.331083	-2.36576	-0.35268
C	0.27185	-2.63434	-0.42254	H	1.07754	-5.70894	-1.82499
O	0.214104	-3.79151	-3.7361	H	1.320164	-5.20829	-0.14148
C	1.238235	-4.84525	-1.1709	H	2.196608	-4.3968	-1.45536

C	1.455793	1.607918	-0.68933	H	2.524089	1.829672	-0.5918
C	1.238553	0.273967	-1.38255	H	1.017028	2.398611	-1.31099
C	1.729924	1.244625	1.80848	H	1.296948	0.371621	-2.47124
C	1.806911	2.613014	2.412623	H	2.008898	-0.43535	-1.06235
C	0.688288	3.457965	1.979352	H	2.712685	0.911334	1.463561
O	2.64817	2.989964	3.213339	H	1.359706	0.558634	2.579204
H	-0.86089	0.513726	-1.46118	H	0.454002	4.414257	2.412019

conformer **9a*R***_2							
C	-1.2132	2.040201	2.362214	H	-0.50852	-2.04838	-1.34076
C	-1.8319	1.073998	1.35259	H	-1.47467	1.714382	3.377514
C	-1.27825	-0.35628	1.387752	H	-1.68401	3.021989	2.226713
N	0.057483	-0.57481	0.838338	H	-2.90761	1.020203	1.565991
C	1.204107	-0.1789	1.659729	H	-1.75843	1.498507	0.345668
C	1.023312	0.96006	2.632377	H	-1.32069	-0.73449	2.417616
C	0.29876	2.210087	2.249062	H	-1.97777	-1.00141	0.843179
C	0.288263	-0.17276	-0.56084	H	0.604592	3.027732	2.914421
C	-0.51243	-0.98157	-1.60127	H	0.575971	2.526108	1.237444
O	-1.88094	-0.54218	-1.63872	H	-1.36657	-2.23926	-3.91538
C	-2.36751	-0.66529	-2.90894	H	0.826432	-1.39699	-3.28188
C	-1.27591	-1.1507	-3.82533	H	0.225294	0.271222	-3.20231
C	-0.04094	-0.77962	-3.02946	H	-2.22354	-0.7785	-5.73115
O	-3.52145	-0.4288	-3.22433	H	-0.4516	-0.79186	-5.79302
C	-1.31501	-0.4919	-5.19073	H	-1.31626	0.600851	-5.11085
C	2.314084	0.157534	0.636857	H	3.290702	-0.2741	0.881156
C	1.792186	-0.39269	-0.67849	H	2.455489	1.24067	0.530253
C	1.639082	-1.35334	2.559319	H	2.231762	0.132023	-1.53244
C	2.08466	-0.63547	3.796306	H	2.035846	-1.45919	-0.76642
C	1.579695	0.742093	3.83098	H	2.452368	-1.94898	2.13549
O	2.738008	-1.12335	4.705201	H	0.807307	-2.0119	2.834696
H	0.077264	0.89692	-0.68547	H	1.62454	1.378145	4.696932

conformer **9a*R***_3							
C	-2.62074	2.557889	0.549636	H	-1.21837	-1.95011	-1.29265
C	-2.87049	1.08498	0.22421	H	-2.89273	2.740908	1.597639
C	-1.9343	0.06446	0.892098	H	-3.31446	3.159455	-0.05128
N	-0.52079	0.063764	0.512312	H	-3.89509	0.848697	0.541628
C	0.336356	1.081425	1.115704	H	-2.87824	0.951359	-0.86258
C	-0.24793	2.465796	1.277078	H	-2.02955	0.155516	1.98122
C	-1.20032	3.057627	0.291012	H	-2.3317	-0.93348	0.665972
C	-0.24622	-0.05184	-0.93035	H	-1.19697	4.150696	0.392604
C	-0.21052	-1.52442	-1.38967	H	-0.8918	2.853915	-0.73816
O	0.14469	-1.61168	-2.77676	H	0.17125	-4.25914	-1.61975

C	0.820064	-2.78074	-2.99365	H	0.432887	-2.77615	0.283101
C	0.983947	-3.52743	-1.6946	H	1.743588	-1.89229	-0.51361
C	0.795218	-2.41393	-0.68386	H	2.433026	-5.00307	-2.32102
O	1.199492	-3.15341	-4.0912	H	2.455939	-4.65727	-0.58215
C	2.333443	-4.20901	-1.57324	H	3.156426	-3.50439	-1.73675
C	1.573013	1.079723	0.197907	H	2.294472	0.311087	0.503481
C	1.055452	0.716464	-1.17952	H	2.107792	2.036209	0.179571
C	0.77018	0.722216	2.551967	H	0.844404	1.635323	-1.73977
C	0.872395	2.078454	3.179154	H	1.80481	0.158782	-1.74963
C	0.12524	3.07913	2.409084	H	1.728012	0.197698	2.606287
O	1.43455	2.335821	4.231697	H	0.01215	0.151901	3.099592
H	-0.99687	0.45475	-1.54583	H	-0.13169	4.054301	2.78308

conformer **9a*R***_4							
C	-0.82313	-2.891	0.806413	H	-1.12182	2.613176	0.22197
C	-1.28186	-1.54229	1.359674	H	-1.69714	-3.44394	0.438008
C	-1.86747	-0.57298	0.324615	H	-0.4182	-3.4806	1.638475
N	-0.93343	0.077101	-0.59182	H	-2.06052	-1.74149	2.107701
C	-0.42503	-0.71274	-1.71551	H	-0.46109	-1.07587	1.914704
C	-0.31286	-2.20716	-1.54168	H	-2.65008	-1.08718	-0.24865
C	0.235601	-2.81141	-0.28919	H	-2.41231	0.209521	0.865601
C	0.162481	0.868445	0.000481	H	0.591062	-3.82829	-0.49983
C	-0.31161	2.123304	0.775968	H	1.110842	-2.25351	0.061315
O	-0.81856	1.735713	2.064724	H	1.40004	3.658115	3.092113
C	0.113351	1.961895	3.032035	H	0.27655	4.132178	1.18979
C	1.263009	2.758379	2.482356	H	1.54682	3.2586	0.356758
C	0.760226	3.149031	1.106724	H	2.859868	1.702655	3.483548
O	0.017152	1.557786	4.179314	H	3.353962	2.505017	1.983651
C	2.544948	1.943996	2.462364	H	2.427715	0.995029	1.932004
C	0.961184	-0.1018	-2.02017	H	1.145641	0.075821	-3.08519
C	0.978038	1.202407	-1.24284	H	1.776693	-0.73732	-1.65178
C	-1.35962	-0.56205	-2.93269	H	2.003727	1.503737	-1.00956
C	-1.27478	-1.92555	-3.54733	H	0.506138	2.000548	-1.8297
C	-0.72892	-2.90854	-2.60409	H	-1.03585	0.208383	-3.638
O	-1.65483	-2.22509	-4.6684	H	-2.40396	-0.38539	-2.6507
H	0.762127	0.224885	0.653155	H	-0.74409	-3.97152	-2.76634

conformer **9a*R***_5							
C	-2.13566	3.012474	-0.27311	H	0.214651	-2.51197	-1.97193
C	-2.53093	1.541372	-0.35469	H	-2.27008	3.368331	0.756602
C	-1.69164	0.600828	0.518811	H	-2.83433	3.590776	-0.89055
N	-0.37495	0.227387	0.017126	H	-3.5748	1.461754	-0.02423
C	0.700061	1.223638	0.030501	H	-2.52642	1.21729	-1.40133

C	0.311682	2.684326	0.141186	H	-1.59936	1.015628	1.53076
C	-0.71805	3.309929	-0.74296	H	-2.27	-0.32255	0.652017
C	-0.35626	-0.56561	-1.22144	H	-0.58292	4.399555	-0.74826
C	0.202698	-1.99607	-1.00209	H	-0.58193	2.993602	-1.78168
O	1.553759	-1.99255	-0.52161	H	0.50732	-4.67295	-0.34987
C	1.749323	-3.07693	0.285579	H	-1.47295	-3.26243	-0.40176
C	0.473118	-3.8704	0.395897	H	-0.8338	-2.23888	0.89396
C	-0.55813	-2.82833	0.012623	H	0.305948	-3.6562	2.551977
O	2.819909	-3.36186	0.795787	H	1.016245	-5.18207	2.025043
C	0.250268	-4.43686	1.785141	H	-0.72926	-4.92021	1.856451
C	1.486636	0.98227	-1.2739	H	2.369014	0.353041	-1.11129
C	0.513594	0.244882	-2.17614	H	1.838497	1.901512	-1.75632
C	1.594325	0.998337	1.269586	H	-0.10578	0.966698	-2.72087
C	1.887032	2.396276	1.714263	H	1.032304	-0.36833	-2.91993
C	1.012435	3.368368	1.054786	H	2.522063	0.462568	1.051695
O	2.709463	2.719854	2.557203	H	1.064167	0.492422	2.085183
H	-1.35696	-0.67935	-1.65549	H	0.969675	4.410442	1.317292

Table S10. Cartesian coordinates for the low-energy optimized conformers of **9a*S*** at M06-2X/Def2SVP level.

conformer **9a*S***_1							
C	-1.0134	-2.98704	-1.20675	H	1.769776	1.438886	-1.53429
C	-0.02684	-1.99083	-1.83159	H	-1.88743	-3.07944	-1.86482
C	-0.47182	-0.52076	-1.88593	H	-0.53286	-3.97364	-1.20003
N	-0.53631	0.01401	-0.53834	H	0.929253	-2.06111	-1.29678
C	-1.82038	-0.08629	0.152052	H	0.179836	-2.32157	-2.85751
C	-2.37605	-1.47913	0.232027	H	-1.41468	-0.4223	-2.43432
C	-1.47353	-2.67057	0.222191	H	0.276084	0.016743	-2.47984
C	-0.006	1.35808	-0.29356	H	-2.01397	-3.54193	0.613245
C	1.520294	1.496802	-0.46713	H	-0.61554	-2.51817	0.886602
O	1.95676	2.784733	-0.00909	H	4.276375	1.129284	-0.47824
C	3.217598	2.677268	0.507635	H	2.470632	-0.45314	-0.20236
C	3.682086	1.245899	0.435399	H	1.943776	0.305704	1.304039
C	2.365266	0.506215	0.312645	H	5.423056	1.381369	1.708569
O	3.861997	3.62222	0.931079	H	4.719946	-0.24347	1.612568
C	4.481739	0.824158	1.653286	H	3.933922	1.019734	2.581821
C	-1.38805	0.476419	1.513499	H	-2.22455	0.793486	2.146268
C	-0.48812	1.658268	1.141761	H	-0.80597	-0.25941	2.084976
C	-3.00102	0.738352	-0.40354	H	0.315429	1.76922	1.875943
C	-4.15717	-0.15694	-0.06485	H	-1.0683	2.588849	1.149629
C	-3.71383	-1.53137	0.205429	H	-3.12224	1.713273	0.076768
O	-5.33361	0.169191	-0.07799	H	-2.97055	0.863716	-1.49001

H	-0.48925	2.086481	-0.95994	H	-4.37235	-2.38125	0.229461

conformer **9a*S***_2							
C	-1.42817	-2.29429	1.487297	H	-1.27629	0.849862	-2.29919
C	-2.41827	-1.11992	1.39985	H	-1.92467	-3.11962	2.013886
C	-1.99031	0.009336	0.444655	H	-1.2186	-2.67143	0.478148
N	-0.5597	0.274955	0.429645	H	-3.38401	-1.50776	1.051043
C	0.104693	0.542643	1.703898	H	-2.6088	-0.71523	2.401261
C	0.033757	-0.59352	2.688127	H	-2.56667	0.913412	0.673881
C	-0.09293	-2.00467	2.208076	H	-2.29748	-0.30143	-0.56112
C	-0.10321	1.288379	-0.53227	H	0.002915	-2.69733	3.054227
C	-0.21749	0.862386	-2.00954	H	0.743489	-2.24393	1.54018
O	0.433707	1.816749	-2.85933	H	-0.22711	-0.52608	-4.415
C	0.95299	1.170773	-3.94663	H	-0.21458	-1.31993	-2.1274
C	0.68249	-0.30815	-3.84349	H	1.374398	-0.60725	-1.82108
C	0.429438	-0.46553	-2.35793	H	1.994319	-1.01143	-5.4102
O	1.521463	1.735749	-4.86594	H	1.65063	-2.21748	-4.1568
C	1.841182	-1.15442	-4.33516	H	2.777812	-0.8837	-3.8352
C	1.535026	0.771651	1.191903	H	2.179606	1.296544	1.905934
C	1.340845	1.585085	-0.08617	H	2.029237	-0.17738	0.943281
C	-0.374	1.756055	2.522072	H	2.105178	1.324122	-0.82435
C	-0.13785	1.264491	3.921676	H	1.448061	2.65449	0.132074
C	-0.01284	-0.19987	3.966823	H	0.199299	2.667501	2.332115
O	-0.12347	1.961568	4.923968	H	-1.4435	1.958492	2.410015
H	-0.67492	2.218736	-0.40478	H	-0.08568	-0.78263	4.867548

conformer **9a*S***_3							
C	-0.93243	-3.47843	-0.95698	H	2.185029	0.228039	-0.14245
C	-0.20042	-2.33765	-1.67791	H	-1.94448	-3.56749	-1.37338
C	-0.72639	-0.91802	-1.4161	H	-0.41845	-4.41578	-1.20489
N	-0.41749	-0.54378	-0.04641	H	0.867172	-2.38262	-1.42402
C	-1.4533	-0.82345	0.945115	H	-0.26048	-2.53003	-2.7568
C	-1.92157	-2.25066	0.97692	H	-1.79546	-0.85694	-1.64755
C	-1.01331	-3.36706	0.570745	H	-0.23759	-0.25714	-2.13541
C	0.109853	0.800769	0.225775	H	-1.39997	-4.31474	0.966641
C	1.522716	1.081719	-0.33842	H	-0.01621	-3.23766	1.006474
O	1.481304	1.288468	-1.76005	H	4.065239	2.107118	-0.76795
C	2.441691	2.187011	-2.12638	H	2.604399	2.259128	1.162974
C	3.151256	2.69708	-0.90166	H	1.380301	3.15947	0.247221
C	2.140156	2.370214	0.1787	H	3.894866	4.534062	-0.0341
O	2.682018	2.498279	-3.28064	H	2.57859	4.771689	-1.20182
C	3.471585	4.177406	-0.97848	H	4.198897	4.373726	-1.77353
C	-0.67837	-0.41696	2.203238	H	-1.31734	-0.21033	3.069221

C	0.093082	0.831489	1.768044	H	0.034803	-1.1961	2.505223
C	-2.76529	-0.01602	0.855541	H	1.09151	0.809705	2.217082
C	-3.75393	-1.02455	1.363797	H	-0.41467	1.737457	2.118524
C	-3.21293	-2.3894	1.302673	H	-2.77373	0.878324	1.485104
O	-4.89417	-0.7786	1.724592	H	-3.04301	0.251465	-0.16842
H	-0.59163	1.549333	-0.16723	H	-3.81186	-3.27853	1.386968

conformer **9a*S***_4							
C	-3.33019	-0.62558	-0.00394	H	3.223601	-0.15754	0.751162
C	-2.04706	-1.03214	-0.72276	H	-4.10009	-0.41944	-0.75862
C	-0.75701	-0.83723	0.082702	H	-3.70162	-1.48192	0.573511
N	-0.13407	0.47631	-0.1487	H	-2.13941	-2.09133	-0.99711
C	-0.86607	1.638147	0.414136	H	-1.97372	-0.49438	-1.67766
C	-2.38094	1.670377	0.379083	H	-0.08902	-1.6107	-0.30363
C	-3.20759	0.560332	0.941577	H	-0.90732	-1.0566	1.146111
C	1.234997	0.599408	0.436494	H	-4.2172	0.918602	1.181539
C	2.289011	-0.5235	0.303392	H	-2.78987	0.229173	1.898762
O	1.943724	-1.71302	1.018856	H	4.220724	-2.27673	-0.67748
C	2.441647	-2.79672	0.350003	H	3.236721	-0.29561	-1.6477
C	3.150632	-2.34969	-0.9035	H	1.634196	-1.0352	-1.70553
C	2.557429	-0.97162	-1.11905	H	3.352966	-4.26536	-1.88345
O	2.336742	-3.94542	0.746518	H	3.361288	-2.88047	-2.99052
C	2.912104	-3.28148	-2.0764	H	1.842278	-3.43498	-2.25676
C	-0.32882	1.77896	1.856643	H	-0.15917	2.824837	2.138143
C	0.979933	0.998265	1.88806	H	-1.00529	1.357164	2.608261
C	-0.48957	2.887836	-0.43172	H	0.867451	0.122693	2.537955
C	-1.78209	3.631664	-0.52201	H	1.797217	1.607682	2.288089
C	-2.90997	2.801912	-0.10302	H	0.280248	3.511689	0.032068
O	-1.9151	4.775926	-0.92734	H	-0.18749	2.628802	-1.45386
H	1.696293	1.461294	-0.06776	H	-3.94052	3.095603	-0.19455

Table S11. Cartesian coordinates for the low-energy optimized conformers of **3** at M06-2X/Def2SVP level.

12***S*9a*R***-1							
C	0.686846	3.743908	1.451019	C	3.909132	1.261431	3.369166
C	0.125497	3.402505	0.108932	H	-2.98296	0.72515	-0.4174
C	-0.78042	2.196007	0.221236	H	-0.49727	-1.024	-0.54865
N	-0.07023	0.920261	0.04445	H	-0.45489	4.268436	-0.23373
C	0.862077	0.524187	1.146532	H	0.93392	3.265679	-0.61841
C	1.801264	1.537372	1.841573	H	-1.49087	2.31452	-0.60387
C	1.723513	2.864335	2.015684	H	-1.36657	2.219867	1.148303
C	-1.03879	-0.22267	-0.03712	H	2.463857	3.412779	2.589871

C	-0.11711	-0.04796	2.20513	H	0.333826	-0.80321	2.858759
C	-1.28083	-0.63648	1.415314	H	-0.49989	0.745754	2.860724
C	2.464741	-0.49719	-0.68031	H	-2.2257	-0.24161	1.806092
C	2.315701	-1.65341	-1.3238	H	-1.30824	-1.72744	1.511005
C	1.670896	-2.57252	-0.38026	H	2.891152	0.401258	-1.10276
O	1.457064	-1.93245	0.803266	H	3.798467	-1.14863	1.889594
O	1.351369	-3.72247	-0.61938	H	-4.83738	-0.52473	-1.99027
C	1.935189	-0.58976	0.706653	H	-3.77554	-1.53434	-0.0526
C	3.028426	-0.40922	1.714915	H	-2.47926	-2.22089	-1.04789
C	2.951256	0.774708	2.333022	H	-4.92594	-2.2401	-3.7829
C	-2.35316	-0.06848	-0.8382	H	-4.96601	-3.05302	-2.20769
O	-2.06775	0.262786	-2.2051	H	-3.47156	-3.02433	-3.16614
C	-2.99643	-0.31998	-3.0202	H	3.115867	-1.1995	-3.25013
C	-3.95447	-1.14064	-2.19647	H	3.37788	-2.86458	-2.70929
C	-3.14856	-1.35874	-0.932	H	1.769253	-2.3481	-3.25634
O	-3.04089	-0.15961	-4.22831	H	4.636708	0.48726	3.636277
C	-4.3502	-2.43919	-2.87268	H	4.469986	2.128695	3.006324
C	2.663217	-2.03569	-2.7075	H	3.377924	1.542851	4.284323
O	0.36588	4.761174	2.061208				

12***S*9a*R***-2							
C	3.705629	1.187671	-0.34432	C	3.906864	-2.89888	-2.2393
C	2.465305	1.894145	0.135557	H	-0.14942	2.497968	1.512887
C	1.253731	1.621789	-0.73806	H	-1.28455	-0.12892	0.457849
N	0.493512	0.424679	-0.41361	H	2.680526	2.969066	0.075898
C	1.219784	-0.86341	-0.36957	H	2.298673	1.664607	1.191151
C	2.6002	-0.98363	-1.03922	H	1.556015	1.563761	-1.79251
C	3.637726	-0.13088	-1.02127	H	0.596264	2.497276	-0.71503
C	-0.40715	0.457665	0.760124	H	4.577379	-0.38583	-1.5043
C	1.393375	-1.136	1.144495	H	1.323656	-2.19714	1.407683
C	0.317527	-0.32513	1.851434	H	2.370797	-0.79967	1.513701
C	-0.56948	-1.59976	-2.15778	H	0.783684	0.336223	2.59085
C	-1.79989	-2.0278	-1.88342	H	-0.37592	-0.98373	2.386244
C	-1.70657	-2.76898	-0.62243	H	-0.29513	-0.99633	-3.01125
O	-0.41199	-2.78999	-0.20299	H	1.26128	-3.8701	-2.09207
O	-2.64087	-3.28083	-0.03343	H	-2.22144	3.786943	2.746755
C	0.390812	-2.02145	-1.10009	H	-1.3949	1.596515	3.394796
C	1.461777	-2.8902	-1.6795	H	-2.54573	0.779709	2.3212
C	2.660614	-2.29534	-1.68067	H	-4.70184	3.707859	2.823037
C	-0.96486	1.809239	1.259247	H	-4.05389	2.552178	4.001297
O	-1.76798	2.425659	0.240908	H	-4.70303	1.984232	2.449257
C	-2.79612	3.112785	0.82157	H	-2.92417	-1.23359	-3.51492
C	-2.75096	2.929393	2.315944	H	-3.4959	-2.79503	-2.90773

C	-1.91532	1.670976	2.435203	H	-3.79861	-1.31249	-1.97815
O	-3.59927	3.79062	0.203147	H	3.725517	-3.90774	-2.62577
C	-4.12815	2.781415	2.933524	H	4.29382	-2.29464	-3.06625
C	-3.07079	-1.83158	-2.60967	H	4.679809	-2.97643	-1.46765
O	4.819666	1.683119	-0.16283				

12***S*9a*R***-3							
C	0.638386	3.171848	1.628078	C	3.787547	0.575458	3.516991
C	0.018768	2.851796	0.306779	H	-2.91627	-1.68356	-0.76379
C	-0.88206	1.640436	0.421176	H	-0.5937	-1.57671	-0.44052
N	-0.17691	0.374006	0.150981	H	-0.57919	3.72236	0.008074
C	0.761459	-0.06362	1.24039	H	0.796966	2.735888	-0.45638
C	1.704394	0.923773	1.968504	H	-1.62517	1.797953	-0.36357
C	1.645871	2.247018	2.174198	H	-1.42348	1.629281	1.374802
C	-1.14568	-0.76933	0.049699	H	2.39466	2.764773	2.766578
C	-0.2084	-0.67644	2.287912	H	0.239721	-1.48157	2.881157
C	-1.40589	-1.18972	1.497085	H	-0.55568	0.082531	3.001672
C	2.384932	-0.98916	-0.6158	H	-2.3258	-0.75312	1.901504
C	2.255104	-2.113	-1.31755	H	-1.49178	-2.27901	1.576641
C	1.601352	-3.08141	-0.43093	H	2.818564	-0.06988	-0.98241
O	1.363135	-2.50205	0.778722	H	3.682721	-1.77625	1.94336
O	1.291279	-4.2179	-0.73712	H	-4.26335	-0.63512	-2.89239
C	1.838783	-1.15554	0.758564	H	-1.88801	-1.09013	-2.85853
C	2.921056	-1.02442	1.786193	H	-1.51378	0.54782	-2.31063
C	2.843137	0.135564	2.448081	H	-4.62449	1.582177	-3.94862
C	-2.44563	-0.69048	-0.78764	H	-3.14694	0.86268	-4.6141
O	-3.4118	0.221581	-0.2598	H	-3.04903	2.1514	-3.39639
C	-4.13478	0.750779	-1.29353	H	3.099347	-1.56601	-3.20028
C	-3.62786	0.211369	-2.6076	H	3.353205	-3.25466	-2.73391
C	-2.24075	-0.26792	-2.22816	H	1.756444	-2.71519	-3.29345
O	-5.06301	1.52763	-1.14508	H	4.50166	-0.21587	3.769791
C	-3.60685	1.259733	-3.70359	H	4.364323	1.448113	3.19433
C	2.63634	-2.42803	-2.70925	H	3.243139	0.831241	4.43191
O	0.403979	4.218882	2.22786				

12***S*9a*R***-4							
C	2.933799	1.516686	1.415616	C	4.600272	-2.36599	-0.14031
C	1.488312	1.93307	1.413843	H	-2.98583	0.648286	1.588253
C	0.773637	1.536807	0.13303	H	-1.61129	-0.81164	0.247568
N	0.175721	0.207161	0.082613	H	1.00277	1.572342	2.322235
C	1.065717	-0.93284	0.427802	H	1.4825	3.029595	1.469837
C	2.597267	-0.76954	0.372353	H	1.465864	1.622386	-0.71622

C	3.371388	0.245577	0.788913	H	0.019155	2.29293	-0.09869
C	-1.09199	-0.02778	0.814415	H	4.454562	0.183622	0.722668
C	0.678646	-1.29342	1.88655	H	0.641866	-2.37286	2.069042
C	-0.67944	-0.65691	2.14074	H	1.388598	-0.88822	2.618858
C	0.358291	-1.81944	-1.95055	H	-0.60049	0.083712	2.943927
C	-0.77083	-2.46406	-2.23567	H	-1.40835	-1.40898	2.463037
C	-1.09462	-3.26918	-1.05431	H	0.863345	-1.12362	-2.60514
O	-0.12645	-3.10426	-0.11238	H	2.346327	-3.77891	-1.08259
O	-2.08477	-3.96603	-0.92871	H	-4.25861	2.859948	0.579266
C	0.835596	-2.15784	-0.57943	H	-3.49115	0.983069	-0.73228
C	2.182621	-2.8036	-0.64392	H	-1.93709	1.752214	-1.05847
C	3.149187	-2.01411	-0.16153	H	-2.26036	4.186645	-1.3545
C	-2.16319	1.070938	0.994623	H	-3.66339	4.955157	-0.61153
O	-1.68132	2.204885	1.719938	H	-3.89679	3.663339	-1.80333
C	-2.30676	3.329725	1.258074	H	-1.20308	-1.76297	-4.20483
C	-3.25845	2.971535	0.144887	H	-1.69099	-3.43458	-3.88676
C	-2.71848	1.6274	-0.30101	H	-2.62517	-2.0881	-3.20262
O	-2.11766	4.445546	1.712025	H	4.775181	-3.37019	-0.54165
C	-3.2695	3.998578	-0.97127	H	5.179032	-1.6635	-0.74874
C	-1.61624	-2.43552	-3.44619	H	4.988612	-2.34898	0.883316
O	3.79401	2.211918	1.95919				

12***S*9a*R***-5							
C	-1.90678	3.64519	0.05772	C	0.381164	3.287833	3.910607
C	-1.46171	2.949917	-1.18751	H	-2.50834	-0.96141	-2.04803
C	-1.74758	1.4682	-1.07939	H	0.098973	-1.23319	-0.50557
N	-0.63862	0.705797	-0.48299	H	-2.03242	3.375499	-2.02255
C	-0.40046	0.927223	0.978646	H	-0.40736	3.169788	-1.39063
C	-0.4615	2.332066	1.623635	H	-1.87615	1.144115	-2.11726
C	-1.1132	3.452867	1.282437	H	-2.70133	1.279742	-0.57104
C	-0.88644	-0.77128	-0.59248	H	-1.08066	4.346444	1.898151
C	-1.50001	0.040204	1.618122	H	-1.24452	-0.3236	2.619808
C	-1.70515	-1.12189	0.6521	H	-2.44555	0.590219	1.716614
C	2.181648	0.670869	0.514615	H	-2.77266	-1.23029	0.428879
C	2.861661	-0.45846	0.326473	H	-1.35791	-2.06142	1.095686
C	2.211893	-1.47641	1.158359	H	2.391248	1.613354	0.029341
O	1.149043	-0.92322	1.805859	H	2.015135	1.023855	3.45599
O	2.546242	-2.64408	1.238015	H	-0.61354	-4.03757	-3.48877
C	1.059102	0.461893	1.467771	H	-2.44383	-3.16755	-2.51237
C	1.257118	1.258587	2.720721	H	-1.62901	-3.35283	-0.9693
C	0.415229	2.295197	2.796255	H	1.650102	-4.12963	-2.52334
C	-1.49542	-1.35252	-1.89797	H	0.49301	-4.67625	-1.29849
O	-0.69262	-0.97416	-3.02425	H	1.20037	-3.05241	-1.20904

C	0.064235	-2.02075	-3.45237	H	4.341166	0.140461	-1.09116
C	-0.32686	-3.28684	-2.74407	H	4.871761	-1.09443	0.061055
C	-1.54938	-2.87135	-1.948	H	3.774802	-1.52722	-1.26693
O	0.939275	-1.92615	-4.29797	H	1.069641	3.008944	4.715771
C	0.813421	-3.81332	-1.89076	H	0.673494	4.281974	3.557638
C	4.023434	-0.7493	-0.53809	H	-0.62286	3.348399	4.34322
O	-2.86132	4.41889	0.076851				

12***S*9a*R***-6							
C	3.395309	2.154882	0.105319	C	4.564273	-1.85517	-1.59384
C	1.999571	2.62076	0.426852	H	-0.85845	2.724905	1.487916
C	0.961123	2.099696	-0.55056	H	-1.29036	-0.10651	0.450715
N	0.411191	0.784267	-0.25826	H	2.013507	3.71522	0.340813
C	1.361927	-0.33529	-0.07842	H	1.76698	2.390677	1.469604
C	2.801449	-0.20352	-0.60442	H	1.379693	2.079059	-1.56598
C	3.650659	0.833625	-0.51996	H	0.152258	2.833271	-0.63006
C	-0.60422	0.665078	0.812458	H	4.667006	0.754446	-0.89678
C	1.421373	-0.52119	1.457686	H	1.526647	-1.56708	1.766142
C	0.140416	0.086586	2.011764	H	2.271669	0.009352	1.904974
C	-0.06767	-1.45712	-1.98613	H	0.386569	0.856513	2.751886
C	-1.21873	-2.09937	-1.79842	H	-0.46096	-0.6787	2.514993
C	-1.10768	-2.77232	-0.50128	H	0.167322	-0.83609	-2.83884
O	0.12531	-2.54054	0.026193	H	2.153168	-3.31974	-1.65548
O	-1.98509	-3.42494	0.033506	H	-4.65794	1.835004	1.876757
C	0.849303	-1.65439	-0.82874	H	-2.72391	2.561372	2.772253
C	2.119282	-2.31022	-1.26774	H	-2.27495	0.875958	2.966871
C	3.175714	-1.49314	-1.18099	H	-5.05408	-0.35778	0.824588
C	-1.48828	1.888448	1.164147	H	-4.2425	-0.60837	2.378726
O	-2.23103	2.300094	0.007066	H	-3.34231	-0.75658	0.858908
C	-3.51749	1.859516	0.080415	H	-2.31826	-1.56884	-3.54958
C	-3.80871	1.295499	1.442905	H	-2.6496	-3.19248	-2.92651
C	-2.54934	1.627185	2.221482	H	-3.29721	-1.76678	-2.08839
O	-4.31215	1.923441	-0.84351	H	4.619099	-2.88828	-1.95401
C	-4.12161	-0.18951	1.374615	H	4.909931	-1.20557	-2.40453
C	-2.43424	-2.15974	-2.6353	H	5.25596	-1.76407	-0.74994
O	4.37006	2.85844	0.378119				

12***S*9a*R***-6							
C	1.755899	2.78311	0.698253	C	5.255084	-0.03401	0.059291
C	0.514057	2.822797	-0.1643	H	-1.4665	1.560206	1.712243
C	0.234472	1.594302	-1.01727	H	-1.54862	-0.75214	-0.29699
N	0.193664	0.357556	-0.2794	H	0.61815	3.690282	-0.82759
C	1.431111	-0.30142	0.125363	H	-0.33095	3.030389	0.497787

C	2.714471	0.534489	0.163301	H	0.996145	1.527612	-1.80516
C	2.863422	1.843491	0.412818	H	-0.69869	1.73744	-1.57311
C	-0.99026	-0.14168	0.425209	H	3.842542	2.307912	0.469311
C	1.072389	-0.73044	1.561034	H	1.660148	-1.57653	1.933382
C	-0.41133	-1.06208	1.507457	H	1.217045	0.097831	2.268693
C	1.436508	-1.48856	-2.21107	H	-0.85794	-0.93119	2.497173
C	0.687339	-2.54604	-2.51505	H	-0.55852	-2.10984	1.223609
C	0.608504	-3.35901	-1.29817	H	1.683269	-0.68606	-2.89055
O	1.361046	-2.7844	-0.32104	H	3.977451	-2.34606	-0.97634
O	-0.02348	-4.39269	-1.18072	H	-4.05487	2.078072	2.442489
C	1.861306	-1.53173	-0.78594	H	-2.95967	-0.06091	2.749235
C	3.355553	-1.51957	-0.65941	H	-3.51195	-0.64671	1.169169
C	3.816907	-0.36706	-0.15455	H	-6.36035	1.624942	1.63877
C	-1.98564	0.882335	1.023662	H	-5.81776	0.257492	2.62843
O	-2.59543	1.66833	-0.0127	H	-5.84223	0.130998	0.857542
C	-3.87837	1.981152	0.334204	H	0.202156	-2.18014	-4.56128
C	-4.21876	1.338007	1.650808	H	0.390184	-3.88803	-4.13569
C	-3.17545	0.240883	1.720207	H	-1.06239	-2.9991	-3.63234
O	-4.60841	2.696592	-0.33058	H	5.903714	-0.87924	-0.19511
C	-5.63746	0.804197	1.697405	H	5.555622	0.812608	-0.56636
C	0.019722	-2.92246	-3.77753	H	5.440975	0.220629	1.107845
O	1.926188	3.613435	1.593577				

12***R*9a*S***-1							
C	-2.44729	-2.99589	0.495105	C	-5.26707	0.404694	-0.36867
C	-0.94564	-2.92219	0.701297	H	1.914555	0.938702	-0.16882
C	-0.39336	-1.57015	1.161877	H	0.996625	-1.83131	-1.04171
N	-0.28586	-0.59107	0.100805	H	-0.46247	-3.21439	-0.24721
C	-1.46556	0.005078	-0.47503	H	-0.70601	-3.72143	1.417327
C	-2.84404	-0.53535	-0.12803	H	0.60832	-1.71071	1.585769
C	-3.2446	-1.77264	0.224754	H	-1.01955	-1.1607	1.970501
C	0.791045	-0.75497	-0.86492	H	-4.31613	-1.9543	0.345873
C	-1.23254	-0.11554	-2.00347	H	-1.74675	0.669467	-2.5714
C	0.285221	-0.07949	-2.14827	H	-1.62416	-1.09377	-2.32084
C	-1.22127	1.789952	1.339644	H	0.636824	-0.59718	-3.05004
C	-0.14369	2.577483	1.350398	H	0.63594	0.961347	-2.19175
C	0.170344	2.899235	-0.07148	H	-1.72746	1.335525	2.192064
O	-0.79463	2.35029	-0.8549	H	-3.54193	2.75215	-0.422
O	1.103667	3.498756	-0.51666	H	4.71238	0.79404	-0.21774
C	-1.64959	1.526248	-0.07938	H	3.250544	0.538464	-2.1549
C	-3.11191	1.754836	-0.32025	H	3.182512	-1.23162	-1.92236
C	-3.78758	0.592998	-0.29525	H	6.474088	-0.98764	-0.17004
C	2.097702	-0.12298	-0.40884	H	6.160116	-0.43814	-1.84083

O	2.568865	-0.76826	0.780803	H	5.499936	-1.99131	-1.26475
C	3.917146	-0.87711	0.785866	H	0.310317	2.81527	3.435487
C	4.473331	-0.25037	-0.48727	H	1.72529	2.607162	2.369007
C	3.252733	-0.26036	-1.40309	H	0.867608	4.153435	2.381282
O	4.519686	-1.39468	1.673747	H	-5.77669	1.359848	-0.54462
C	5.727361	-0.9553	-0.97423	H	-5.53099	-0.29477	-1.17549
C	0.728538	3.065858	2.45365	H	-5.64966	-0.02379	0.570197
O	-3.02359	-4.05624	0.595129				

12***R*9a*S***-2							
C	-3.20775	-2.35177	0.739833	C	-4.8347	1.482539	-1.02163
C	-1.86273	-2.55324	1.426013	H	1.729663	-2.41329	1.116685
C	-0.98983	-1.31396	1.606221	H	0.057863	-2.60226	-0.5667
N	-0.47705	-0.76083	0.373247	H	-1.32331	-3.33329	0.865488
C	-1.32986	0.059581	-0.45803	H	-2.0995	-3.00963	2.399655
C	-2.8364	-0.02002	-0.27111	H	-0.14105	-1.56519	2.262855
C	-3.60247	-1.03239	0.180276	H	-1.55647	-0.53221	2.134302
C	0.443439	-1.57418	-0.4125	H	-4.68944	-0.92235	0.1269
C	-0.95258	-0.36803	-1.90192	H	-1.07091	0.448397	-2.62377
C	0.480363	-0.87187	-1.76658	H	-1.61606	-1.1962	-2.19296
C	-0.77098	2.026828	1.115515	H	0.778924	-1.55393	-2.57088
C	0.43429	2.599519	1.155654	H	1.181897	-0.02594	-1.7552
C	0.965511	2.594347	-0.23834	H	-1.45863	1.858879	1.945043
O	0.02238	2.050153	-1.04914	H	-2.50104	3.233051	-1.07071
O	2.031469	2.962518	-0.63182	H	4.309828	-1.14671	1.461608
C	-1.09809	1.612448	-0.29562	H	2.102801	-0.13159	1.790495
C	-2.39997	2.185411	-0.78332	H	2.088122	0.443675	0.10936
C	-3.38545	1.273056	-0.73043	H	5.76733	0.319148	0.009316
C	1.801381	-1.69517	0.280541	H	4.815361	1.271871	1.183378
O	2.738684	-2.22496	-0.65759	H	4.314131	1.209344	-0.52072
C	3.945141	-1.6179	-0.55406	H	0.726974	3.069727	3.232696
C	3.90547	-0.6064	0.586703	H	2.206426	2.609393	2.335188
C	2.407608	-0.3778	0.764921	H	1.496719	4.203953	2.080584
O	4.861162	-1.88242	-1.26726	H	-5.02007	2.499463	-1.38828
C	4.752692	0.622612	0.299359	H	-5.18731	0.762367	-1.77457
C	1.249951	3.149997	2.272473	H	-5.43809	1.325812	-0.11451
O	-4.00682	-3.26008	0.724335				

12***R*9a*S***-3							
C	-1.76817	-3.21589	0.54354	C	-5.16176	-0.37685	-0.27475
C	-0.30404	-2.87038	0.74804	H	1.75808	1.516792	-0.32008
C	-0.00921	-1.43569	1.193855	H	1.436554	-1.44803	-0.97516
N	-0.08084	-0.46651	0.121039	H	0.224992	-3.08106	-0.19785

C	-1.34997	-0.10209	-0.45837	H	0.074555	-3.60562	1.47281
C	-2.60689	-0.86956	-0.07828	H	0.995958	-1.3827	1.629381
C	-2.77576	-2.15536	0.288144	H	-0.70363	-1.13934	1.995903
C	1.008967	-0.43134	-0.84196	H	-3.7958	-2.52415	0.427066
C	-1.11008	-0.22929	-1.98451	H	-1.75737	0.434968	-2.57032
C	0.377351	0.065233	-2.15413	H	-1.32825	-1.27044	-2.26591
C	-1.38096	1.756314	1.293813	H	0.801203	-0.43728	-3.03298
C	-0.47168	2.732235	1.249003	H	0.541458	1.146475	-2.26765
C	-0.27019	3.069915	-0.18936	H	-1.76411	1.238209	2.173603
O	-1.12375	2.313217	-0.92846	H	-3.88042	2.235503	-0.41718
O	0.507343	3.833988	-0.67913	H	5.361569	0.005614	-0.47642
C	-1.79338	1.373818	-0.10272	H	3.793227	1.424827	-1.48321
C	-3.27848	1.332515	-0.30644	H	3.127516	0.006043	-2.30934
C	-3.73759	0.070851	-0.24159	H	5.182811	-2.46881	-0.40996
C	2.14797	0.493044	-0.40461	H	5.036266	-1.8656	-2.0848
O	2.611889	0.121087	0.893805	H	3.588618	-2.40134	-1.19803
C	3.798428	-0.51957	0.852771	H	-0.03039	3.142848	3.309717
C	4.372399	-0.46705	-0.55898	H	1.384728	3.071043	2.227773
C	3.383922	0.419657	-1.32405	H	0.322605	4.487071	2.175951
O	4.271166	-1.05642	1.806782	H	-5.83592	0.46974	-0.45296
C	4.555669	-1.88592	-1.09749	H	-5.31552	-1.12756	-1.06388
C	0.336317	3.3966	2.30826	H	-5.442	-0.84751	0.679908
O	-2.14016	-4.36453	0.635137				

12***R*9a*S***-4							
C	1.76834	3.215888	0.544852	C	5.161511	0.377634	-0.27715
C	0.30403	2.870328	0.747578	H	-1.75819	-1.51678	-0.31929
C	0.009058	1.435712	1.193708	H	-1.43694	1.447929	-0.97489
N	0.080576	0.466292	0.121092	H	-0.22382	3.080209	-0.19917
C	1.34969	0.102326	-0.4587	H	-0.07569	3.605856	1.47145
C	2.606668	0.869948	-0.07899	H	-0.99615	1.382927	1.629159
C	2.775746	2.155541	0.288062	H	0.703375	1.139448	1.995877
C	-1.00937	0.431223	-0.84177	H	3.795861	2.524155	0.426867
C	1.109384	0.229618	-1.98478	H	1.756725	-0.43437	-2.57085
C	-0.37799	-0.06528	-2.15406	H	1.327213	1.270854	-2.26614
C	1.38234	-1.75599	1.293616	H	-0.80218	0.437074	-3.03285
C	0.473328	-2.7322	1.24962	H	-0.54186	-1.14657	-2.26747
C	0.270545	-3.06985	-0.18856	H	1.766257	-1.23783	2.173035
O	1.123316	-2.31297	-0.92842	H	3.880402	-2.23492	-0.41933
O	-0.50736	-3.83396	-0.67766	H	-5.36207	-0.0063	-0.47675
C	1.793486	-1.37347	-0.10331	H	-3.79325	-1.42569	-1.48262
C	3.278463	-1.33201	-0.308	H	-3.12778	-0.00713	-2.30928
C	3.737419	-0.07029	-0.24322	H	-5.18351	2.468187	-0.41097

C	-2.14831	-0.49314	-0.40422	H	-5.03663	1.864476	-2.0856
O	-2.61244	-0.12073	0.893964	H	-3.58917	2.400581	-1.19873
C	-3.79921	0.519498	0.852584	H	0.03383	-3.14265	3.310748
C	-4.37293	0.466422	-0.55923	H	-1.38208	-3.07352	2.229608
C	-3.38418	-0.42037	-1.32384	H	-0.31777	-4.48782	2.177704
O	-4.27231	1.056358	1.806405	H	5.835654	-0.46878	-0.4563
C	-4.55621	1.885132	-1.09821	H	5.31462	1.128793	-1.06597
C	-0.3331	-3.39731	2.309603	H	5.442379	0.847781	0.677576
O	2.1406	4.364279	0.638592				

12***R*9a*S***-5							
C	3.716734	-1.87711	-0.49833	C	4.193963	2.552461	-0.63131
C	2.427808	-2.6461	-0.29538	H	-1.97296	-2.2749	1.925849
C	1.176711	-1.95158	-0.84904	H	0.308126	-2.5558	1.417057
N	0.564873	-0.97746	0.035318	H	2.306719	-2.83421	0.78552
C	1.317686	0.220544	0.403146	H	2.587571	-3.628	-0.76263
C	2.696106	0.456371	-0.2157	H	0.424733	-2.72135	-1.0668
C	3.688982	-0.39909	-0.53692	H	1.408719	-1.47344	-1.81353
C	-0.10122	-1.54773	1.206683	H	4.640886	0.01924	-0.87369
C	1.477083	0.113957	1.942454	H	1.610607	1.094828	2.415444
C	0.210182	-0.60705	2.383537	H	2.368266	-0.49908	2.146525
C	-0.27367	1.502951	-1.21279	H	0.343473	-1.16278	3.320995
C	-1.4767	2.027903	-0.96679	H	-0.60618	0.112442	2.51323
C	-1.52452	2.351781	0.493857	H	0.125129	1.160696	-2.16852
O	-0.32335	1.992631	1.042069	H	1.517008	3.608897	-0.17538
O	-2.39742	2.855133	1.125269	H	-4.14836	-2.53682	0.389971
C	0.572364	1.552732	0.035415	H	-1.96099	-3.55741	-0.17915
C	1.699781	2.533261	-0.1468	H	-1.51856	-2.07548	-1.09511
C	2.876439	1.917	-0.32887	H	-5.15234	-1.49906	-1.65895
C	-1.60214	-1.74797	1.026918	H	-4.32178	-3.02857	-2.06242
O	-2.27082	-0.48238	0.963458	H	-3.55369	-1.462	-2.43308
C	-3.39455	-0.57161	0.221792	H	-2.46641	1.952959	-2.87138
C	-3.52231	-1.9845	-0.33394	H	-3.55309	1.945373	-1.4385
C	-2.07904	-2.46698	-0.23238	H	-2.74429	3.441514	-1.92421
O	-4.13371	0.347695	0.04686	H	4.10961	3.645954	-0.62749
C	-4.17686	-2.00078	-1.70394	H	4.951126	2.252771	0.108275
C	-2.62422	2.349438	-1.86045	H	4.561192	2.23666	-1.6196
O	4.764001	-2.46153	-0.66598				

12***R*9a*S***-6							
C	-1.16902	-3.19823	1.318711	C	-4.65474	-0.44832	2.203882
C	0.270797	-2.74234	1.325015	H	1.806707	-0.42932	-2.86523
C	0.492534	-1.23966	1.234516	H	0.551817	-2.13854	-1.31504

N	-0.03242	-0.59429	0.042549	H	0.792867	-3.25919	0.513
C	-1.49557	-0.35839	0.008642	H	0.709413	-3.10088	2.264478
C	-2.38285	-1.03208	1.069088	H	1.565687	-1.04127	1.307083
C	-2.26444	-2.25061	1.617224	H	0.088924	-0.7491	2.130352
C	0.457541	-1.04702	-1.27714	H	-3.00104	-2.6464	2.308473
C	-1.91387	-0.89351	-1.37892	H	-2.79365	-0.40073	-1.80543
C	-0.68126	-0.67857	-2.22856	H	-2.1324	-1.96954	-1.34459
C	-0.88713	2.041897	0.896128	H	-0.69167	-1.31467	-3.11983
C	-0.49464	3.055113	0.126026	H	-0.61284	0.362398	-2.56271
C	-1.23595	2.931489	-1.13217	H	-0.51269	1.837531	1.887874
O	-2.07625	1.863361	-1.05626	H	-3.81714	1.990432	1.037754
O	-1.14475	3.680848	-2.08745	H	4.455007	0.221319	-2.27453
C	-1.87208	1.184728	0.183499	H	3.36857	-1.88599	-1.82718
C	-3.17917	1.125146	0.915235	H	2.922641	-1.39477	-0.20155
C	-3.44325	-0.09094	1.409641	H	5.812027	0.847495	-0.29386
C	1.776814	-0.40337	-1.76763	H	5.702268	-0.92002	-0.37949
O	1.843611	0.978921	-1.38233	H	4.715359	-0.01413	0.786054
C	3.121939	1.298481	-1.03089	H	1.002541	3.986612	1.329417
C	4.026711	0.119412	-1.27073	H	1.25327	4.13792	-0.41582
C	3.043937	-1.03197	-1.22379	H	0.000976	5.103561	0.387907
O	3.464188	2.394006	-0.61738	H	-5.33878	0.402279	2.295991
C	5.124187	-0.0025	-0.23049	H	-5.20548	-1.26359	1.723349
C	0.492802	4.126003	0.370696	H	-4.37802	-0.75995	3.216273
O	-1.45205	-4.38907	1.180151				

12***R*9a*S***-7							
C	-2.7552	-2.52542	-1.15679	C	-4.83266	1.486347	-1.17288
C	-1.67655	-2.96592	-0.20104	H	1.77782	0.73639	-0.17351
C	-1.14721	-1.91192	0.761023	H	0.985938	-2.20286	-0.56146
N	-0.44417	-0.78615	0.161024	H	-0.86518	-3.40149	-0.79344
C	-1.25542	0.243956	-0.51958	H	-2.10666	-3.78028	0.395767
C	-2.74749	-0.03689	-0.78531	H	-0.45144	-2.40836	1.450453
C	-3.37513	-1.18758	-1.0672	H	-1.95611	-1.54791	1.407213
C	0.739139	-1.13787	-0.64752	H	-4.43676	-1.20919	-1.29586
C	-0.55559	0.432276	-1.89778	H	0.096753	1.310455	-1.92686
C	0.315528	-0.8	-2.06757	H	-1.24999	0.524005	-2.74106
C	-1.24043	1.491873	1.788338	H	-0.2804	-1.61541	-2.49387
C	-0.1982	2.17811	2.252494	H	1.150802	-0.61649	-2.74894
C	0.423145	2.817381	1.090494	H	-1.90281	0.888403	2.391393
O	-0.30366	2.533207	-0.02437	H	-2.94983	3.193264	0.078772
O	1.431945	3.498474	1.1097	H	4.474253	0.823201	0.073722
C	-1.33736	1.607077	0.306872	H	3.32256	-0.0763	-1.86695
C	-2.66019	2.168323	-0.11032	H	3.306052	-1.71106	-1.17722

C	-3.44202	1.246668	-0.68735	H	6.367433	-0.69888	0.587965
C	1.974721	-0.34056	-0.16379	H	6.027533	-0.77152	-1.15069
O	2.298442	-0.71169	1.187625	H	5.495105	-2.08063	-0.07579
C	3.650356	-0.63963	1.367511	H	-0.26989	1.74876	4.342107
C	4.315819	-0.26141	0.069915	H	1.359107	1.949059	3.679584
C	3.245281	-0.64263	-0.93417	H	0.320946	3.367161	3.935213
O	4.208561	-0.8433	2.432618	H	-5.12214	2.536335	-1.05595
C	5.622949	-0.9956	-0.15858	H	-4.91947	1.237203	-2.23555
C	0.329963	2.317794	3.624534	H	-5.55109	0.881886	-0.60987
O	-3.20712	-3.31066	-1.99305				

12***R*9a*S***-8							
C	2.980945	-0.50322	2.337331	C	3.079961	-3.82284	-0.74492
C	1.732728	-0.09921	3.083143	H	-1.09857	2.462272	1.490298
C	0.406212	-0.31936	2.361634	H	1.244706	1.966932	1.60907
N	0.305342	0.135723	0.976314	H	1.839671	0.956403	3.357279
C	0.888376	-0.75622	-0.05161	H	1.721823	-0.66429	4.023292
C	2.03657	-1.69963	0.357354	H	-0.37297	0.195863	2.938683
C	2.93747	-1.59374	1.343313	H	0.135854	-1.37908	2.432596
C	0.706081	1.538136	0.756946	H	3.734527	-2.31888	1.472925
C	1.434107	0.224957	-1.12624	H	0.707879	0.433846	-1.91602
C	1.708231	1.518009	-0.38999	H	2.343552	-0.12734	-1.62748
C	-1.22599	-2.31947	0.158209	H	2.729112	1.488845	0.01115
C	-2.42105	-2.02551	-0.34902	H	1.641489	2.385221	-1.05374
C	-2.17096	-1.30942	-1.60231	H	-1.06257	-2.85866	1.079355
O	-0.83222	-1.26034	-1.83235	H	0.47662	-3.65422	-1.86858
O	-3.02753	-0.83816	-2.32736	H	-2.95623	3.596815	-0.09004
C	-0.13448	-1.79356	-0.70657	H	-2.13927	1.33231	-0.38204
C	0.776583	-2.89596	-1.15779	H	-0.88003	1.879284	-1.50611
C	1.967911	-2.84884	-0.54511	H	-3.13954	4.628376	-2.3396
C	-0.52106	2.456111	0.555054	H	-3.36388	2.875991	-2.49277
O	-0.10348	3.80856	0.320172	H	-1.82371	3.628537	-2.95566
C	-1.04378	4.440477	-0.44696	H	-3.74592	-2.8644	1.099085
C	-2.14037	3.472796	-0.81145	H	-4.33215	-1.38539	0.323335
C	-1.44132	2.143233	-0.60546	H	-4.33586	-2.92083	-0.5689
O	-0.99229	5.620388	-0.75103	H	2.834363	-4.55653	-1.5203
C	-2.6441	3.657767	-2.22998	H	3.993874	-3.30673	-1.05675
C	-3.7796	-2.3147	0.152907	H	3.285354	-4.37443	0.178099
O	4.079572	-0.03591	2.638617				

12***R*9a*S***-9							
C	-1.72663	-3.02734	-1.01576	C	-5.05644	0.019918	-0.814
C	-0.50611	-3.06694	-0.13242	H	1.489646	1.611004	-0.40503

C	-0.30618	-1.88441	0.804724	H	1.697568	-1.43999	-0.65868
N	-0.074	-0.59426	0.170423	H	0.366841	-3.20801	-0.77837
C	-1.23176	0.089044	-0.44016	H	-0.59734	-3.97212	0.481199
C	-2.55254	-0.68891	-0.60344	H	0.562324	-2.10406	1.439832
C	-2.76442	-1.98776	-0.85983	H	-1.14542	-1.81162	1.508102
C	1.104764	-0.52225	-0.71246	H	-3.76658	-2.3768	-1.01429
C	-0.73639	0.484516	-1.8615	H	-0.44079	1.535727	-1.9319
C	0.500705	-0.36435	-2.09944	H	-1.47267	0.308853	-2.65453
C	-1.4758	1.300487	1.874034	H	0.204633	-1.34056	-2.50096
C	-0.70929	2.315066	2.268476	H	1.170913	0.099166	-2.82828
C	-0.43974	3.116502	1.072471	H	-1.83831	0.512025	2.516977
O	-1.09238	2.574024	0.008451	H	-3.78727	2.284597	0.319458
O	0.249853	4.118867	1.030313	H	5.264155	0.68697	-0.12565
C	-1.71536	1.353264	0.405442	H	3.608924	1.798312	-1.17489
C	-3.17797	1.419401	0.095006	H	3.294125	0.325138	-2.07678
C	-3.63618	0.278528	-0.43598	H	5.474633	-1.76223	0.075123
C	2.023804	0.661606	-0.30092	H	5.114018	-1.35745	-1.6109
O	2.409969	0.523856	1.0761	H	3.851169	-2.01593	-0.55341
C	3.692529	0.085214	1.176881	H	-0.47398	1.917771	4.352338
C	4.358609	0.071874	-0.16949	H	0.928302	2.66767	3.575334
C	3.334217	0.740583	-1.0662	H	-0.5202	3.634573	3.923155
O	4.214029	-0.25976	2.225259	H	-5.67985	0.907285	-0.65944
C	4.713199	-1.34378	-0.59233	H	-5.13123	-0.25502	-1.8712
C	-0.16586	2.650846	3.599868	H	-5.47901	-0.78897	-0.2093
O	-1.92922	-3.92017	-1.84144				

12***S*9a*S***-1							
C	-2.72286	-2.5734	0.705073	C	-5.0727	0.986895	-0.89942
C	-1.50141	-2.43164	1.607362	H	1.819882	-2.10451	1.146003
C	-0.83251	-1.06043	1.638294	H	0.167314	-2.45702	-0.53284
N	-0.4086	-0.58892	0.334491	H	-0.79988	-3.23156	1.324975
C	-1.37883	0.059682	-0.51683	H	-1.85438	-2.69674	2.618105
C	-2.85288	-0.22901	-0.27527	H	0.037481	-1.09302	2.314169
C	-3.41708	-1.35522	0.193119	H	-1.50741	-0.30448	2.059922
C	0.520273	-1.40997	-0.42999	H	-4.50699	-1.45061	0.17812
C	-0.97629	-0.38217	-1.94608	H	-1.18311	0.387082	-2.70201
C	0.49634	-0.76786	-1.81379	H	-1.56519	-1.27259	-2.21012
C	-0.1986	2.317648	-1.01983	H	0.837073	-1.4564	-2.59564
C	0.658803	2.759914	-0.09502	H	1.141524	0.122645	-1.8362
C	0.081536	2.411967	1.238591	H	-0.09234	2.391118	-2.10246
O	-1.15358	1.896879	1.036814	H	-3.03549	3.052539	-0.98423
O	0.576005	2.545516	2.318587	H	4.317061	-0.92275	1.470707
C	-1.35611	1.632636	-0.3535	H	2.161105	0.260019	1.59962

C	-2.7533	2.024776	-0.75152	H	2.234298	0.638473	-0.13378
C	-3.59591	0.978879	-0.68366	H	5.94965	0.369115	0.075133
C	1.884326	-1.45942	0.251298	H	4.945716	1.462013	1.070464
O	2.818455	-2.06259	-0.64817	H	4.611421	1.266296	-0.67182
C	4.040048	-1.49064	-0.53923	H	2.311766	3.531705	-1.2358
C	3.995413	-0.41676	0.542531	H	1.8952	4.469983	0.228914
C	2.502094	-0.11296	0.62464	H	2.72336	2.922507	0.399418
O	4.967069	-1.81393	-1.21394	H	-5.42188	1.975813	-1.22025
C	4.930165	0.741495	0.241283	H	-5.36003	0.242909	-1.65704
C	1.969257	3.458309	-0.19625	H	-5.59395	0.720876	0.032675
O	-3.19429	-3.66553	0.493155				

12***S*9a*S***-2							
C	-3.47217	-1.58066	0.701304	C	-4.01785	2.709248	-0.7311
C	-2.36705	-1.95457	1.679912	H	1.389339	-3.20521	-1.16731
C	-1.11843	-1.07033	1.673016	H	-0.63496	-2.92727	0.034583
N	-0.5534	-0.84151	0.352451	H	-2.14196	-3.01731	1.508087
C	-1.1059	0.238311	-0.43845	H	-2.83299	-1.91303	2.679589
C	-2.56443	0.613561	-0.18687	H	-0.35749	-1.52949	2.324395
C	-3.57274	-0.16944	0.230971	H	-1.33529	-0.08854	2.111149
C	-0.25756	-2.01736	-0.46018	H	-4.59532	0.220053	0.21245
C	-0.97644	-0.24847	-1.90748	H	-0.01266	0.06221	-2.32412
C	-1.00049	-1.77076	-1.77977	H	-1.78119	0.147072	-2.53968
C	0.93726	1.859606	-0.86203	H	-2.03547	-2.13304	-1.68687
C	1.873811	2.095319	0.061934	H	-0.53546	-2.27462	-2.63818
C	1.225385	1.914242	1.393285	H	1.0774	1.891628	-1.94212
O	-0.10235	1.728755	1.194584	H	-1.27206	3.6629	-0.73938
O	1.733155	1.909568	2.47612	H	4.022088	-2.5063	-0.23165
C	-0.39389	1.619504	-0.20496	H	2.108791	-3.15591	1.162031
C	-1.4797	2.606359	-0.56272	H	1.61534	-1.43841	1.315074
C	-2.69799	2.040795	-0.53244	H	5.090433	-0.4023	0.67115
C	1.24254	-2.24538	-0.64099	H	4.610952	-1.52621	1.972042
O	1.79708	-1.21579	-1.46919	H	3.617961	-0.08943	1.634121
C	3.026995	-0.84869	-1.05459	H	3.630047	2.490668	-1.10061
C	3.41722	-1.67179	0.166611	H	3.416741	3.527899	0.353673
C	2.064397	-2.18841	0.645862	H	3.946921	1.850213	0.52793
O	3.667433	-0.00925	-1.61181	H	-3.89163	3.766451	-0.9944
C	4.234421	-0.87572	1.171407	H	-4.59192	2.209951	-1.52588
C	3.297834	2.514035	-0.05752	H	-4.61745	2.650693	0.18998
O	-4.31879	-2.38811	0.397243				

12***S*9a*S***-3							
C	-1.00266	-3.17448	0.46257	C	-5.09531	-1.35908	-0.45582

C	0.032199	-2.45968	1.320487	H	1.915318	1.296647	0.042221
C	-0.12401	-0.94766	1.446056	H	1.203686	-1.48784	-0.92873
N	-0.17826	-0.26799	0.160062	H	1.027895	-2.73755	0.943948
C	-1.44899	-0.19789	-0.52228	H	-0.04186	-2.92289	2.319173
C	-2.50714	-1.23706	-0.18366	H	0.711916	-0.54606	2.033381
C	-2.3167	-2.51244	0.191255	H	-1.04277	-0.68959	1.987572
C	0.931187	-0.42201	-0.76883	H	-3.17759	-3.18358	0.268418
C	-1.0797	-0.29376	-2.02215	H	-1.75933	0.27993	-2.66623
C	0.376143	0.159841	-2.06783	H	-1.14222	-1.34844	-2.32713
C	-1.67648	2.352407	-0.88092	H	0.909474	-0.2021	-2.95624
C	-1.03534	3.120117	0.004929	H	0.451858	1.258005	-2.03891
C	-1.21445	2.487568	1.347411	H	-1.7601	2.519268	-1.95514
O	-2.02138	1.410284	1.194374	H	-4.46949	1.47442	-0.51553
O	-0.77164	2.840981	2.398072	H	4.729965	1.396109	-0.10925
C	-2.22863	1.138786	-0.19231	H	3.220266	1.182442	-2.02636
C	-3.66332	0.745512	-0.42168	H	3.304351	-0.59821	-1.93791
C	-3.81811	-0.59013	-0.38374	H	6.630889	-0.22666	-0.28429
C	2.191795	0.284196	-0.3039	H	6.211241	0.444395	-1.88611
O	2.761239	-0.42703	0.802038	H	5.701052	-1.20489	-1.43829
C	4.113611	-0.42264	0.754989	H	-0.21271	4.710743	-1.18684
C	4.566984	0.364458	-0.46945	H	-0.70369	5.176196	0.470688
C	3.315966	0.330214	-1.34184	H	0.765677	4.233311	0.236003
O	4.791565	-0.96917	1.566636	H	-5.95092	-0.69455	-0.62695
C	5.854452	-0.18406	-1.05928	H	-5.05561	-2.10636	-1.26217
C	-0.25686	4.380127	-0.1421	H	-5.26253	-1.90444	0.485425
O	-0.81539	-4.30712	0.084797				

12***S*9a*S***-4							
C	-3.32892	-1.35047	0.815555	C	-3.72541	2.937227	-0.70613
C	-2.23935	-1.70386	1.816788	H	0.813439	-3.49587	-1.45578
C	-0.91927	-0.94349	1.656672	H	-1.01485	-2.85401	-0.02229
N	-0.44465	-0.82822	0.281333	H	-2.12004	-2.79619	1.784523
C	-0.94299	0.314571	-0.4706	H	-2.65877	-1.47645	2.811984
C	-2.3693	0.774771	-0.1677	H	-0.15411	-1.42948	2.282286
C	-3.39507	0.054057	0.313393	H	-1.0149	0.075612	2.049512
C	-0.46577	-2.04938	-0.53893	H	-4.39777	0.492588	0.332354
C	-0.90182	-0.16334	-1.94255	H	0.109472	-0.03486	-2.34486
C	-1.20257	-1.65213	-1.8252	H	-1.6128	0.384563	-2.57334
C	1.168551	1.809842	-0.99333	H	-2.28235	-1.82019	-1.70085
C	2.131123	2.124852	-0.12226	H	-0.86617	-2.22287	-2.70127
C	1.515905	2.119983	1.23572	H	1.281699	1.732215	-2.0738
O	0.19752	1.815659	1.099975	H	-0.92647	3.727473	-0.84264
O	2.030266	2.327999	2.292941	H	3.858929	-2.3629	0.545216

C	-0.14706	1.651251	-0.28248	H	2.164147	-3.96701	0.371626
C	-1.19171	2.689729	-0.63397	H	1.12264	-2.92824	1.3544
C	-2.43833	2.19904	-0.54196	H	3.486337	-0.08182	1.523997
C	0.929856	-2.61319	-0.80886	H	2.867594	-1.35512	2.602297
O	1.705539	-1.66738	-1.54297	H	1.736462	-0.41386	1.59189
C	2.771181	-1.21315	-0.85472	H	3.821368	2.499598	-1.38146
C	2.861681	-1.90143	0.499884	H	3.815561	3.393335	0.180605
C	1.750151	-2.9542	0.453763	H	4.194476	1.657809	0.131951
O	3.50708	-0.38271	-1.29637	H	-3.55049	3.979862	-0.99818
C	2.732704	-0.87462	1.623428	H	-4.35726	2.455246	-1.46707
C	3.57104	2.443585	-0.31624	H	-4.29084	2.932383	0.238164
O	-4.17797	-2.1551	0.512635				

12***S*9a*S***-5							
C	-1.80492	2.4526	2.783495	C	-0.84085	4.987276	-0.80296
C	-1.51427	0.992662	2.967304	H	-1.26917	-3.2199	0.531055
C	-0.2546	0.508526	2.259515	H	-1.93251	-1.21074	1.503468
N	-0.41992	0.122316	0.858858	H	-2.41303	0.432001	2.696688
C	-0.80187	1.204513	-0.07602	H	-1.36078	0.843646	4.044334
C	-0.97016	2.666254	0.399498	H	0.119005	-0.35915	2.815208
C	-1.38819	3.178305	1.566836	H	0.549894	1.250883	2.340894
C	-1.26134	-1.07728	0.647186	H	-1.45992	4.254415	1.704269
C	-2.17866	0.752489	-0.60925	H	-2.40626	1.117835	-1.61646
C	-2.13524	-0.76024	-0.5663	H	-2.98925	1.092226	0.050027
C	0.326884	0.477093	-2.38615	H	-3.14049	-1.18247	-0.46098
C	1.516082	-0.11281	-2.50159	H	-1.72077	-1.14761	-1.50112
C	2.328208	0.353785	-1.37335	H	-0.49391	0.334265	-3.07324
O	1.62675	1.285467	-0.67753	H	0.573725	3.247949	-2.51286
O	3.460154	-0.01279	-1.1151	H	0.828741	-4.79977	0.945466
C	0.297411	1.391997	-1.22014	H	0.235333	-2.98716	2.451632
C	0.121409	2.822255	-1.62716	H	1.277788	-1.85605	1.559707
C	-0.56134	3.523439	-0.71514	H	3.289379	-4.7497	0.612216
C	-0.51265	-2.42368	0.475879	H	2.915783	-4.08834	2.21452
O	0.119737	-2.56175	-0.80115	H	3.334412	-3.00579	0.870417
C	1.127468	-3.48091	-0.68892	H	1.208017	-1.45866	-4.13042
C	1.333072	-3.8445	0.759873	H	2.744503	-0.57604	-4.16807
C	0.602341	-2.7129	1.45852	H	2.51962	-1.91531	-3.03454
O	1.724271	-3.95336	-1.64188	H	-0.49713	5.404027	-1.75599
C	2.799377	-3.92366	1.138677	H	-1.91614	5.180214	-0.72925
C	2.020763	-1.06748	-3.51022	H	-0.32916	5.531499	-0.00268
O	-2.42758	3.080096	3.642943				

12***S*9a*S***-6

C	0.693095	-3.11251	0.039216	C	-3.38803	-3.17657	-1.95101
C	0.967843	-2.39091	1.33886	H	1.143002	2.03	1.418275
C	0.062063	-1.20361	1.646371	H	1.859851	-0.46024	-0.23656
N	-0.07012	-0.20411	0.58874	H	2.014267	-2.06574	1.32057
C	-1.01862	-0.54108	-0.49216	H	0.877674	-3.12841	2.145238
C	-1.38755	-2.00708	-0.75829	H	0.436213	-0.71163	2.551697
C	-0.6644	-3.11519	-0.54585	H	-0.92966	-1.56905	1.942472
C	1.17925	0.353872	0.032506	H	-1.01766	-4.1015	-0.82577
C	-0.3201	-0.00406	-1.7698	H	-0.9955	0.442443	-2.50577
C	0.68813	1.005503	-1.25333	H	0.232129	-0.80136	-2.28684
C	-2.64473	1.53301	-0.60965	H	1.491505	1.160611	-1.98005
C	-2.97907	2.237566	0.469887	H	0.208576	1.971902	-1.05874
C	-3.07771	1.283533	1.579635	H	-2.51346	1.953101	-1.59575
O	-2.86303	0.029106	1.106255	H	-4.37958	-0.60468	-1.27613
O	-3.35024	1.571661	2.730528	H	5.072922	2.047852	0.999831
C	-2.45762	0.100051	-0.27105	H	3.081648	3.112764	0.918583
C	-3.34923	-0.82988	-1.03704	H	2.896904	2.406737	-0.67507
C	-2.75209	-2.006	-1.28077	H	5.67016	-0.03637	-0.18253
C	1.885909	1.346624	0.988357	H	4.974841	1.106115	-1.3439
O	2.510193	0.630458	2.066058	H	4.019236	-0.2619	-0.74604
C	3.853439	0.53105	1.864923	H	-3.13872	4.216033	-0.31598
C	4.2754	1.364434	0.688419	H	-4.26372	3.846407	0.999601
C	3.019764	2.156397	0.38123	H	-2.54449	4.122845	1.348612
O	4.595508	-0.15387	2.549466	H	-4.39792	-2.93693	-2.301
C	4.754389	0.496085	-0.46206	H	-2.80194	-3.48722	-2.82212
C	-3.2456	3.68141	0.633414	H	-3.46948	-4.02372	-1.26242
O	1.556348	-3.80395	-0.50305				

12***S*9a*S***-7							
C	0.683208	-3.77638	0.264979	C	-3.4576	-3.3127	-1.5309
C	1.086609	-3.0754	1.541718	H	2.770067	0.541084	1.367795
C	0.48878	-1.68972	1.774163	H	2.416557	-1.45186	0.080944
N	0.443389	-0.77778	0.630838	H	2.181045	-3.00596	1.545328
C	-0.69364	-0.96762	-0.29243	H	0.81324	-3.73145	2.376869
C	-1.24478	-2.39041	-0.49909	H	1.074854	-1.20443	2.565567
C	-0.66993	-3.5828	-0.29291	H	-0.50843	-1.80592	2.213327
C	1.713128	-0.63238	-0.10253	H	-1.17583	-4.51485	-0.5216
C	-0.13116	-0.46758	-1.6497	H	-0.27199	0.606495	-1.79204
C	1.359468	-0.72802	-1.58374	H	-0.56965	-0.96129	-2.52485
C	-2.12316	1.259863	-0.18172	H	1.558621	-1.74431	-1.94642
C	-2.2429	1.961695	0.943581	H	1.918966	-0.03687	-2.22119
C	-2.28783	0.988041	2.03827	H	-2.09271	1.695488	-1.16977
O	-2.28322	-0.26527	1.515627	H	-4.10555	-0.67133	-0.71928

O	-2.35341	1.262071	3.222297	H	3.184269	3.181761	1.155402
C	-2.0312	-0.19446	0.103067	H	0.991237	2.147307	0.959453
C	-3.0877	-1.0094	-0.58032	H	1.16865	1.996361	-0.7989
C	-2.64315	-2.2337	-0.90115	H	3.335127	4.937356	-0.59204
C	2.455315	0.654558	0.320887	H	1.636123	4.738409	-0.12477
O	3.64879	0.831477	-0.45515	H	2.236419	4.047143	-1.6461
C	3.922425	2.168043	-0.55545	H	-2.31455	3.967771	0.217269
C	2.844551	2.963125	0.136484	H	-3.28306	3.668215	1.668464
C	1.706783	1.961423	0.153373	H	-1.51367	3.771963	1.78321
O	4.908419	2.622949	-1.1103	H	-4.45688	-2.95357	-1.79978
C	2.489207	4.241692	-0.59789	H	-2.97838	-3.67143	-2.44768
C	-2.34433	3.418871	1.164058	H	-3.5836	-4.15773	-0.84654
O	1.413066	-4.61853	-0.25874				

12***R*9a*R***-1							
C	1.273061	-0.77656	2.976137	C	-2.47577	1.573616	4.099744
C	1.253857	-1.93333	1.986967	H	1.709834	-1.81495	-1.7761
C	0.242808	-1.86655	0.84655	H	-0.42434	-0.69236	-1.65801
N	0.214086	-0.51757	0.337985	H	2.261275	-1.98899	1.557235
C	-0.81659	0.372643	0.879534	H	1.096625	-2.85477	2.559627
C	-0.84606	0.451492	2.405439	H	-0.72977	-2.21277	1.20591
C	0.041354	-0.01236	3.298096	H	0.540574	-2.58729	0.076515
C	0.392204	-0.24137	-1.07928	H	-0.09921	0.125968	4.36526
C	-0.26932	1.686134	0.274831	H	-0.99552	2.504856	0.235257
C	0.212527	1.289823	-1.12781	H	0.591125	2.054698	0.851857
C	-2.91722	0.673252	-0.7355	H	1.12456	1.841584	-1.37503
C	-3.46508	-0.31995	-1.43328	H	-0.53183	1.557277	-1.88568
C	-3.32761	-1.52749	-0.61465	H	-2.88048	1.702002	-1.06458
O	-2.75758	-1.19587	0.572855	H	-4.05495	1.117133	1.78196
O	-3.67736	-2.64606	-0.94152	H	4.371146	-1.33624	-2.28754
C	-2.36806	0.183436	0.553761	H	3.277017	-0.89472	-0.18577
C	-3.007	0.851584	1.741818	H	2.901113	0.767126	-0.64267
C	-2.14923	1.000464	2.763051	H	5.850341	0.587578	-2.80857
C	1.717703	-0.7196	-1.70758	H	5.694807	0.442494	-1.04833
O	1.822499	-0.21847	-3.04915	H	4.795263	1.694399	-1.92953
C	3.135144	0.020361	-3.34487	H	-4.12795	0.682066	-3.19801
C	3.997627	-0.31406	-2.15664	H	-3.58402	-0.98351	-3.44851
C	2.991165	-0.25541	-1.02477	H	-5.15259	-0.66489	-2.67904
O	3.524771	0.41894	-4.4296	H	-3.49541	1.97297	4.124394
C	5.147438	0.6577	-1.97153	H	-2.40034	0.811612	4.881976
C	-4.1167	-0.32089	-2.75924	H	-1.79362	2.394774	4.343009
O	2.283697	-0.53995	3.643018				

12***R*9a*R***-2							
C	0.744634	-0.58164	3.085227	C	-1.89753	3.125526	3.336542
C	-0.09339	-1.71898	2.531572	H	1.496972	-2.38025	-1.38863
C	-0.0876	-1.76529	1.009341	H	-0.42238	-0.95024	-1.59286
N	0.176858	-0.48597	0.377926	H	-1.11811	-1.60811	2.90787
C	-0.67625	0.672091	0.686698	H	0.280374	-2.66137	2.948744
C	-0.65748	1.17339	2.13827	H	-1.01194	-2.22252	0.641526
C	0.064942	0.735131	3.179944	H	0.710524	-2.46537	0.735539
C	0.436981	-0.51568	-1.06441	H	0.0187	1.155397	4.176048
C	0.080043	1.718468	-0.17966	H	-0.50609	2.609888	-0.42915
C	0.503305	0.970501	-1.44072	H	0.985559	2.07051	0.336064
C	-2.72433	0.849114	-0.98788	H	1.494289	1.309623	-1.75861
C	-3.49165	-0.18263	-1.33431	H	-0.18435	1.181614	-2.267
C	-3.56827	-1.05741	-0.16088	H	-2.49283	1.683051	-1.63486
O	-2.88067	-0.48942	0.864765	H	-3.63315	2.335181	1.209571
O	-4.1555	-2.12187	-0.11075	H	4.216808	-2.44928	-1.69375
C	-2.23654	0.705974	0.40499	H	3.077431	-1.44346	0.190491
C	-2.67558	1.840489	1.300588	H	3.043281	0.140886	-0.59177
C	-1.76958	2.108608	2.256297	H	6.057544	-0.9317	-2.37895
C	1.692394	-1.30541	-1.49506	H	5.758094	-0.72448	-0.64369
O	1.974216	-1.07226	-2.88238	H	5.167151	0.479459	-1.80741
C	3.326346	-1.11786	-3.08127	H	-4.01211	0.301011	-3.3476
C	4.030368	-1.37209	-1.77349	H	-3.82951	-1.42913	-3.02193
C	2.976923	-0.93761	-0.77481	H	-5.26597	-0.56037	-2.44245
O	3.85534	-0.99501	-4.17336	H	-2.79566	3.737921	3.202607
C	5.324145	-0.5919	-1.63985	H	-1.96557	2.648357	4.319277
C	-4.18619	-0.48282	-2.60337	H	-1.03426	3.798915	3.333511
O	1.879281	-0.73099	3.527437				

12***R*9a*R***-3							
C	-0.94572	2.005401	3.221172	C	-4.39487	3.044298	0.510914
C	0.102721	0.952378	2.981266	H	2.319496	-0.4778	1.710292
C	-0.42016	-0.25909	2.229212	H	0.655108	-0.87445	-0.81689
N	-0.40055	-0.13916	0.781595	H	0.969741	1.414693	2.500923
C	-1.16702	0.963274	0.172113	H	0.430227	0.611246	3.971922
C	-2.16899	1.774863	1.01528	H	-1.4429	-0.49628	2.551503
C	-2.07411	2.21091	2.281872	H	0.152317	-1.14118	2.535063
C	0.883741	-0.26643	0.068407	H	-2.86058	2.817882	2.722459
C	-0.07188	1.949673	-0.30007	H	-0.3082	2.447819	-1.24595
C	1.216227	1.145931	-0.40763	H	0.097124	2.754443	0.427786
C	-1.50905	0.281559	-2.37221	H	1.989363	1.615595	0.211658
C	-1.61181	-0.97794	-2.79338	H	1.581414	1.134563	-1.44068
C	-2.3096	-1.72085	-1.73732	H	-1.06185	1.081012	-2.94398

O	-2.63428	-0.86584	-0.73408	H	-4.01841	1.411336	-1.7545
O	-2.58286	-2.90618	-1.78145	H	4.622284	-1.8889	1.375532
C	-2.09824	0.439022	-1.01747	H	3.905492	-0.09345	-0.0945
C	-3.25772	1.382515	-0.98583	H	3.054705	-1.2265	-1.16163
C	-3.30882	2.084363	0.152937	H	5.516485	-3.71399	-0.05079
C	2.083358	-0.96163	0.754699	H	5.754566	-2.16626	-0.88237
O	1.789912	-2.34174	1.017202	H	4.447466	-3.26089	-1.37839
C	2.93348	-3.0817	0.909694	H	-0.69111	-0.88516	-4.71727
C	4.072754	-2.19574	0.478163	H	-0.45434	-2.41113	-3.85246
C	3.324809	-1.02048	-0.11789	H	-2.0273	-2.03886	-4.58841
O	3.00627	-4.27329	1.157484	H	-5.12346	3.146212	-0.30077
C	4.998834	-2.86862	-0.51664	H	-4.93744	2.704733	1.399139
C	-1.17322	-1.61087	-4.05433	H	-3.9822	4.039014	0.708759
O	-0.87119	2.751673	4.199837				

12***R*9a*R***-4							
C	0.471417	0.899383	3.602088	C	-3.29846	3.207077	2.677265
C	1.031369	-0.28577	2.863074	H	2.6038	-1.16635	0.668033
C	0.08481	-0.98745	1.902787	H	0.438546	-0.33141	-1.32436
N	-0.15479	-0.30927	0.642286	H	1.95694	0.032436	2.377587
C	-0.93479	0.939543	0.677506	H	1.312786	-1.01578	3.633469
C	-1.38642	1.552395	2.014354	H	-0.87495	-1.1858	2.397987
C	-0.80277	1.54325	3.221083	H	0.48225	-1.98783	1.694618
C	0.924983	-0.18172	-0.34949	H	-1.251	2.074117	4.056905
C	-0.00734	1.992814	-0.00119	H	-0.39576	2.341777	-0.96166
C	1.317643	1.282052	-0.23919	H	0.170247	2.888162	0.607103
C	-2.37942	0.765267	-1.53733	H	1.99607	1.456981	0.603052
C	-2.82893	-0.39848	-2.00471	H	1.805842	1.655815	-1.14502
C	-3.16124	-1.22275	-0.83753	H	-2.08178	1.594137	-2.16159
O	-2.94377	-0.49942	0.290378	H	-4.14116	2.135054	0.19737
O	-3.60942	-2.35367	-0.88145	H	4.567861	-2.38275	-0.75153
C	-2.33953	0.762404	-0.05329	H	3.857261	-0.08492	-1.10799
C	-3.18106	1.821403	0.583546	H	2.669633	-0.58148	-2.32886
C	-2.66138	2.226059	1.750751	H	4.913515	-3.41307	-2.98369
C	2.121906	-1.15598	-0.31493	H	5.214814	-1.6699	-3.10313
O	1.686717	-2.49381	-0.60237	H	3.695483	-2.36248	-3.70706
C	2.667931	-3.15752	-1.28363	H	-2.74424	-0.09778	-4.11667
C	3.824663	-2.22761	-1.54209	H	-2.4171	-1.75485	-3.58775
C	3.15628	-0.87628	-1.39021	H	-4.07707	-1.12384	-3.56568
O	2.606854	-4.33252	-1.6034	H	-4.216	3.624646	2.248648
C	4.446079	-2.4252	-2.91134	H	-3.56521	2.730991	3.626277
C	-3.02733	-0.86767	-3.3916	H	-2.6205	4.042454	2.880656
O	1.075711	1.365137	4.571934				

12***R*9a*R***-5							
C	-2.32468	0.822044	2.771048	C	-5.15859	-1.33123	-0.09627
C	-0.89162	0.385354	3.048232	H	1.108117	2.407596	1.153176
C	-0.22621	-0.49128	1.995673	H	1.305934	-0.22522	-0.42265
N	-0.39223	0.170293	0.727247	H	-0.89278	-0.14455	4.007821
C	-1.49538	-0.26437	-0.11766	H	-0.3008	1.301581	3.176423
C	-2.84211	-0.36382	0.598011	H	-0.67605	-1.48932	2.016583
C	-3.1996	0.10362	1.804662	H	0.823577	-0.649	2.257201
C	0.729119	0.63947	-0.07156	H	-4.20754	-0.03209	2.184641
C	-1.49072	0.938415	-1.08453	H	-1.96914	0.746381	-2.05068
C	-0.00848	1.30104	-1.25072	H	-2.01238	1.799713	-0.64253
C	-0.68847	-1.7494	-2.16389	H	0.090236	2.391949	-1.26538
C	0.268591	-2.67167	-2.08186	H	0.382106	0.922991	-2.20173
C	0.168702	-3.25858	-0.74206	H	-0.88976	-1.15459	-3.04302
O	-0.88094	-2.69692	-0.08864	H	-3.28843	-2.71754	-1.72604
O	0.899175	-4.12443	-0.29812	H	3.565631	3.596757	1.163484
C	-1.44675	-1.65229	-0.89118	H	2.319672	3.100092	-0.85914
C	-2.91163	-1.95283	-1.0602	H	3.079417	1.5044	-1.01181
C	-3.67719	-1.23043	-0.2271	H	5.940245	3.011097	0.733681
C	1.683846	1.628405	0.636197	H	5.174485	3.466657	-0.79923
O	2.490185	0.95522	1.615527	H	5.46985	1.758437	-0.41533
C	3.716988	1.550524	1.691747	H	1.16355	-2.56642	-4.01714
C	3.810837	2.650142	0.668395	H	2.282705	-2.94804	-2.70012
C	2.711326	2.249099	-0.29424	H	1.149852	-4.18112	-3.29201
O	4.582546	1.234269	2.490111	H	-5.58098	-2.00404	-0.85039
C	5.173051	2.725288	0.006113	H	-5.43936	-1.71993	0.887766
C	1.268183	-3.11485	-3.07542	H	-5.6243	-0.34961	-0.23165
O	-2.84184	1.722734	3.438561				

12***R*9a*R***-6							
C	2.893316	-0.10672	2.042882	C	2.668632	4.32115	1.254087
C	1.842276	-1.1284	1.707317	H	-1.96525	-2.17483	0.583535
C	0.428438	-0.61803	1.925607	H	-1.59905	-0.15534	-0.63284
N	-0.15892	0.075058	0.796942	H	2.019459	-1.49743	0.69393
C	0.554958	1.252197	0.257549	H	2.000588	-1.97399	2.388992
C	1.704068	1.901055	1.055144	H	0.397216	0.045435	2.800412
C	2.675549	1.337056	1.791054	H	-0.22209	-1.44929	2.217707
C	-0.68732	-0.69442	-0.33978	H	3.43869	1.947399	2.266972
C	1.152391	0.727904	-1.0706	H	1.200717	1.485841	-1.85952
C	0.290328	-0.45664	-1.49284	H	2.180255	0.36521	-0.9376
C	-1.2281	2.589692	-1.19909	H	0.934032	-1.31604	-1.69892
C	-2.53391	2.600473	-0.93563	H	-0.25109	-0.22736	-2.41798

C	-2.66962	2.535674	0.523984	H	-0.80562	2.641958	-2.19183
O	-1.43339	2.544827	1.085766	H	0.145932	4.695475	0.060362
O	-3.72508	2.505155	1.129792	H	-1.62013	-4.85419	0.269593
C	-0.43323	2.495976	0.052158	H	0.094973	-3.37421	1.094442
C	0.454172	3.67932	0.267559	H	0.738589	-3.09835	-0.52963
C	1.612889	3.346173	0.848309	H	-0.53947	-6.4394	-1.30527
C	-1.19055	-2.15104	-0.19465	H	0.656227	-5.91838	-0.10416
O	-1.83227	-2.5365	-1.4268	H	0.678356	-5.24202	-1.74574
C	-1.70771	-3.88405	-1.61098	H	-3.38404	2.717198	-2.89058
C	-0.91379	-4.47779	-0.47933	H	-4.33054	1.782785	-1.72287
C	-0.17999	-3.25629	0.0436	H	-4.30115	3.556142	-1.63026
O	-2.19354	-4.49159	-2.54966	H	2.404147	5.343251	0.961782
C	0.026109	-5.57879	-0.93231	H	2.807573	4.315391	2.340032
C	-3.69807	2.667943	-1.84295	H	3.623093	4.079295	0.775107
O	3.980918	-0.45394	2.507196				

12***R*9a*R***-7							
C	-0.47586	0.70821	2.905873	C	-4.2104	2.658105	1.202737
C	-0.24433	-0.77175	2.719657	H	2.228159	-0.04274	1.782995
C	-0.51019	-1.30012	1.31447	H	1.317069	-0.31889	-1.12457
N	-0.2374	-0.41319	0.197262	H	0.787779	-0.98925	3.011388
C	-1.24436	0.619391	-0.11364	H	-0.87947	-1.30071	3.440624
C	-2.02786	1.239599	1.060272	H	-1.55689	-1.62473	1.275707
C	-1.70641	1.313556	2.358856	H	0.0447	-2.23613	1.179999
C	1.128025	0.049406	-0.10573	H	-2.32399	1.81723	3.09281
C	-0.3964	1.759191	-0.74656	H	-0.31037	1.660558	-1.833
C	0.990704	1.56638	-0.1662	H	-0.76616	2.769819	-0.53942
C	-2.19696	0.069203	-2.51696	H	1.055472	2.017965	0.830076
C	-2.369	-1.17887	-2.94894	H	1.751782	2.031946	-0.8006
C	-2.79281	-1.96849	-1.78875	H	-1.9131	0.896796	-3.14993
O	-2.919	-1.14544	-0.7161	H	-4.42571	1.273658	-1.26884
O	-3.03733	-3.16081	-1.79937	H	4.879029	-0.58346	1.84325
C	-2.44149	0.166221	-1.0564	H	3.87254	1.168421	0.493018
C	-3.5261	1.134541	-0.68496	H	3.684714	0.074893	-0.89141
C	-3.29581	1.709602	0.505236	H	6.604198	-1.7026	0.4519
C	2.342015	-0.3773	0.74738	H	6.441178	-0.01902	-0.0798
O	2.501853	-1.80196	0.743172	H	5.715307	-1.33436	-1.02622
C	3.830247	-2.11319	0.817495	H	-1.92686	-1.00064	-5.02993
C	4.650422	-0.85	0.804948	H	-1.463	-2.54531	-4.30075
C	3.666223	0.133286	0.204438	H	-3.17167	-2.19527	-4.63487
O	4.262205	-3.25032	0.903585	H	-5.07701	2.907718	0.581085
C	5.922566	-0.98048	-0.0102	H	-4.58632	2.224183	2.134814
C	-2.22514	-1.75979	-4.29966	H	-3.6907	3.593271	1.435528

O	0.271922	1.401663	3.594255				

12***R*9a*R***-8							
C	-1.79612	0.567156	2.466035	C	-4.90202	-1.50267	-0.12085
C	-0.51335	-0.12682	2.862863	H	0.454918	2.13966	1.313004
C	0.136652	-0.99229	1.786491	H	1.918054	0.028154	-0.35344
N	0.068257	-0.48502	0.434518	H	-0.72853	-0.74053	3.745813
C	-1.17768	-0.61129	-0.31971	H	0.182067	0.650732	3.191749
C	-2.5024	-0.66838	0.451498	H	-0.32836	-1.98595	1.821704
C	-2.79494	-0.15674	1.654511	H	1.181219	-1.18363	2.061057
C	0.983616	0.537352	-0.08266	H	-3.78274	-0.21066	2.095977
C	-1.17679	0.690546	-1.15032	H	-1.71537	0.615877	-2.10077
C	0.293364	1.008511	-1.36517	H	-1.62812	1.520545	-0.58886
C	-0.60843	-1.94859	-2.52996	H	0.428306	2.067706	-1.59645
C	0.323306	-2.89921	-2.57135	H	0.681527	0.446063	-2.22159
C	0.274271	-3.59057	-1.27867	H	-0.83357	-1.27572	-3.34429
O	-0.73256	-3.06625	-0.53343	H	-3.22703	-2.84034	-1.98088
O	0.996302	-4.51296	-0.94771	H	2.234718	4.117132	1.923585
C	-1.28483	-1.92514	-1.20995	H	1.587341	3.530547	-0.33452
C	-2.76706	-2.141	-1.29596	H	2.916413	2.363581	-0.48692
C	-3.43056	-1.45216	-0.35621	H	4.685856	4.515877	1.910178
C	1.355921	1.707087	0.861652	H	4.033043	4.896234	0.306154
O	2.212681	1.232415	1.915924	H	4.908877	3.376629	0.582373
C	3.089377	2.212878	2.281808	H	1.120672	-2.66223	-4.53739
C	2.896505	3.416719	1.401297	H	2.293597	-3.17845	-3.31655
C	2.189799	2.798567	0.211308	H	1.094383	-4.3291	-3.9425
O	3.886013	2.109987	3.199098	H	-5.40587	-2.12196	-0.87079
C	4.203063	4.08832	1.024887	H	-5.12559	-1.9277	0.862933
C	1.256473	-3.28728	-3.64887	H	-5.33575	-0.49885	-0.17621
O	-2.08892	1.683231	2.895534				

Spectroscopic data

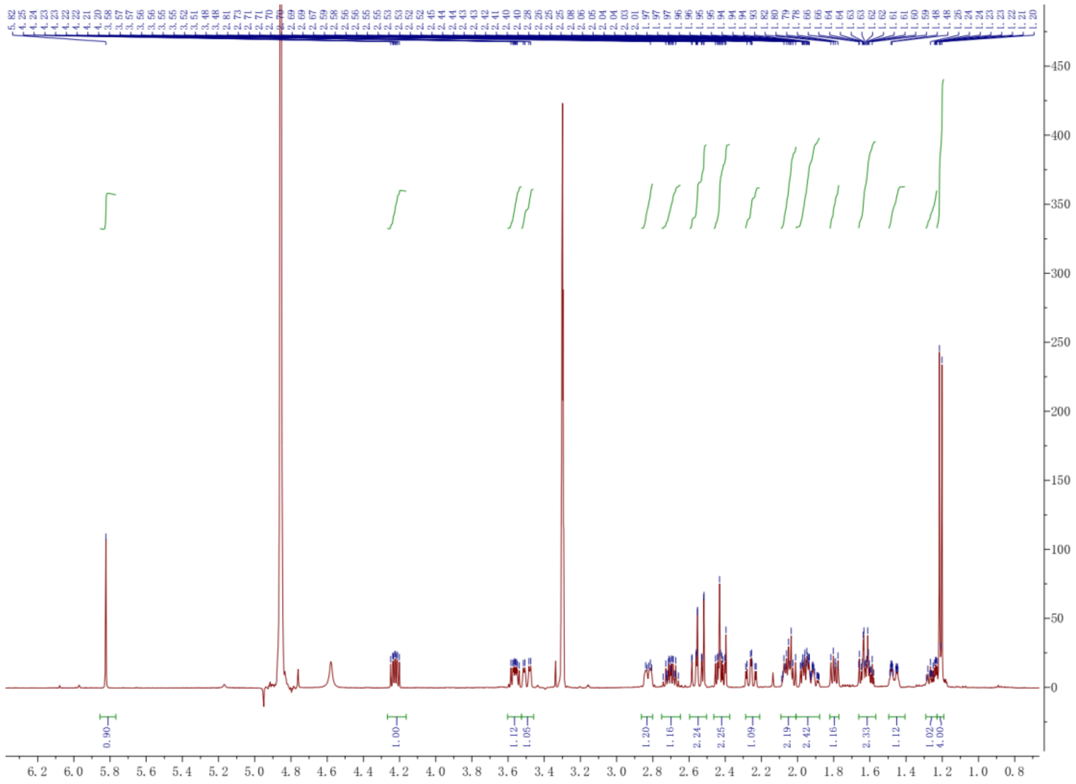

Figure S5. ^{1}H NMR Spectrum of **1** in CD_3OD

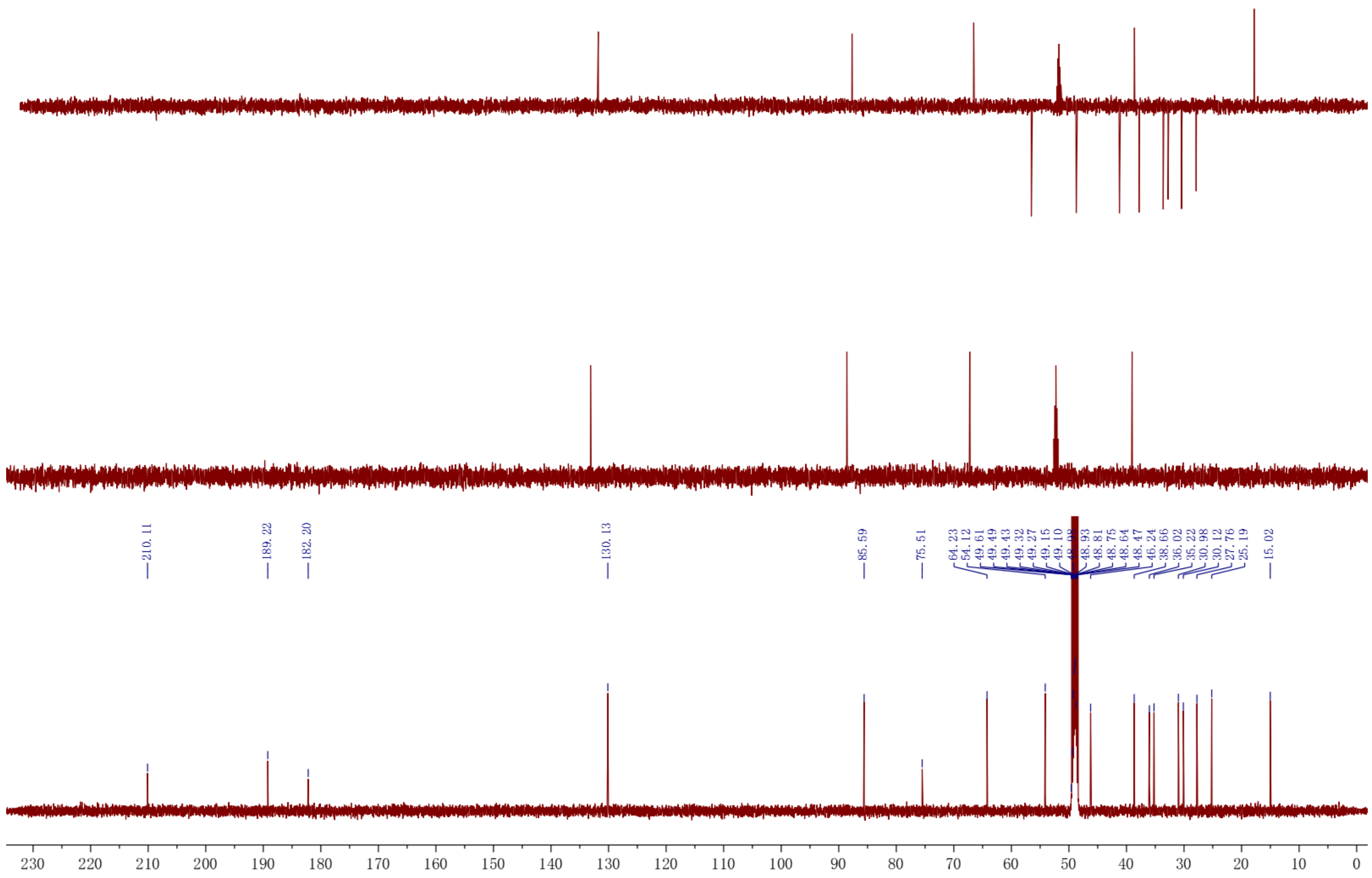

Figure S6. ^{13}C NMR Spectrum of **1** in CD_3OD

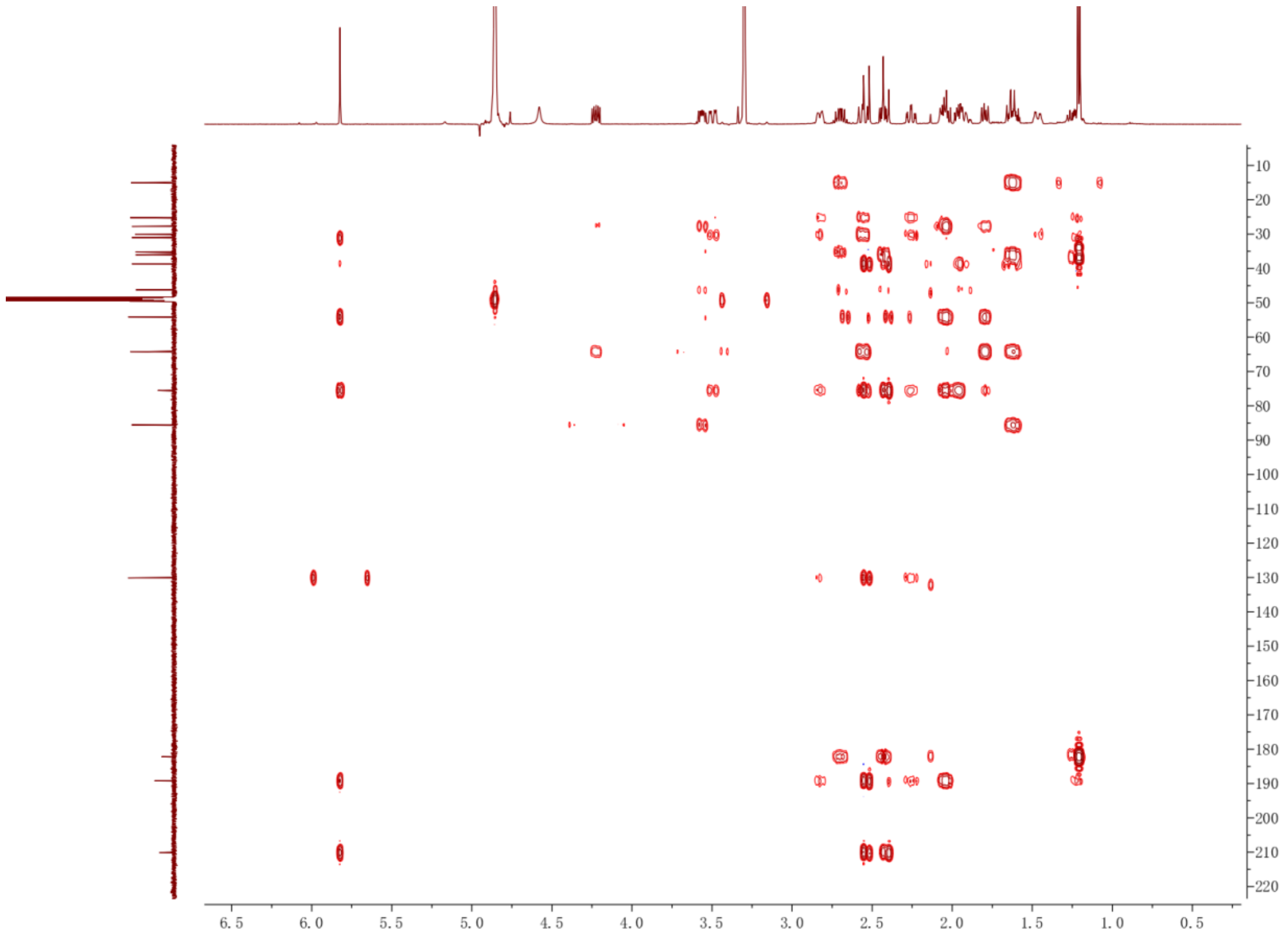

Figure S7. HMBC Spectrum of **1** in CD_3OD

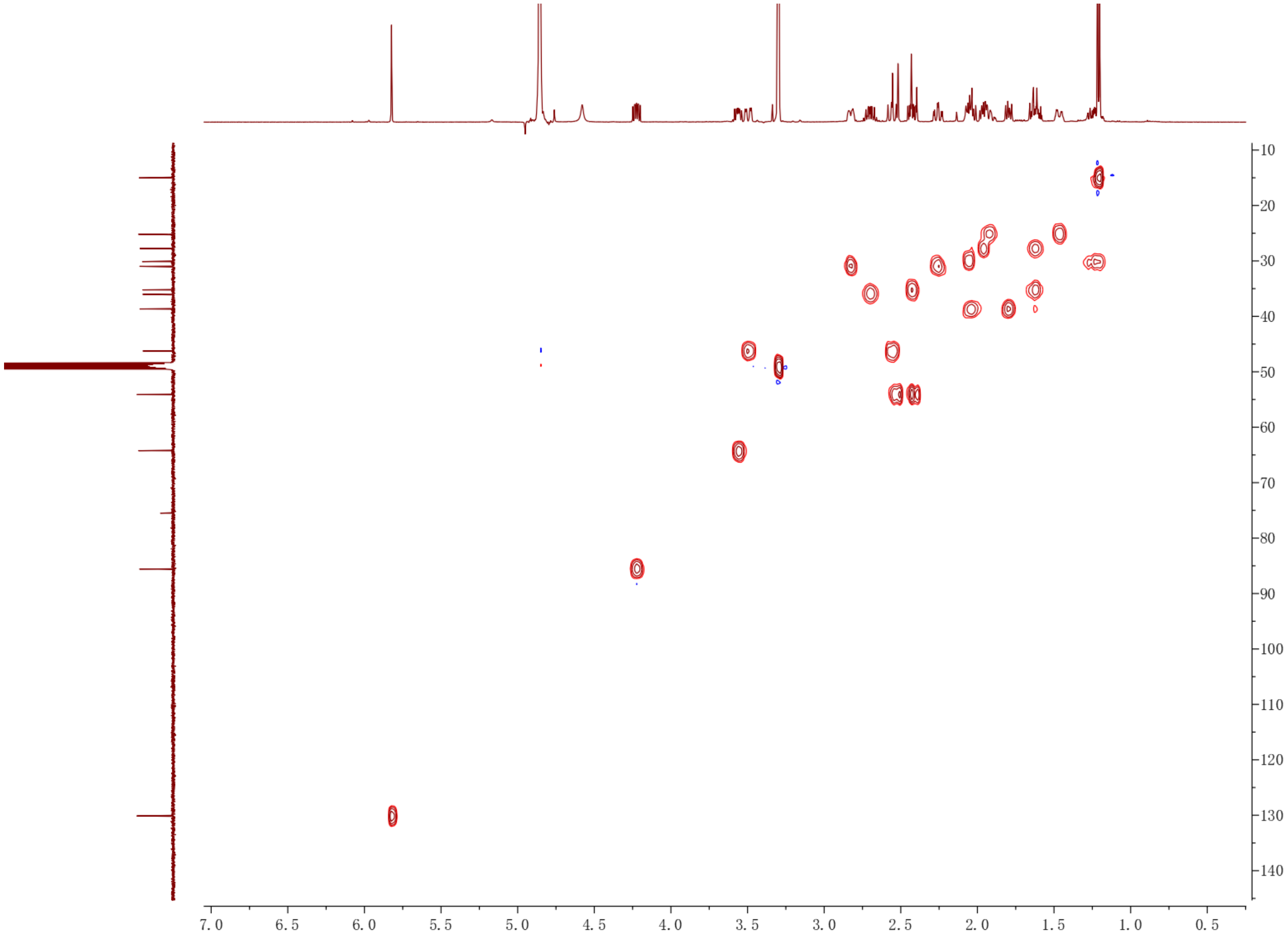

Figure S8. HSQC Spectrum of **1** in CD_3OD

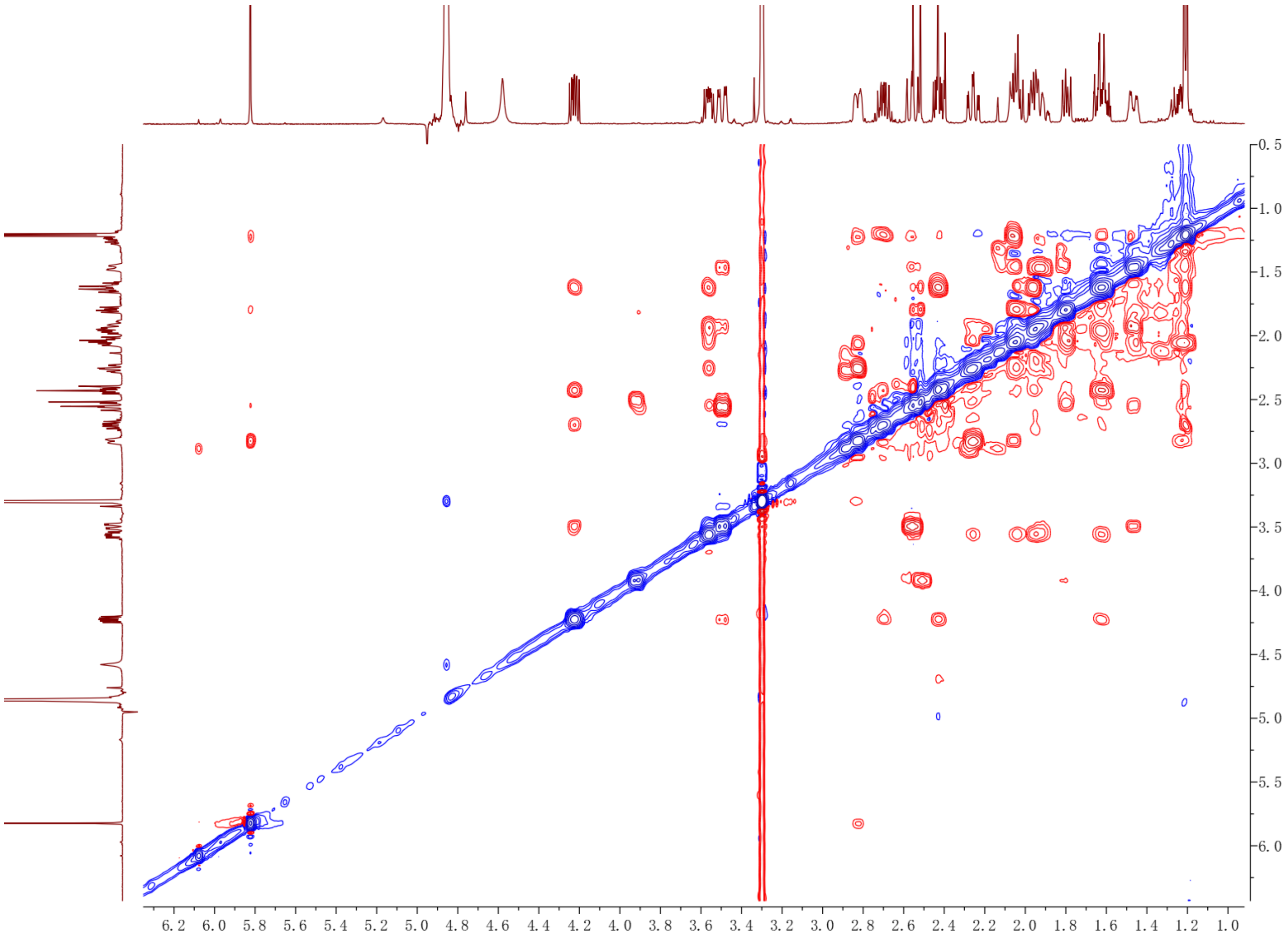

Figure S9. ROESY Spectrum of **1** in CD_3OD

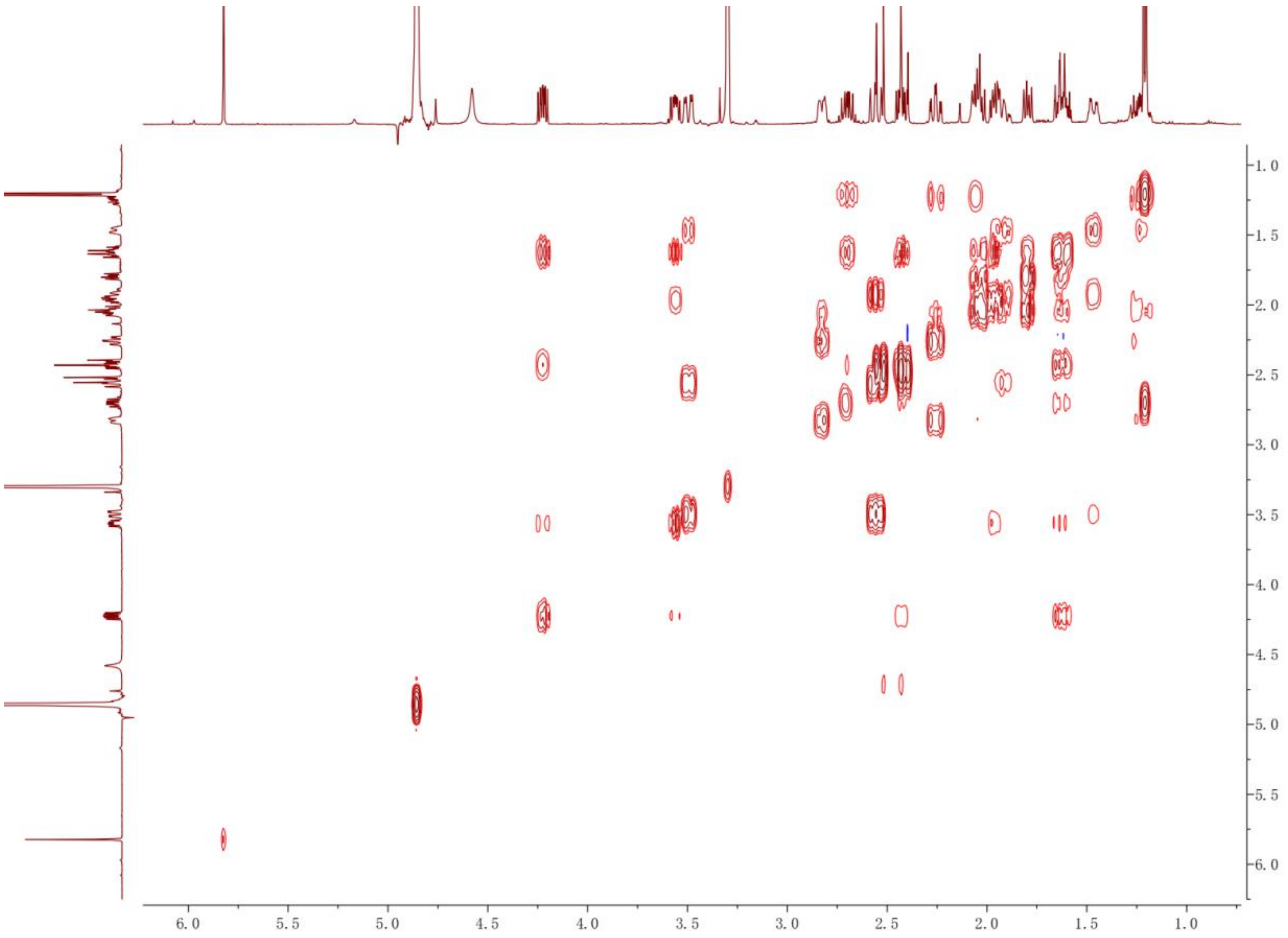

Figure S10. COSY Spectrum of **1** in CD_3OD

Qualitative Analysis Report

**Data Filename**	20221128ESIA3.d	**Sample Name**	wsj-18
**Sample Type**	Sample	**Position**	
**Instrument Name**	Agilent G6230 TOF MS	**User Name**	KIB
**Acq Method**	ESI.m	**Acquired Time**	11/28/2022 8:49:12 PM
**IRM Calibration Status**	Success	**DA Method**	ESI.m
**Comment**			

Sample Group **Info.**
Acquisition SW 6200 series TOF/6500 series
Version Q-TOF B.05.01 (B5125.2)

User Spectra

Fragmentor Voltage	Collision Energy	Ionization Mode
200	0	ESI

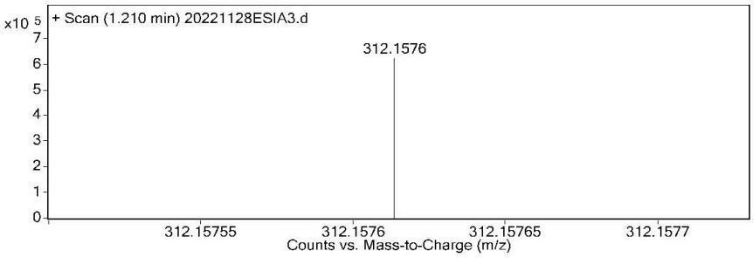

Peak List

m/z	z	Abund	Formula	Ion
121.0509	1	81054.84		
131.0854	1	158299.05		
247.0706	1	139761.19		
288.2893	1	42073		
290.1753	1	292029.31		
291.1783	1	51655.18		
312.1576	1	621877	C17 H23 N Na O3	M+
313.1603	1	104958.73	C17 H23 N Na O3	M+
328.1328	1	82456.95		
922.0098	1	140726.14		

Formula Calculator Element Limits

Element	Min	Max
C	0	200
H	0	400
O	0	10
N	1	1
Na	1	1

Formula Calculator Results

Formula	CalculatedMass	Mz	Diff.(mDa)	Diff. (ppm)	DBE
C17 H23 N Na O3	312.1576	312.1576	0.0	0.1	6.5

--- End Of Report ---

Figure S11. HRESIMS Spectrum of **1**

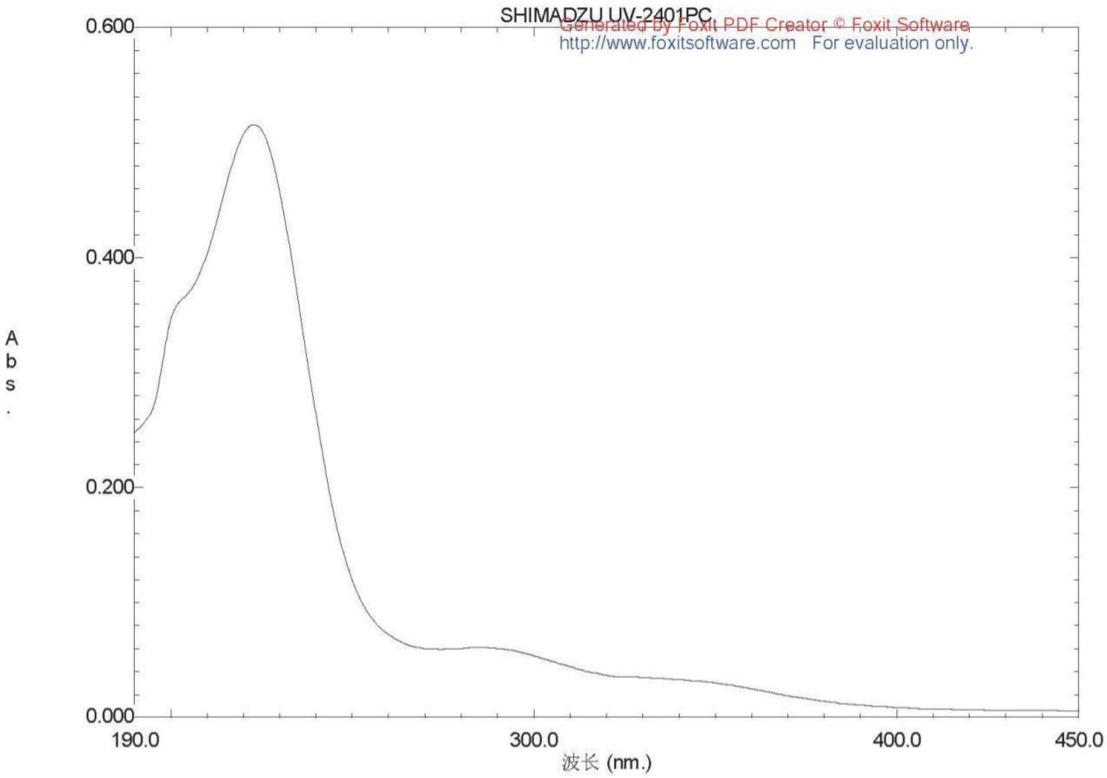

文件名: WSJ-18

WSJ-18 ———

样品浓度：0.0760毫克/毫升
溶剂：甲醇

创建于: 17:14 22-11-25
数据: 原始

测量模式: Abs.
扫描速度: 中速
狭缝: 5.0
采样间隔: 0.5

否.	波长 (nm.)	Abs.
1	204.50	0.3676
2	223.00	0.5155
3	285.00	0.0607
4	333.50	0.0339

Figure S12. UV *S*pectrum of **1**

Rudolph Research Analytical

This sample was measured on an Autopol VI, Serial #91058
Manufactured by Rudolph Research Analytical, Hackettstown, NJ, USA.

Measurement Date : Friday, 25-NOV-2022

Set Temperature : OFF

Time Delay : Disabled

Delay between Measurement : Disabled

n	Average	Std.Dev.	% RSD	Maximum	Minimum
5	-7.00	0.00	0.00	-7.00	-7.00

S.No	Sample ID	Time	Result	Scale	OR °Arc	WLG.nm	Lg.mm	Conc.g/100ml	Temp.
1	wsj-18	04:09:48 PM	-7.00	SR	-0.014	589	100.00	0.200	19.5
2	wsj-18	04:09:54 PM	-7.00	SR	-0.014	589	100.00	0.200	19.5
3	wsj-18	04:10:00 PM	-7.00	SR	-0.014	589	100.00	0.200	19.5
4	wsj-18	04:10:05 PM	-7.00	SR	-0.014	589	100.00	0.200	19.5
5	wsj-18	04:10:11 PM	-7.00	SR	-0.014	589	100.00	0.200	19.5

Figure S13. Optical Rotation Spectrum of **1**

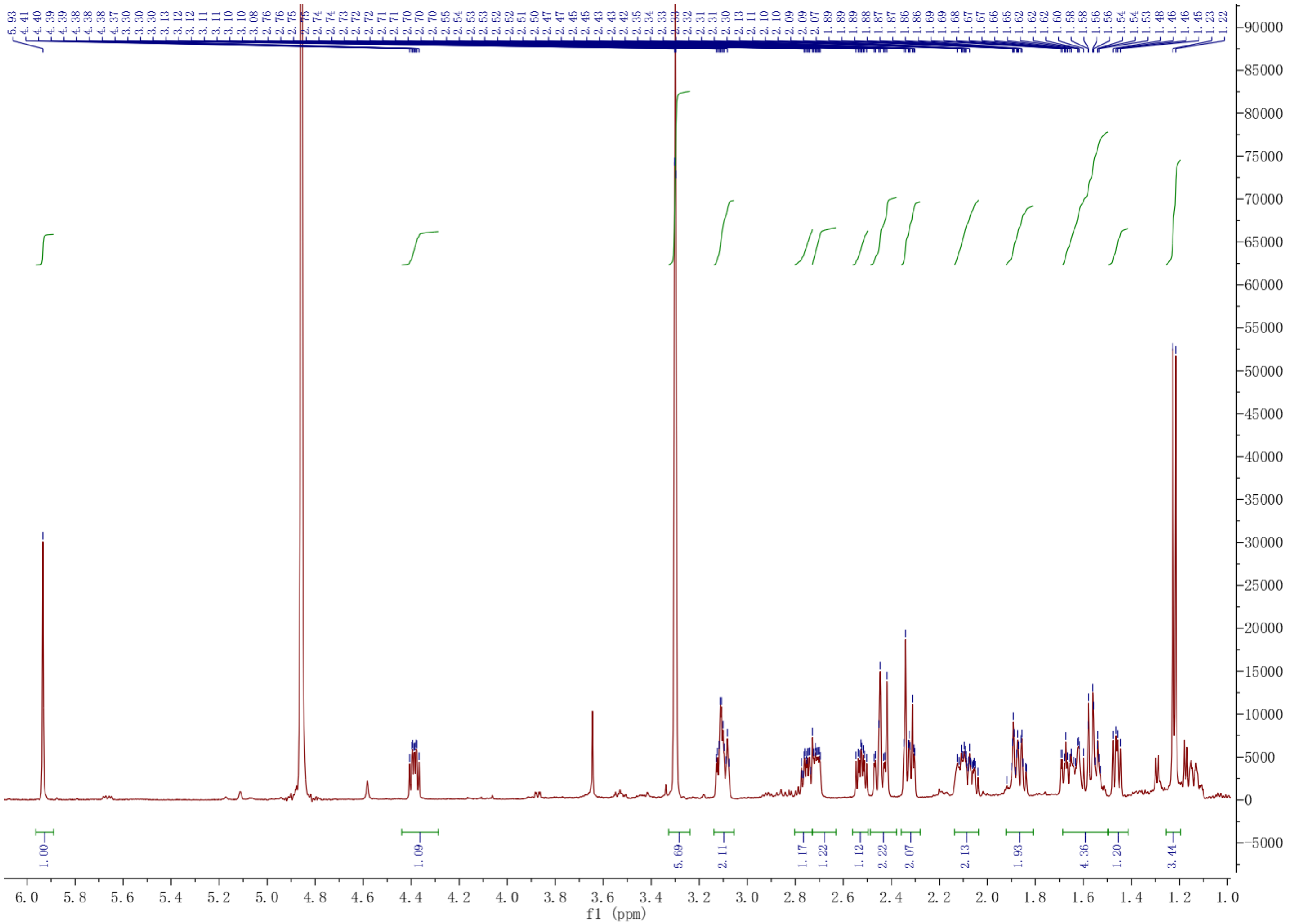

Figure S14. ^{1}H NMR Spectrum of **2** in CD_3OD

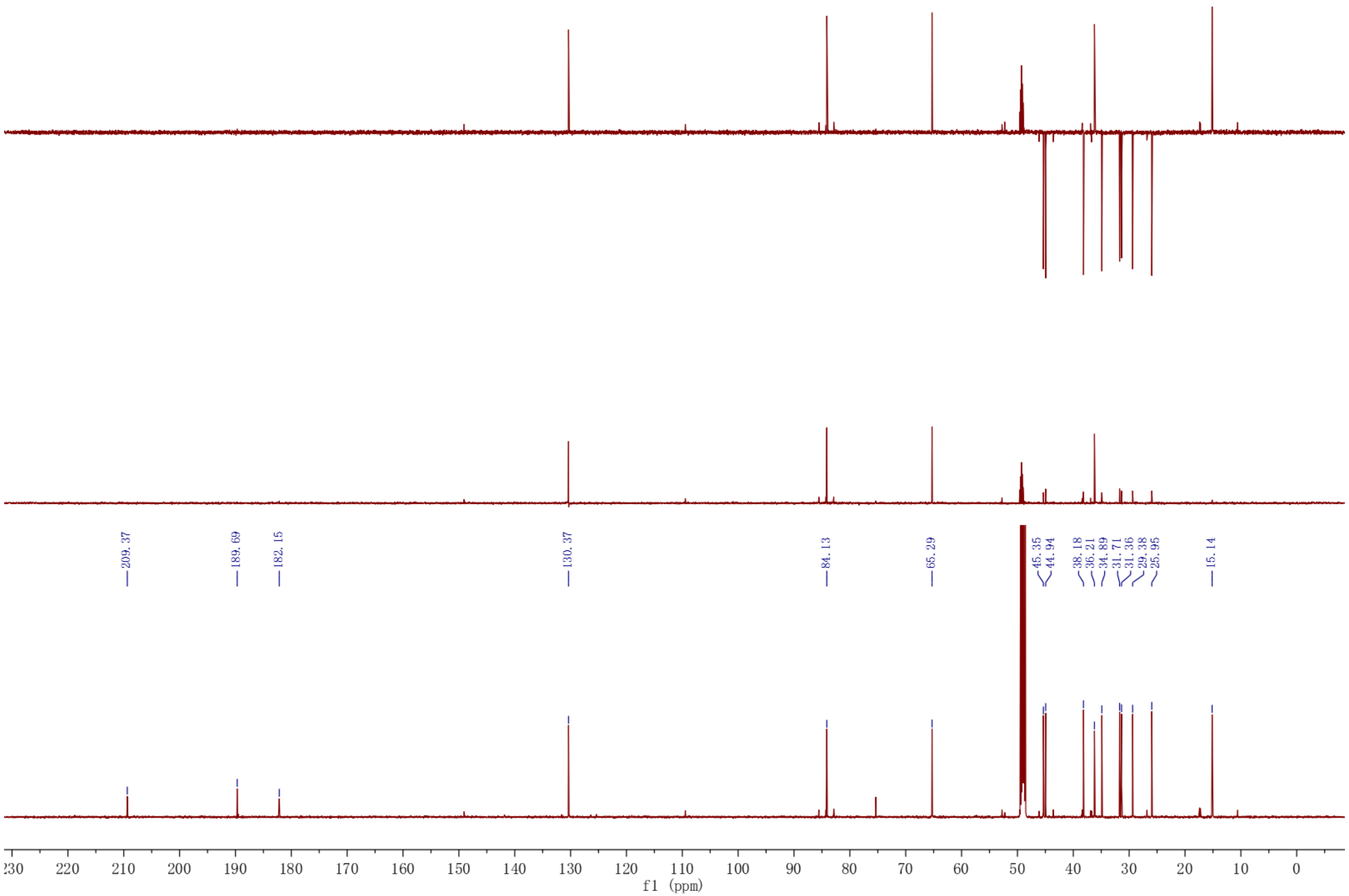

Figure S15. ^{13}C NMR Spectrum of **2** in CD_3OD

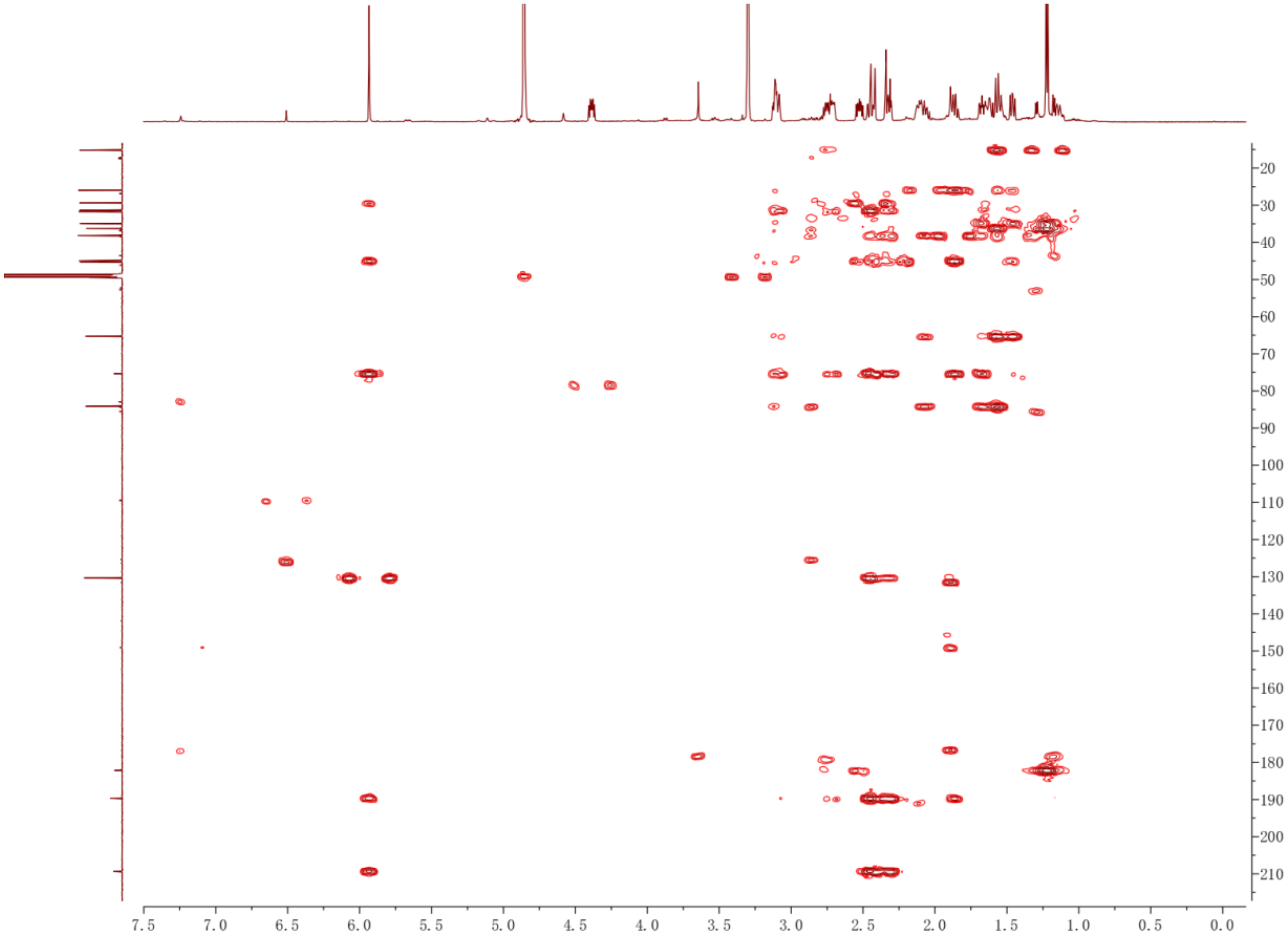

Figure S16. HMBC Spectrum of **2** in CD_3OD

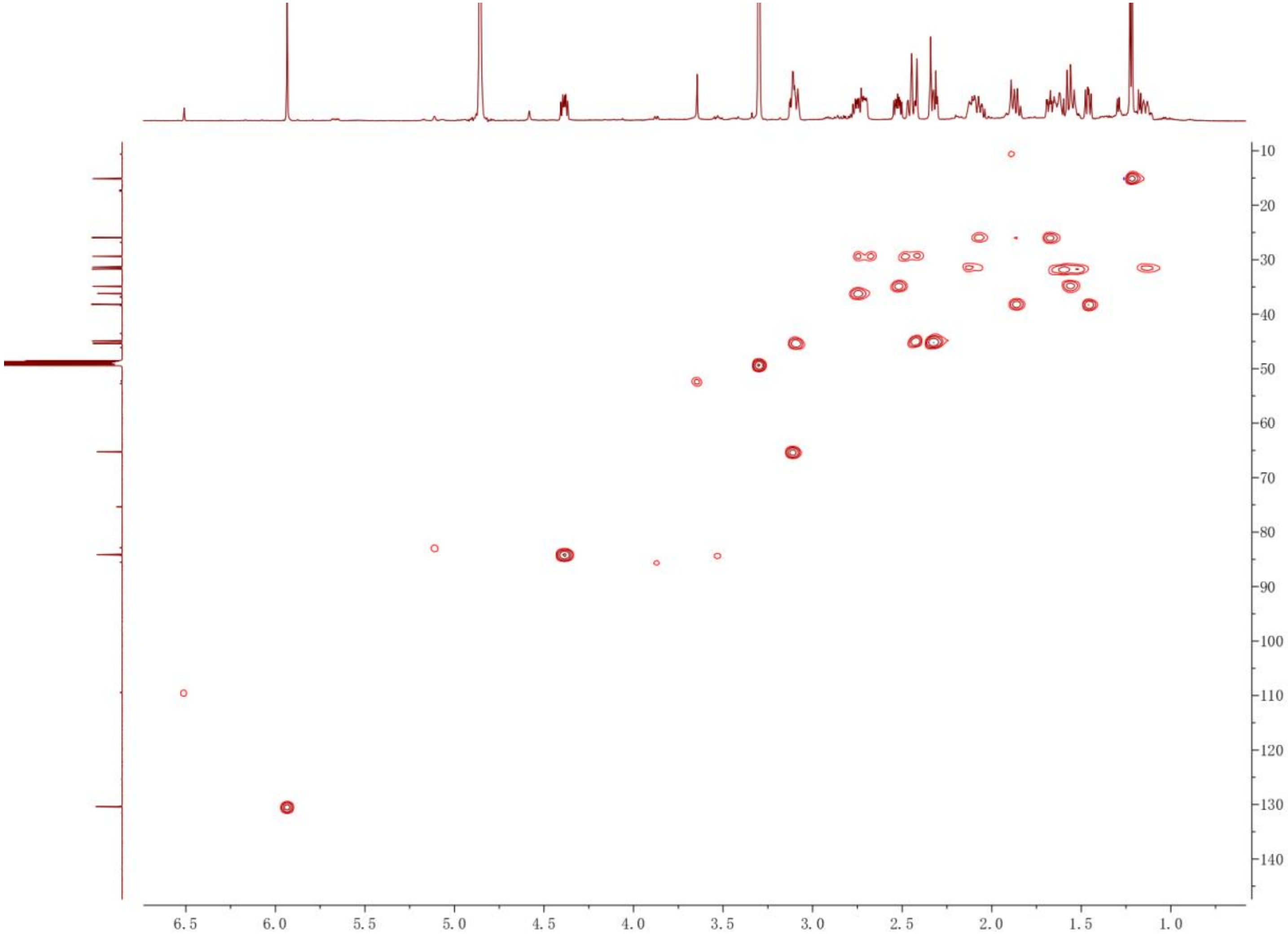

Figure S17. HSQC Spectrum of **2** in CD_3OD

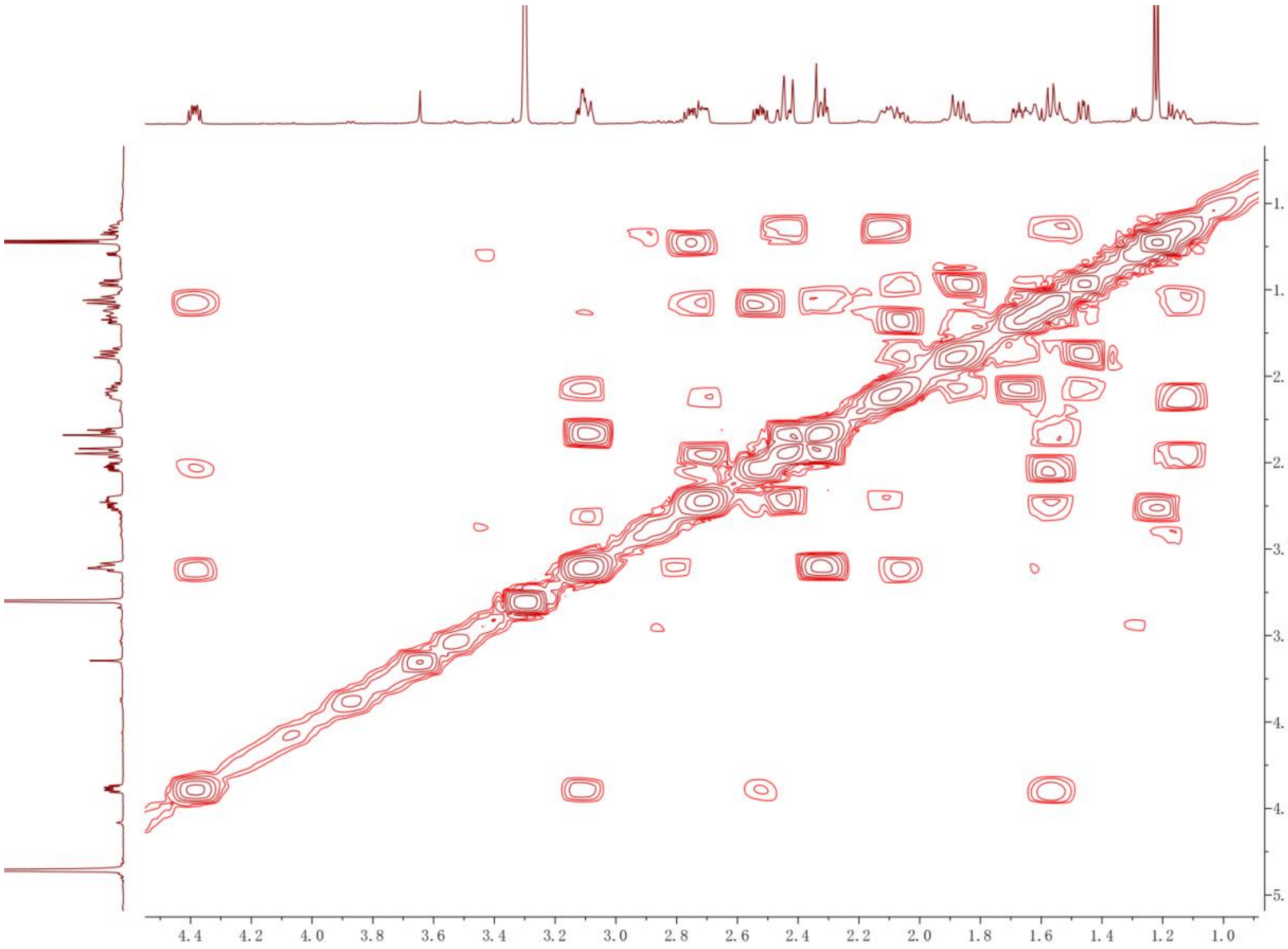

Figure S18. ^{1}H-^{1}H COSY Spectrum of **2** in CD_3OD

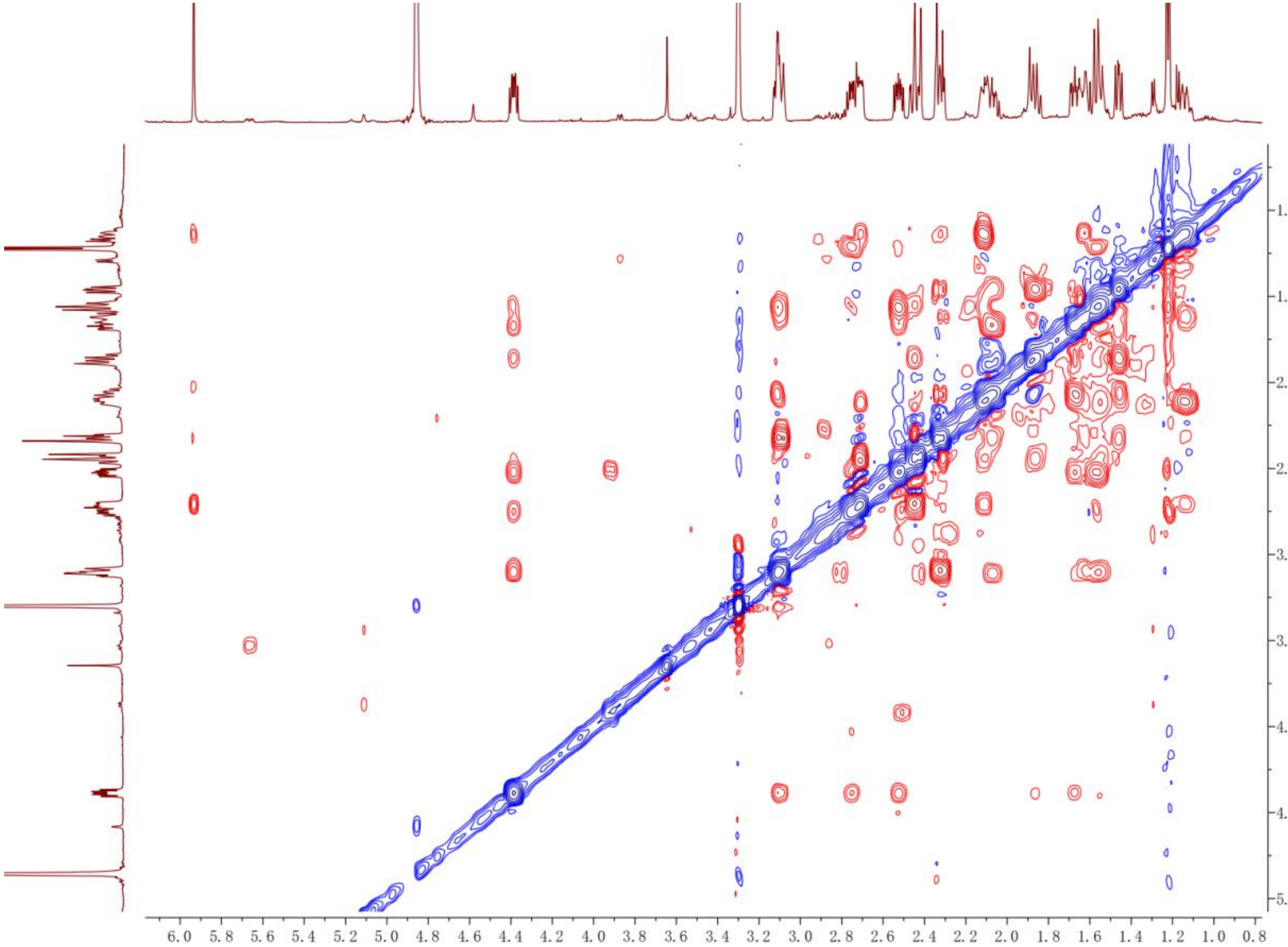

Figure S19. ROESY Spectrum of **2** in CD_3OD

Qualitative Analysis Report

**Data Filename**	20221128ESIA4.d	**Sample Name**	wsj-91b
**Sample Type**	Sample	**Position**	
**Instrument Name**	Agilent G6230 TOF MS	**User Name**	KIB
**Acq Method**	ESI.m	**Acquired Time**	11/28/2022 8:51:05 PM
**IRM Calibration Status**	Success	**DA Method**	ESI.m
**Comment**			

Sample Group **Info.**
Acquisition SW 6200 series TOF/6500 series
Version Q-TOF B.05.01 (B5125.2)

User Spectra

Fragmentor Voltage	Collision Energy	Ionization Mode
300	0	ESI

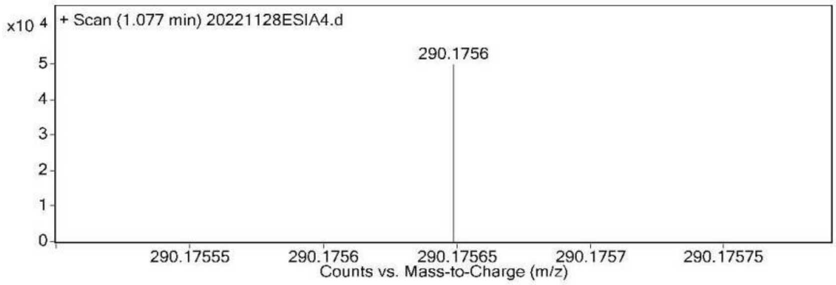

Peak List

m/z	z	Abund	Formula	Ion
290.1756	1	49812.51	C17 H24 N O3	M+
462.2133	1	103674.5		
463.2174	1	39616.31		
500.1902	1	101650.88		
501.1937	1	27168.71		
502.2052	1	94433.92		
503.2101	1	120683.65		
504.2151	1	59683.9		
922.0098	1	208940.86		
923.0122	1	32825.27		

Formula Calculator Element Limits

Element	Min	Max
C	0	200
H	0	400
O	0	10
N	1	1

Formula Calculator Results

Formula	CalculatedMass	Mz	Diff.(mDa)	Diff. (ppm)	DBE
C17 H24 N O3	290.1756	290.1756	0.0	0.1	6.5

--- End Of Report ---

Figure S20. HRESIMS Spectrum of **2**

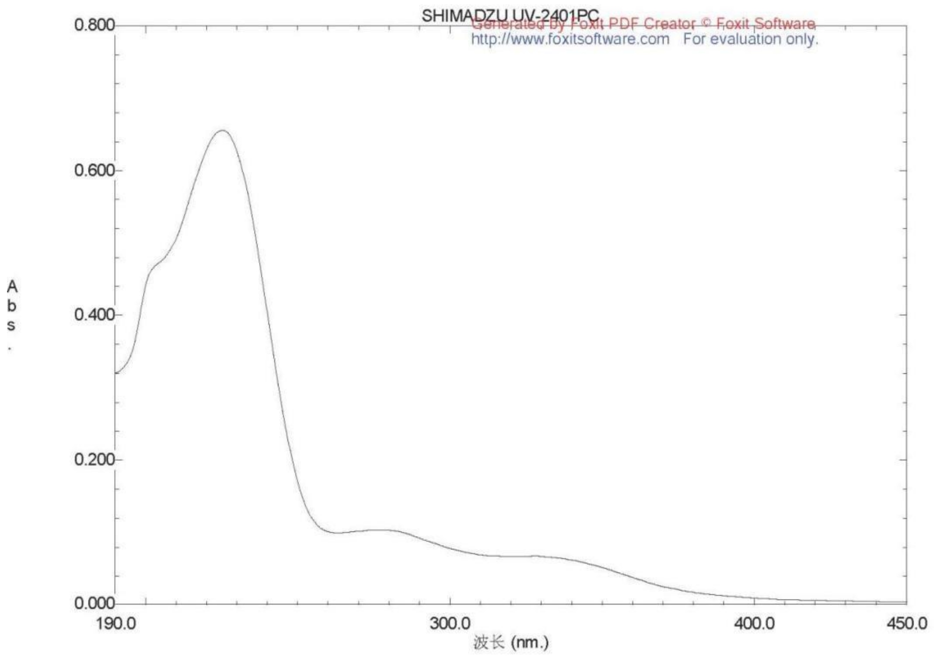

文件名: WSJ-91B

WSJ-91B———

样品浓度：0.0193毫克/毫升
溶剂：甲醇

创建于: 17:19 22-11-25
数据: 原始

测量模式: Abs.
扫描速度: 中速
狭缝: 5.0
采样间隔: 0.5

否.	波长 (nm.)	Abs.
1	206.00	0.4784
2	225.50	0.6556
3	275.50	0.1027
4	327.00	0.0666

Figure S21. UV Spectrum of **2**

Rudolph Research Analytical

This sample was measured on an Autopol VI, Serial #91058
Manufactured by Rudolph Research Analytical, Hackettstown, NJ, USA.

Measurement Date : Friday, 25-NOV-2022

Set Temperature : OFF

Time Delay : Disabled

Delay between Measurement : Disabled

n	Average	Std.Dev.	% RSD	Maximum	Minimum
5	46.67	0.00	0.00	46.67	46.67

S.No	Sample ID	Time	Result	Scale	OR °Arc	WLG.nm	Lg.mm	Conc.g/100ml	Temp.
1	wsj-91b	04:16:36 PM	46.67	SR	0.028	589	100.00	0.060	19.9
2	wsj-91b	04:16:43 PM	46.67	SR	0.028	589	100.00	0.060	19.9
3	wsj-91b	04:16:49 PM	46.67	SR	0.028	589	100.00	0.060	19.8
4	wsj-91b	04:16:55 PM	46.67	SR	0.028	589	100.00	0.060	19.8
5	wsj-91b	04:17:01 PM	46.67	SR	0.028	589	100.00	0.060	19.8

Figure S22. Optical Rotation Spectrum of **2**

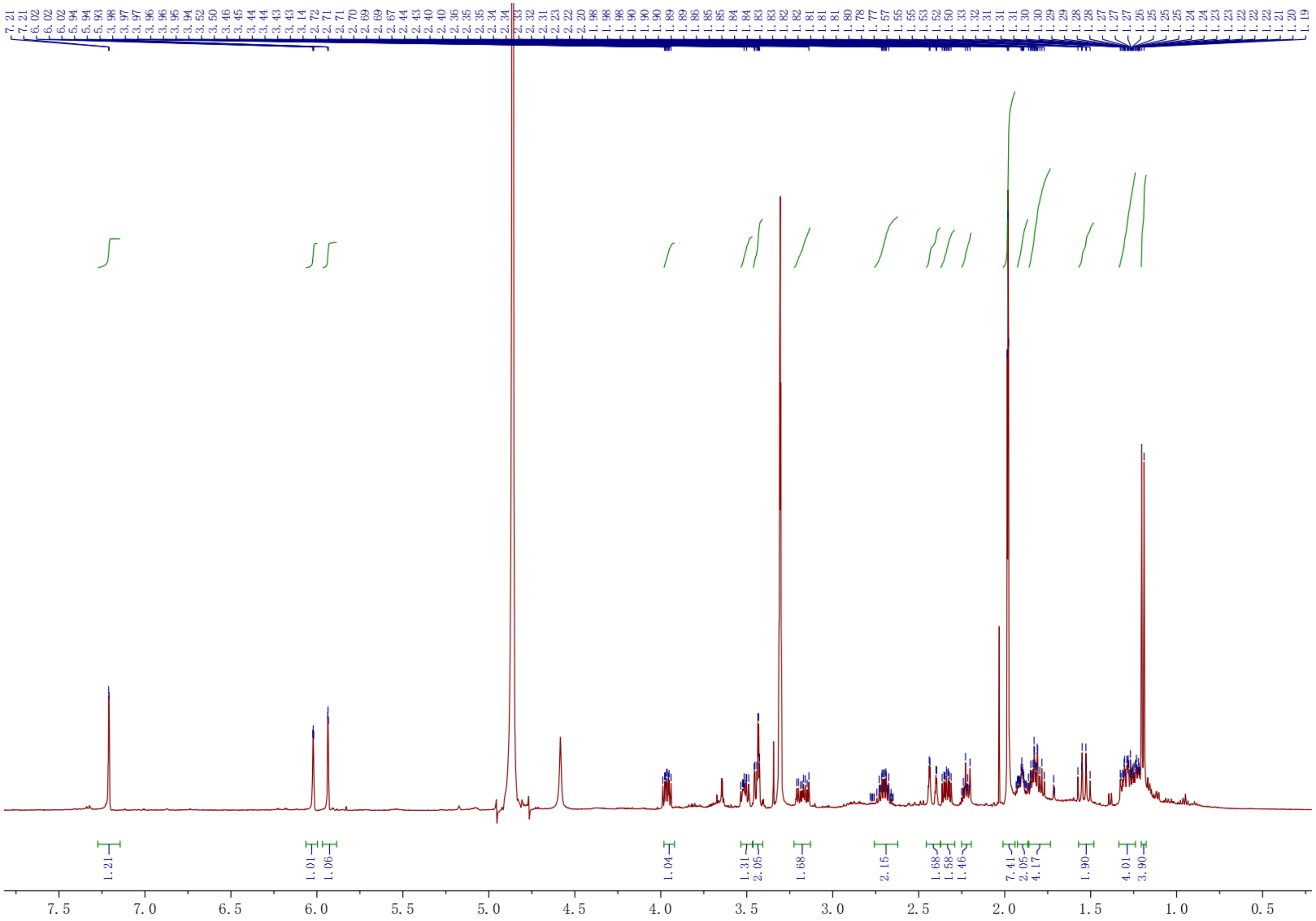

Figure S23. ^{1}H NMR Spectrum of **3** in CD_3OD

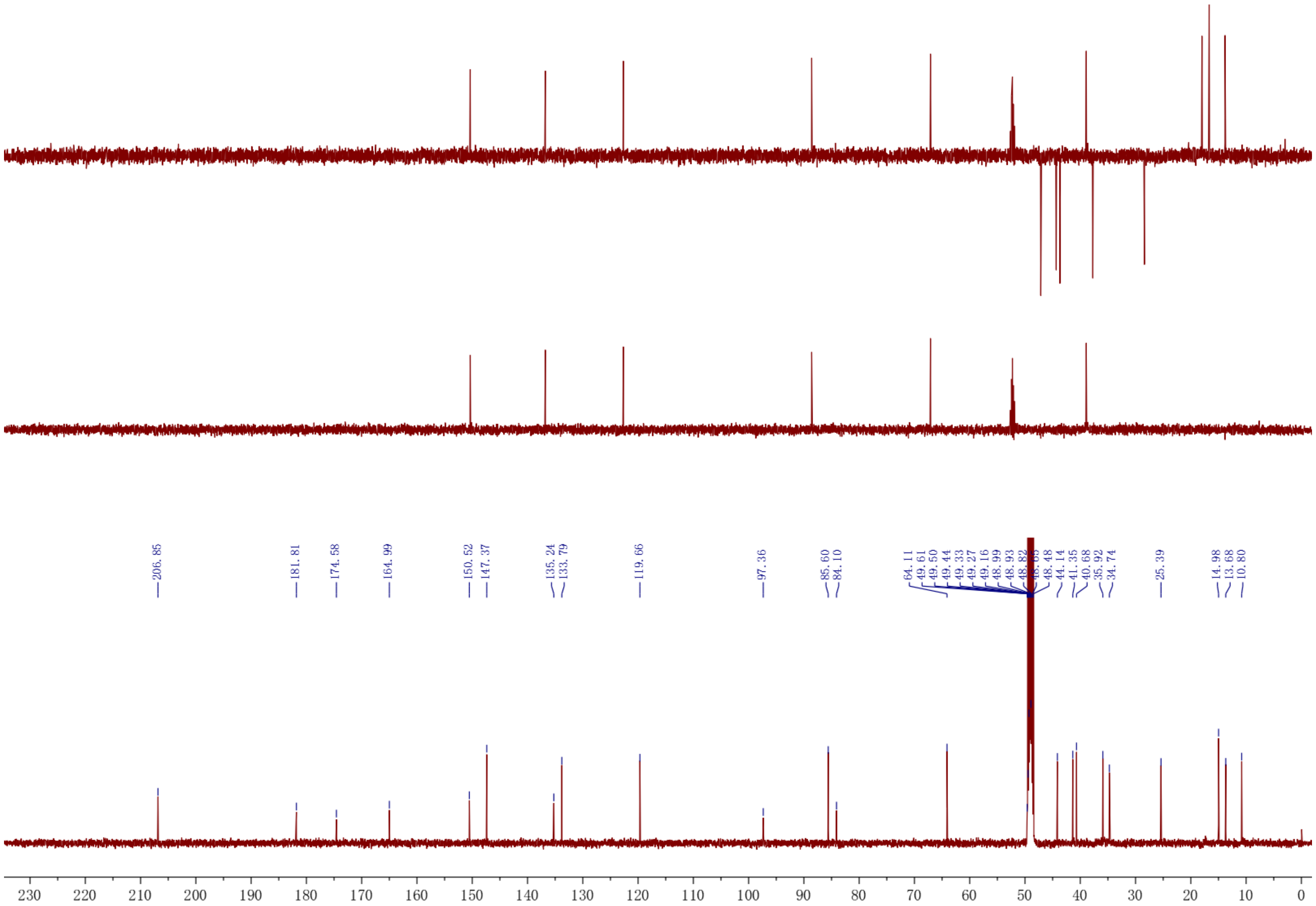

Figure S24. ^{13}C NMR Spectrum of **3** in CD_3OD

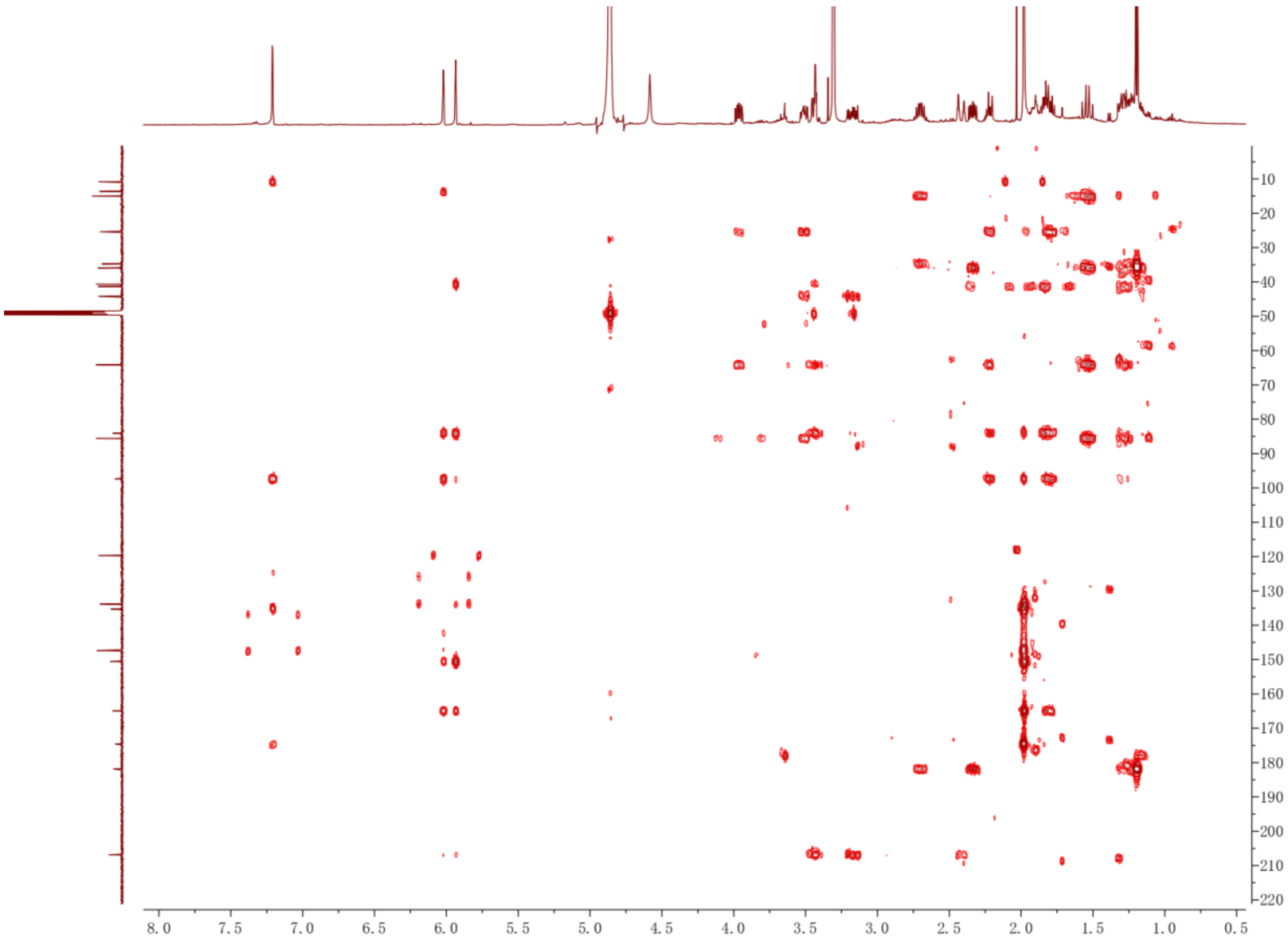

Figure S25. HMBC Spectrum of **3** in CD_3OD

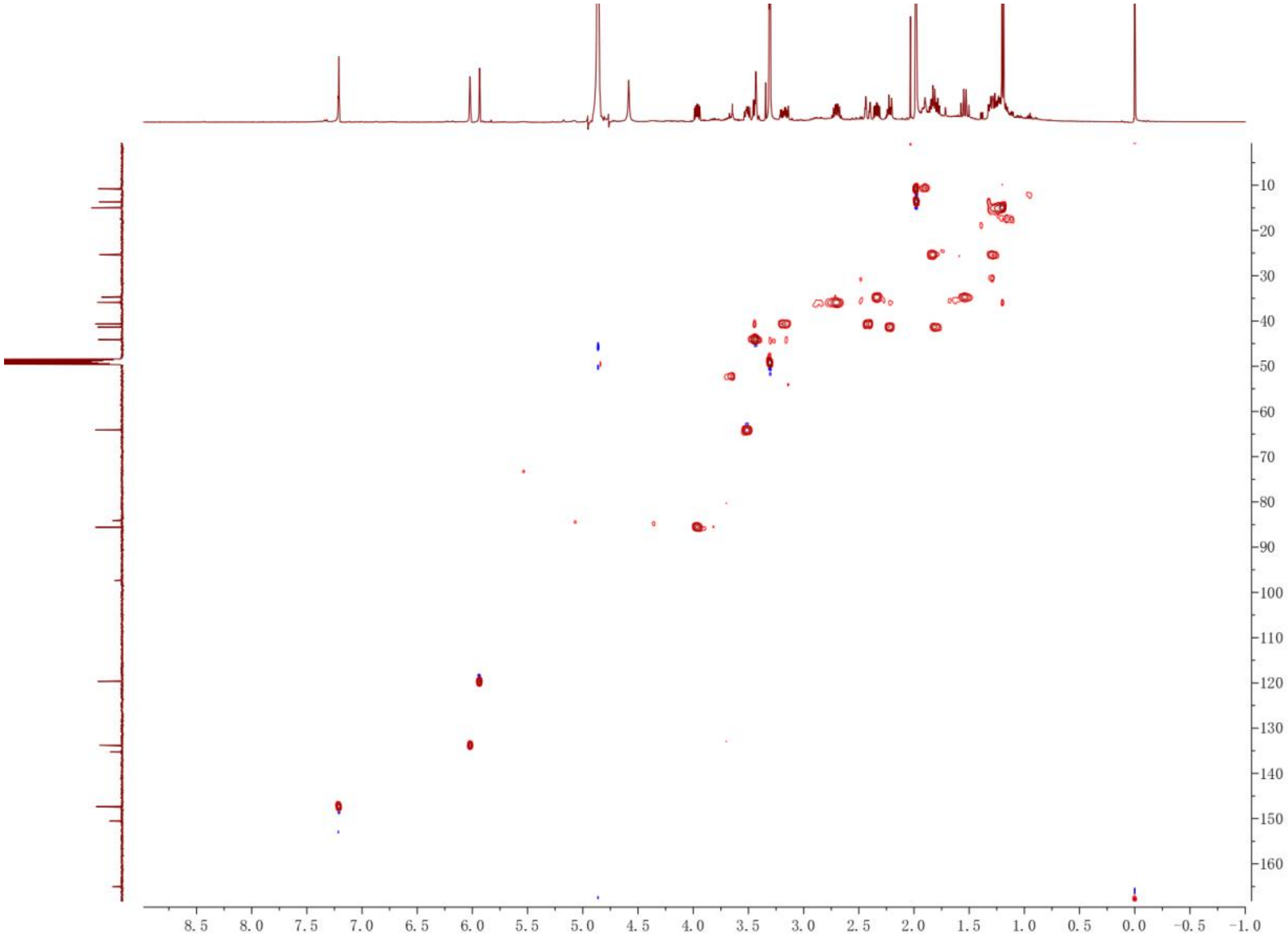

Figure S26. HSQC Spectrum of **3** in CD_3OD

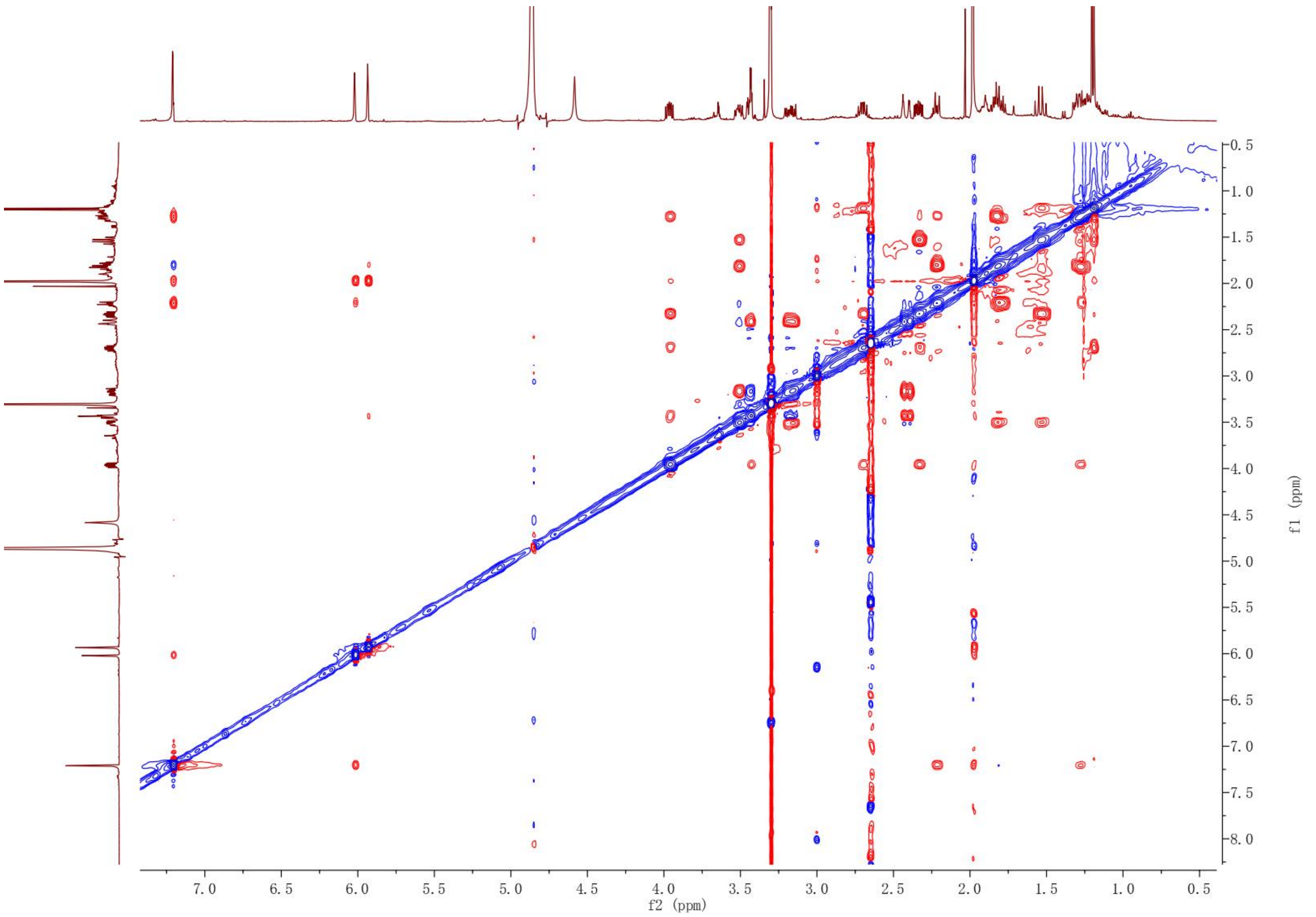

Figure S27. ROESY Spectrum of **3** in CD_3OD

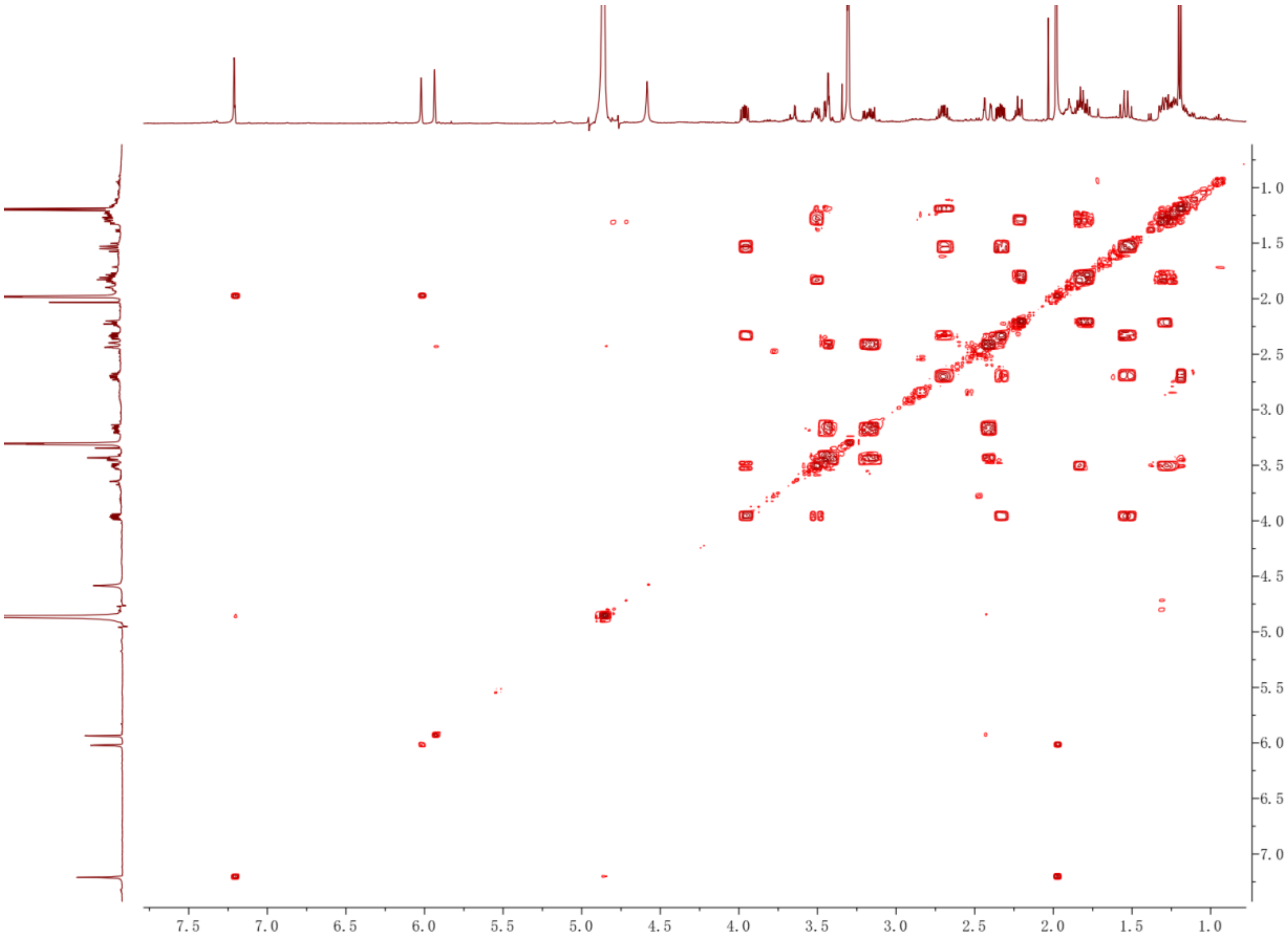

Figure S28. COSY Spectrum of **3** in CD_3OD

Qualitative Analysis Report

**Data Filename**	20221128ESIA5.d	**Sample Name**	wsj-78a
**Sample Type**	Sample	**Position**	
**Instrument Name**	Agilent G6230 TOF MS	**User Name**	KIB
**Acq Method**	ESI.m	**Acquired Time**	11/28/2022 8:53:30 PM
**IRM Calibration Status**	Success	**DA Method**	ESI.m
**Comment**			

**Sample Group**		**Info.**
**Acquisition SW**	6200 series TOF/6500 series	
**Version**	Q-TOF B.05.01 (B5125.2)	

User Spectra

Fragmentor Voltage	Collision Energy	Ionization Mode
250	0	ESI

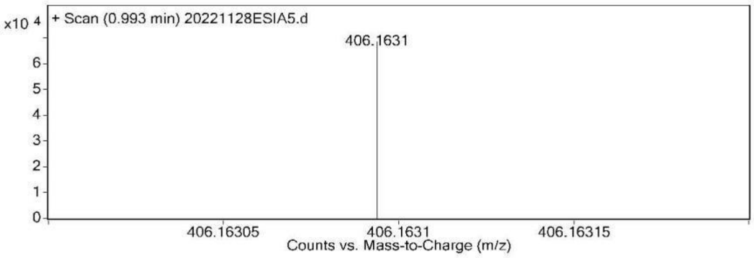

Peak List

m/z	z	Abund	Formula	Ion
131.0848		23578.82		
406.1631	1	68476.35	C22 H25 N Na O5	M+
414.188	1	23665.32		
444.1998	1	446752.59		
445.2026	1	94702.69		
446.2102	1	31171.24		
458.2131	1	46180.79		
460.1743	1	81116.41		
472.2247	1	29761.43		
922.0098	1	114117.12		

Formula Calculator Element Limits

Element	Min	Max
C	0	200
H	0	400
O	0	10
N	1	1
Na	1	1

Formula Calculator Results

Formula	CalculatedMass	Mz	Diff.(mDa)	Diff. (ppm)	DBE
C22 H25 N Na O5	406.1630	406.1631	-0.1	0.1	10.5

--- End Of Report ---

Figure S29. HRESIMS Spectrum of **3**

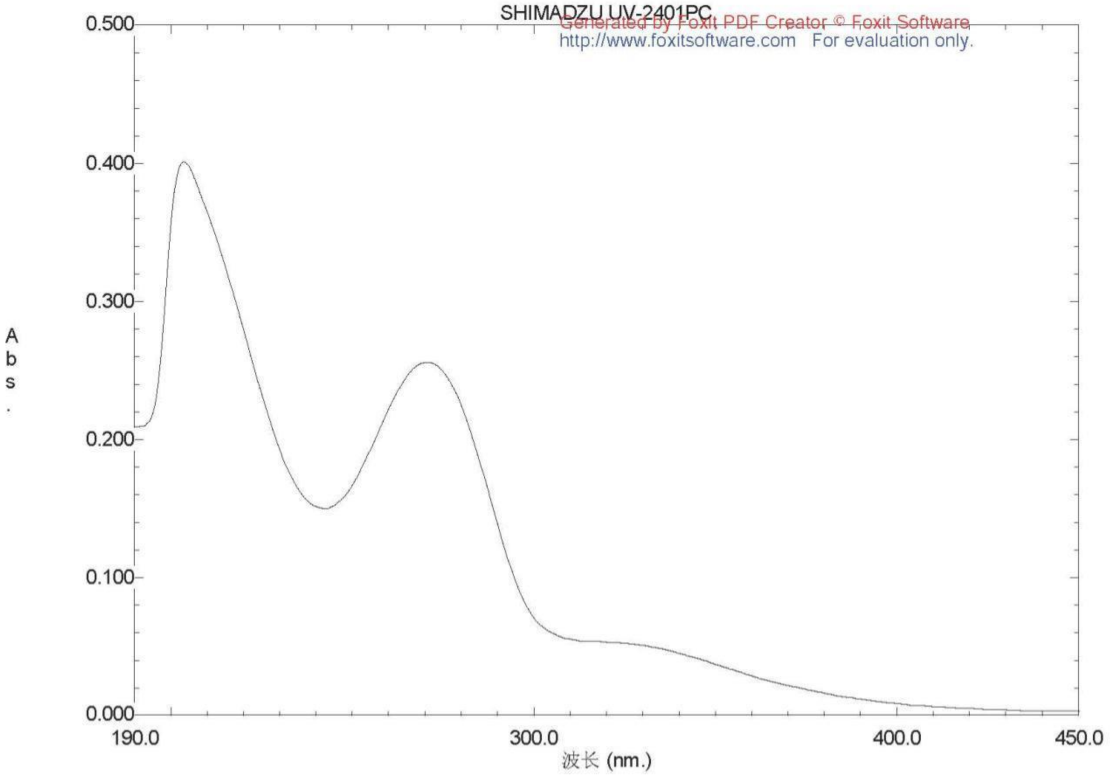

文件名: WSJ-78A

WSJ-78A

样品浓度：0.0600毫克/毫升
溶剂：甲醇

创建于: 17:28 22-11-25
数据: 原始

测量模式: Abs.
扫描速度: 中速
狭缝: 5.0
采样间隔: 0.5

否.	波长 (nm.)	Abs.
1	203.00	0.4001
2	270.50	0.2556
3	322.00	0.0525

Figure S30. UV Spectrum of **3**

Rudolph Research Analytical

This sample was measured on an Autopol VI, Serial #91058
Manufactured by Rudolph Research Analytical, Hackettstown, NJ, USA.

Measurement Date : Friday, 25-NOV-2022

Set Temperature : OFF

Time Delay : Disabled

Delay between Measurement : Disabled

n	Average	Std.Dev.	% RSD	Maximum	Minimum
5	-60.20	1.79	-2.97	-58.00	-62.00

S.No	Sample ID	Time	Result	Scale	OR °Arc	WLG.nm	Lg.mm	Conc.g/100ml	Temp.
1	wsj-78a	04:22:18 PM	-62.00	SR	-0.062	589	100.00	0.100	20.2
2	wsj-78a	04:22:24 PM	-62.00	SR	-0.062	589	100.00	0.100	20.2
3	wsj-78a	04:22:34 PM	-60.00	SR	-0.060	589	100.00	0.100	20.1
4	wsj-78a	04:22:40 PM	-59.00	SR	-0.059	589	100.00	0.100	20.1
5	wsj-78a	04:22:47 PM	-58.00	SR	-0.058	589	100.00	0.100	20.1

Figure S31. Optical Rotation Spectrum of **3**
